# Global, regional, and national burdens of cirrhosis in childhood and adolescence during 2000 to 2021: an age-period-cohort analysis

**DOI:** 10.3389/fpubh.2025.1638207

**Published:** 2025-08-29

**Authors:** Xue-Qi Yang, Xin-Jing Yang, Yu-Xin Tian, Qi An, Yun-Xin Yao, Jing Zuo, Bai-Yun Wu, Jie-Ru Yang, Yu-Chen Fan

**Affiliations:** ^1^Department of Hepatology, Qilu Hospital of Shandong University, Jinan, China; ^2^Institute of Hepatology, Shandong University, Jinan, China

**Keywords:** public health, cirrhosis, global burden of disease, children, APC model

## Abstract

**Background:**

Cirrhosis is a major cause of morbidity and mortality worldwide, but data on the cirrhosis burden and trends in children and adolescents are sparse.

**Methods:**

Data on cirrhosis during 2000–2021, including global-, regional, and national-level numbers of cases, incidence, and prevalence, were collected by the GBD 2021 database. Trends across age groups, periods, and birth cohorts were analysed using the age–period–cohort (APC) model; epidemiological trajectories were predicted using the Bayesian APC (BAPC) model.

**Results:**

From 2000 to 2021, cirrhosis incidence decreased in the 5–9 and 10–14-year age groups but increased in the 15–19 and 20–24-year age groups. In the 15–19- and 20-24-year age groups, the respective proportion of cirrhosis due to hepatitis B decreased from 11 to 4% and from 35 to 23%, while the respective proportion due to metabolic dysfunction-associated fatty liver disease (MAFLD) increased from 87 to 93% and from 55 to 67%. Regionally, the proportion of cirrhosis attributable to HBV decreased over time, while MAFLD became the leading cause among individuals aged 15–24 years. Overall, the effects of cirrhosis among children and adolescents were consistent across socio-demographic index regions and globally, increasing with age but decreasing with period and cohort. However, the period and cohort effects of MAFLD-related cirrhosis increased regionally and globally. The BAPC model predicted that the incidence of cirrhosis among children and adolescents will continue to rise until 2050.

**Conclusion:**

During 2000–2021, the burden associated with hepatitis B declined, while the proportion of cirrhosis caused by MAFLD has been steadily increasing. The APC model revealed a consistent global trend of cirrhosis risk, increasing with age but decreasing by period and cohort.

## Introduction

Cirrhosis represents the terminal stage of progressive liver damage arising from diverse chronic hepatic conditions and remains a major global contributor to liver-related mortality (1). The most common causes are hepatitis B virus (HBV) and hepatitis C virus (HCV) infection, alcohol-related liver injury, and metabolic dysfunction-associated fatty liver disease (MAFLD) (2).

Chronic HBV infection develops in approximately 90% of infected infants and neonates and can lead to progressive liver disease and severe complications, including cirrhosis and hepatocellular carcinoma. Among HBV E antigen-positive children, the incidence of cirrhosis is 1–5% (3). Vertical transmission is the predominant mode of HCV infection in children. However, in high-income countries, increased rates of HCV infection among adolescents are also associated with intravenous drug use (4). Approximately 1–2% of children and adolescents with chronic HCV infection develop cirrhosis, although in a cohort in the United Kingdom, a third of pediatric HCV patients progressed to cirrhosis (5). MAFLD is closely associated with metabolic disturbances including obesity, insulin resistance, type 2 diabetes mellitus, hypertension, hyperglycaemia, and metabolic syndrome. The global incidence of MAFLD-related liver cirrhosis increased by over 100%, reflecting the rapid growth of metabolic disorders in the younger population (6). As the prevalence of obesity and type 2 diabetes mellitus are rising among younger populations, assessment of the disease burden of MAFLD-related cirrhosis in this demographic is crucial (7, 8).

Compared with cirrhosis in adults, cirrhosis in children and adolescents has received less attention, particularly regarding detection and treatment strategies. The global “Countdown to 2030” initiative has emphasized the importance of prioritizing health in children and adolescents (9). A recent study based on GBD 1990–2019 data has analyzed global, regional, and national burdens of cirrhosis in children and adolescents, providing valuable epidemiological insights (10). In this study, we used Global Burden of Disease (GBD) study (2000–2021) data to comprehensively analyse trends and patterns in cirrhosis among children and adolescents by sex, age, region, and country. We used an age–period–cohort (APC) model to examine trends across age groups, periods, and birth cohorts and a Bayesian APC (BAPC) model to predict the future trajectory of cirrhosis among children and adolescents over the next 30 years.

## Materials and methods

### Data sources

The GBD 2021 study offers a systematic evaluation of 371 diseases and injuries, along with 88 associated risk factors, across 204 countries and territories from 1990 to 2021. Its conceptual framework, analytical methodology, cause hierarchy, and detailed approaches are thoroughly documented elsewhere (11). For this study, we extracted data via the Global Health Data Exchange query tool,^1^ we obtained sex-, age-, cause-, region-, and country-disaggregated data, including annual incident cases and incidence rates, prevalent cases and prevalence rates, and death counts and mortality rates (all rates are presented per 100,000 population).

The socio-demographic index (SDI), a composite indicator reflecting a nation or region’s economic, demographic, and developmental characteristics, integrates metrics such as per capita GDP, education attainment, and fertility rates (12). SDI values are scaled from 0 to 1, with higher values denoting greater socio-economic and developmental progress. According to the SDI values reported by GBD 2021, 204 countries and territories were grouped into five tiers: low-SDI, low–middle-SDI, middle-SDI, high–middle-SDI, or high-SDI.

### Study population

The transition from childhood to adulthood encompasses rapid and significant developmental changes, which must be captured through health data analyses over short time intervals. According to the World Health Organization, adolescence spans the ages of 10–19 years (13). However, some researchers have supported extending the definition of adolescence up to 24 years of age, in recognition of continued neurobiological maturation and delayed social transitions into adulthood (14, 15). In this study, we analysed the cirrhosis incidence, prevalence, and mortality rates among children and adolescents aged 5–9, 10–14, 15–19, and 20–24. However, data on cirrhosis attributable to MAFLD were unavailable for the 5–9 and 10–14-year groups; Due to the absence of MAFLD-related data, our APC and BAPC model-based analyses of MAFLD-related cirrhosis were restricted to individuals aged 15–29 years to ensure adequate data structure and model stability, as recommended in previous methodological literature (16).

### Data analysis

We analysed the burdens and etiologies of cirrhosis in children and adolescents, focusing on metrics such as the total number and proportions of cases for incidence, prevalence, and mortality. Age-standardized incidence rates (ASIRs), age-standardized mortality rates (ASMRs), and age-standardized prevalence rates (ASPRs) were calculated based on the GBD 2021 global standard population using the combined 5–24-year age groups. This standardization facilitates accurate comparisons of the liver cirrhosis burden across populations and over time by controlling for differences in age structure. Long-term trends were assessed using joinpoint regression analysis and expressed as the average annual percent change (AAPC). The 95% uncertainty intervals (UIs) were determined using the 2.5^th^ and 97.5^th^ percentiles of 1,000 model draws; statistical significance was defined by the exclusion of zero. These UIs were derived from the GBD 2021 modeling framework and serve as a standard approach to reflect uncertainty. The diagnosis of cirrhosis and its causes was based on ICD-10 codes, following the definitions established in previous GBD studies (1).

### APC model analysis

The APC model surpasses traditional methods used in health and social sciences by capturing both overall temporal trends and specific period effects (17, 18). We used the APC model to analyse trends in cirrhosis incidence due to specific etiologies across different age groups, time periods, and birth cohorts. To ensure consistency, the age intervals matched the time intervals: e.g. 5-year age groups were paired with 5-year time periods. This study covered the age range of 5–24 years, divided into four groups (for MAFLD, the age range was 15–29 years, divided into three groups). The study period was segmented into four 5-year intervals: 2002–2006, 2007–2011, 2012–2016, and 2017–2021.

The APC model estimates both overall temporal and age-specific trends in incidence. In this model, the age effect is depicted by age-specific rates that remain consistent across different birth cohorts. The period and cohort effects are quantified as the relative risk of incidence linked to specific time periods or birth cohorts, respectively. These relative risks are calculated by age-specific rates of periods (cohorts) against an arbitrarily selected reference period (cohort). The reference period and reference cohort were selected as the midpoints of the study range, following standard APC modeling conventions to facilitate estimation and interpretation of relative risks.

### Prediction

To forecast the 30-year disease burden, we employed the Bayesian Age–Period–Cohort (BAPC) model, which incorporates age, period, and cohort effects within a Bayesian generalized linear framework. The model uses a second-order random walk to smooth temporal trends and applies the Integrated Nested Laplace Approximation (INLA) method for efficient estimation of posterior distributions. This approach is particularly suited for large-scale epidemiological data with complex temporal structures and has been widely validated for long-term burden prediction (19).

### Statistics

All calculations and visualizations were performed using R Studio (version 4.4.1) with the packages BAPC (version 0.0.36), INLA (version 24.12.11), and ggplot2 (version 3.5.1).

## Results

### Global trends

**Figure 1:**
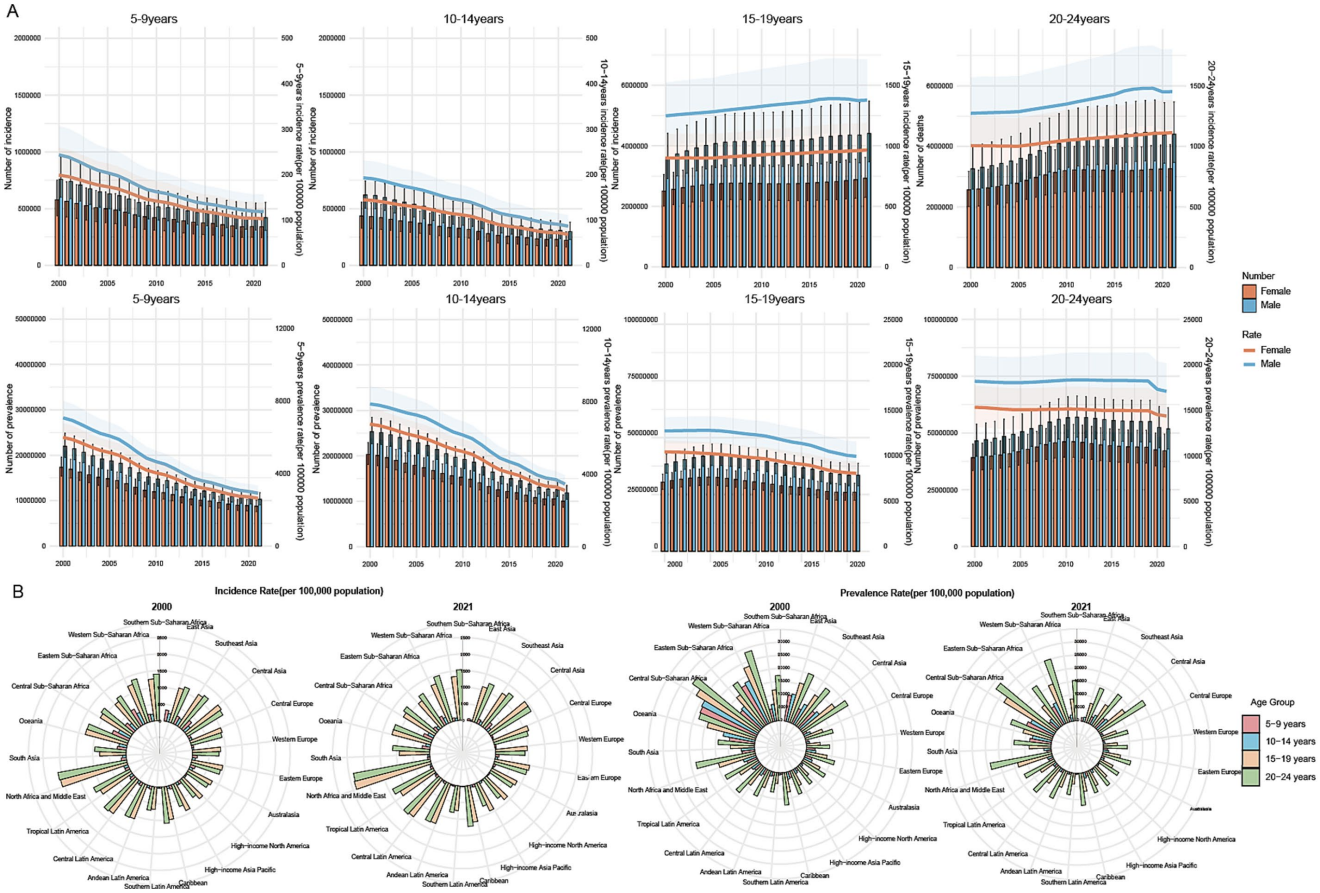
Global, regional, and national burdens of cirrhosis among childhood and adolescence during 2000–2021. **(A)** Counts and rates of cirrhosis incidence and prevalence at the global level by sex, 2000–2021. **(B)** Incidence and prevalence of cirrhosis in different regions in 2000 and 2021 among four age groups: 5–9 years, 10–14 years, 15–19 years, and 20–24 years.

The incidence and prevalence of cirrhosis are presented in Table 1, while mortality data are shown in Supplementary Table 1. From 2000 to 2021, the global numbers of cirrhosis incidence in the 5–9 and 10–14-year groups decreased significantly from 1,335,351 (95% UI: 1,043,359–1,706,085) and 1,056,896 cases (95% UI: 830,108–1,292,430) to 762,124 (95% UI: 558,035–1,018,866) and 518,869 cases (95% UI: 388,687–677,975), representing reductions of 42.9 and 50.9%, respectively. Corresponding prevalence cases in these groups also fell by over 50%. Declining trends in prevalence rates were observed in these groups, as well as the 15–19 and 20–24–year groups (with smaller fluctuations in the latter). However, the incidence in the 15–19 and 20–24–year groups trended upward, from 6,125,687 (95% UI: 4,934,149–7,459,743) and 5,825,581 cases (95% UI: 4,603,344–7,196,156) in 2000 to 7,341,743 (95% UI: 5,779,071–9,112,087) and 7,672,836 cases (95% UI: 6,025,592–9,542,805) in 2021, rising by 19.9 and 31.7%, respectively. Across all four age groups, the incidence and prevalence of cirrhosis were consistently higher in males than in females (Figure 1A). Mortality decreased across all age groups, with the most substantial reductions observed in the 5–9 and 10–14-year groups. Notably, mortality rates were higher among females aged 10–14 and 15–19 (Supplementary Figure 1).

**Table 1:** Global incidence and prevalence of cirrhosis among children and adolescence in 2000 and 2021.

Age group	Incidence					Prevalence				
	2000		2021			2000		2021		
	Number (95%UI)	Rate per 100,000 (95%UI)	Number (95%UI)	Rate per 100,000 (95%UI)	No. change (%)	Number (95%UI)	Rate per 100,000 (95%UI)	Number (95%UI)	Rate per 100,000 (95%UI)	No. change (%)
Age 5–9 years										
Total	1,335,351 (1,043,359, 1,706,085)	221.93 (173.4, 283.55)	762,124 (558,035, 1,018,866)	110.93 (81.22, 148.29)	−42.93	39,336,574 (35,014,912, 44,501,564)	6537.6 (5819.35, 7,396)	19,062,045 (16,707,607, 21,887,494)	2774.46 (2431.78, 3185.7)	−51.54
Female	577,255 (437,622, 751,792)	198.94 (150.82, 259.09)	341,675 (245,159, 461,429)	102.74 (73.71, 138.74)	−40.81	17,357,153 (15,387,511, 19,590,096)	5981.72 (5302.93, 6751.24)	8,744,911 (7,599,380, 10,098,941)	2629.44 (2,285, 3036.57)	−49.62
Male	758,096 (602,132, 954,777)	243.35 (193.28, 306.48)	420,449 (309,865, 554,763)	118.61 (87.41, 156.5)	−44.54	21,979,421 (19,508,743, 24,840,342)	7055.37 (6262.28, 7973.72)	10,317,134 (9,069,349, 11,770,253)	2910.53 (2558.52, 3320.46)	−53.06
Age 10–14 years										
Total	1,056,896 (830,108, 1,292,430)	169.49 (133.12, 207.26)	518,869 (388,687, 677,975)	77.83 (58.31, 101.7)	−50.91	45,642,936 (40,851,512, 51,256,473)	7319.39 (6551.03, 8219.59)	21,869,349 (19,161,300, 24,840,101)	3280.55 (2874.33, 3726.19)	−52.09
Female	435,589 (327,715, 557,160)	144.5 (108.71, 184.83)	221,608 (161,340, 295,492)	86.49 (66.14, 110.74)	−49.12	20,332,341 (18,090,702, 22,915,827)	6744.93 (6001.3, 7601.96)	10,006,854 (8,700,586, 11,401,190)	3098.81 (2694.3, 3530.59)	−50.78
Male	621,307 (503,901, 744,494)	192.87 (156.42, 231.11)	297,261 (227,328, 380,640)	68.63 (49.96,91.5)	−52.16	25,310,596 (22,701,730, 28,483,552)	7856.95 (7047.11, 8841.91)	11,862,495 (10,430,661, 13,495,804)	3451.31 (3034.73, 3926.51)	−53.13
Age 15–19 years										
Total	6,125,687 (4,934,149, 7,459,743)	1076.95 (867.47, 1311.49)	7,341,743 (5,779,071, 9,112,087)	1176.6 (926.16, 1460.32)	19.85	68,836,746 (61,614,124, 76,567,306)	12102.16 (10832.36, 13461.27)	59,490,140 (51,198,019, 68,602,474)	9533.98 (8205.08, 10994.34)	−13.58
Female	2,505,467 (2,010,703, 3,053,580)	899.36 (721.76, 1096.11)	2,930,518 (2,298,803, 3,616,766)	965.1 (757.06, 1191.1)	16.96	30,417,578 (27,169,043, 33,902,915)	12102.16 (10832.36, 13461.27)	26,014,127 (22,408,883, 29,961,104)	8567.14 (7379.84, 9866.99)	−14.48
Male	3,620,220 (2,914,489, 4,414,711)	1247.43 (1004.25, 1521.19)	4,411,225 (3,472,403, 5,483,022)	1377.09 (1084.01,1711.68)	21.85	38,419,169 (34,419,995, 42,756,183)	13238.19 (11860.19, 14732.61)	33,476,014 (28,798,795, 38,707,022)	10450.48 (8990.35, 12083.49)	−12.87
Age 20–24 years										
Total	5,825,581 (4,603,344, 7,196,156)	1139.98 (900.81, 1408.18)	7,672,836 (6,025,592, 9,542,805)	1284.89 (1009.04, 1598.04)	31.71	85,692,969 (73,420,633, 98,847,998)	16768.84 (14367.32, 19343.08)	94,034,631 (77,901,800, 110,589,850)	15747.02 (13045.42, 18519.36)	9.73
Female	2,567,241 (2,011,738, 3,174,818)	1006.59 (788.78, 1244.81)	3,267,856 (2,541,279, 4,062,684)	1112.45 (865.11, 1383.03)	27.29	39,110,848 (33,505,438, 45,131,799)	15334.9 (13137.09, 17695.65)	42,202,022 (35,027,112, 49,522,338)	14366.55 (11924.04, 16858.55)	7.9
Male	3,258,340 (2,587,054, 4,016,175)	1272.89 (1010.64, 1568.94)	4,404,980 (3,468,415, 5,468,996)	1451.84 (1143.16, 1802.53)	35.19	46,582,121 (39,754,598, 53,824,036)	18197.52 (15530.32, 21026.61)	51,832,609 (42,970,796, 61,122,514)	17083.57 (14162.8, 20145.45)	11.27

### Regional trends

Regionally, the cirrhosis incidence and prevalence trends in the 5–9 and 10–14-year groups declined across all regions in 2021 compared with 2000. The most substantial decreases in the incidence rate occurred in East Asia, with respective decreases from 332.36 and 249 to 58.13 and 33.66 (all per 100,000), corresponding to AAPCs of −10.80% and −9.81%, respectively. Similarly, the prevalence rate declined from 10,062.43 and 10,733.35 to 1,358.32 and 1,720.24, with AAPCs of −9.60% and −9.70%. In addition to East Asia, a decrease in prevalence was observed in the 5–9-year group in South Asia (AAPC:–5.15%) and Eastern Sub-Saharan Africa (AAPC: −5.22%). In the 10–14-year groups, significant decreases in prevalence were recorded in Central Europe (AAPC: −5.32%) and Australasia (AAPC: −5.35%).

In the 15–19 and 20–24-year groups, an increasing trend in cirrhosis incidence was reported in most regions, excepting Oceania, which reported respective declines from 1,301.47 and 1,293.66 to 1,214.08 and 1,207.85, respectively. In the 15-19-year groups, the largest incidence increases were observed in North Africa and the Middle East (AAPC: 0.67%), South Asia (AAPC:0.73%) and Andean Latin America (AAPC: 0.78%). The 15–19-year groups experienced decreases in prevalence in all 21 regions: the most substantial decrease was in East Asia, from 8209.31 to 7441.29 (AAPC: −3.91%). In the 20–24-year group, regional prevalence trends varied significantly. The majority of regions experienced slight declines. However, a few regions, including Central Asia (AAPC: 0.02%), Western Europe (AAPC: 0.08%), North Africa and the Middle East (AAPC: 0.08%), South Asia (AAPC: 0.15%), high-income North America (AAPC: 0.24%), Andean Latin America (AAPC: 0.26%), and Southern Latin America (AAPC: 0.44%), recorded marginal increases in prevalence (Figure 1B; Supplementary Table 2).

### National trends

**Figure 2:**
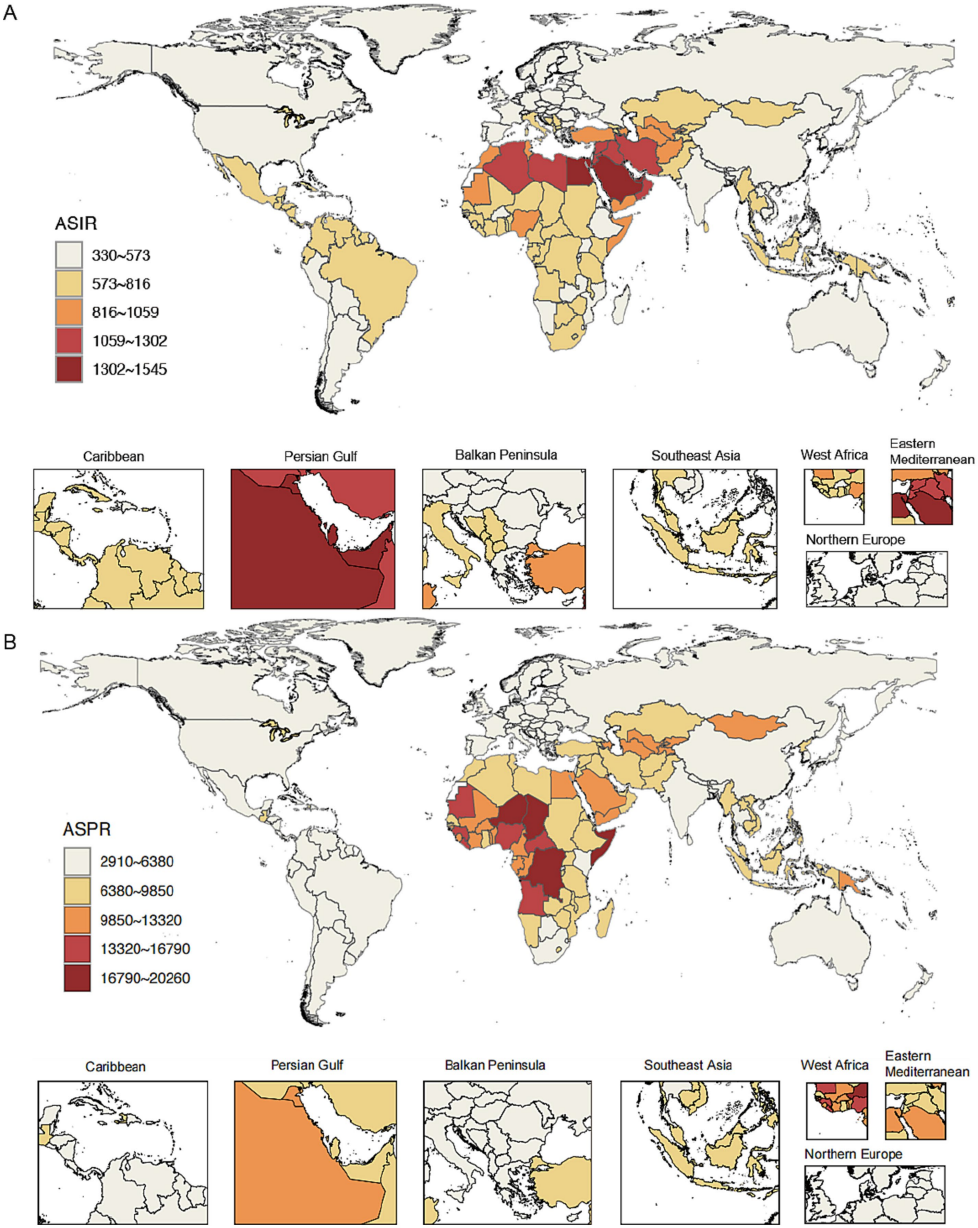
Age-standardized incidence rate (ASIR) and age-standardized prevalence rate (ASPR) of cirrhosis in countries and territories in 2021. **(A)** ASIR of cirrhosis in countries and territories in 2021. **(B)** ASPR of cirrhosis in countries and territories in 2021.

Among individuals aged 5–24 years, Egypt reported the highest ASIR of cirrhosis in 2021, reaching 1573.74 (all per 100,000). Other Eastern Mediterranean countries, such as Qatar and Kuwait (1458.92 and 1,417.46, respectively), also ranked highly. Canada recorded the lowest ASIR in 2021 at 331.36, followed by Finland at 344.6 (Figure 2A).

In 2021, Somalia had the highest age-standardized prevalence rate of 20,259.33, followed by Chad (18,500.38) and Democratic Republic of the Congo (18,394.6). Eight of the 10 countries with the lowest age-standardized prevalence rates were located in Europe: Norway (2,990.26), Germany (3,109.09), Greece (3,122.76), the Netherlands (3,147.41), Cyprus (3,172.37), Luxembourg (3,225.13), Belgium (3,251.64), and Sweden (3,286.03). The others were Uruguay (2,910.63) and Argentina (2,912.43; Supplementary Table 3; Figure 2B).

To further explore the national burden of liver cirrhosis, we examined age-specific crude incidence, prevalence, and mortality rates in 2021 for the 5–9, 10–14, 15–19, and 20–24 age groups across countries and territories (Supplementary Figures 2–4). Although crude rates naturally increased with age, the countries with the highest burden were largely consistent across all age groups. For instance, Egypt, Somalia, and Chad consistently ranked among the top in multiple indicators, suggesting a concentration of burden in specific regions.

### Cirrhosis by specific etiology

**Figure 3:**
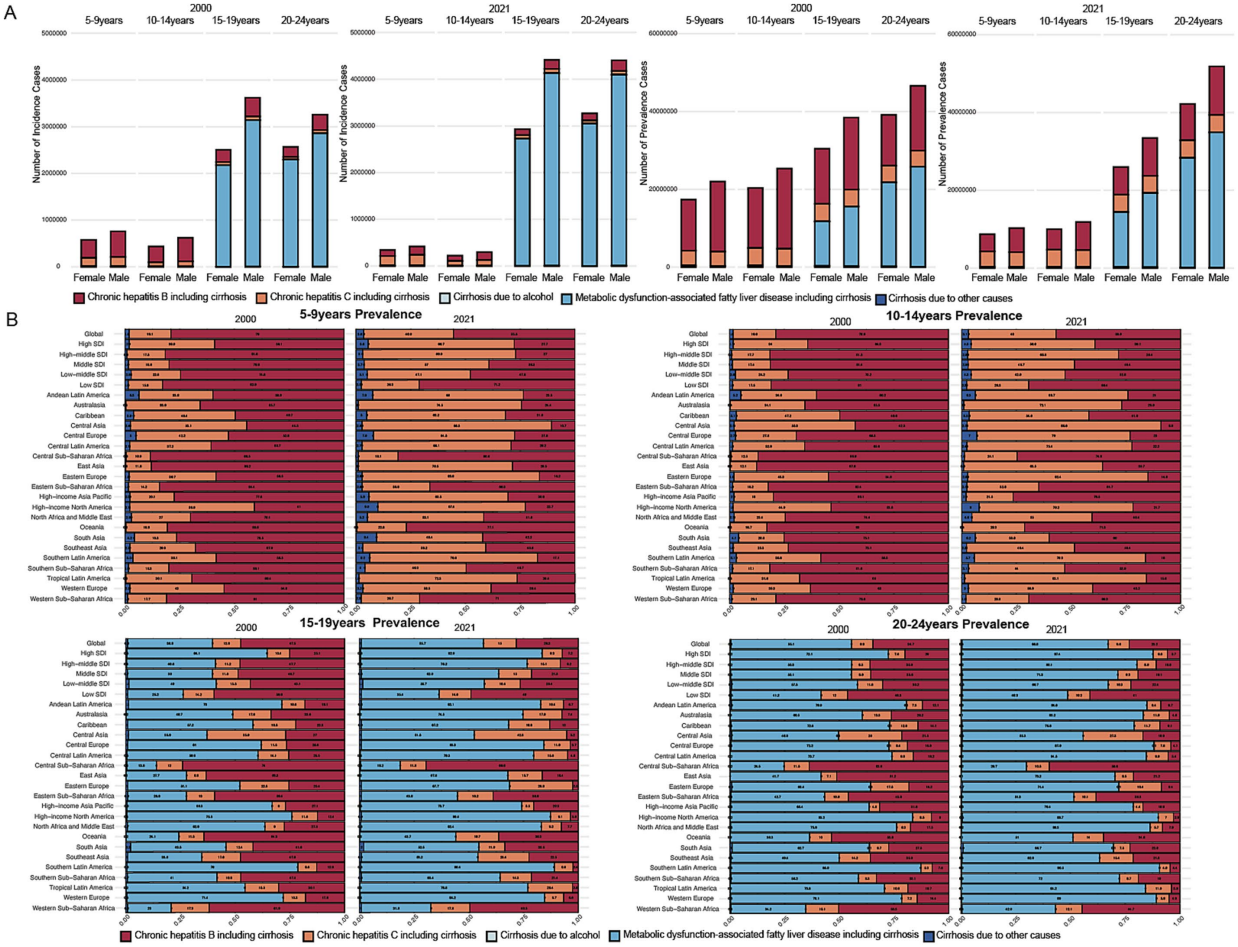
Contribution of specific causes among childhood and adolescents in different age groups and regions in 2000 and 2021. **(A)** Contributions of different causes to the incidence and prevalence rates across four age groups in the years 2000 and 2021. **(B)** Contributions of different causes to the prevalence rates across regions across four age groups in the years 2000 and 2021.

With regard to incidence, the 5–9 and 10–14-year age groups lacked available data on cirrhosis attributable to MAFLD. In these age groups, HBV and HCV remained the predominant causes of cirrhosis. Compared with 2000, the number of incident cirrhosis cases due to HBV declined markedly by 2021, whereas HCV-related incidence showed minimal change. In the 15–19 and 20–24-year groups, MAFLD was the primary risk factor for cirrhosis, followed by HBV and HCV. Males consistently had significantly higher incidence and prevalence rates of cirrhosis than females across all causes and age groups. A similar pattern was observed for prevalence, with MAFLD emerging as the dominant cause in the older age groups (Figure 3A). Across all age groups, cirrhosis due to other causes represented the primary contributor to cirrhosis-related mortality in both 2000 and 2021, with this pattern being particularly pronounced in the 15–24-year groups (Supplementary Figure 5).

Compared with 2000, the proportion of cirrhosis incidence attributable to HCV among individuals aged 5–9 and 10–14 years increased notably across most of the 21 global regions by 2021. In 2021, the Central region reported that HCV accounted for 92 and 87% of cirrhosis incidence in the 5–9 and 10–14-year age groups, respectively. Similarly, in Eastern Europe, HCV contributed to 88 and 86% of cirrhosis cases in the same age groups. Among individuals aged 15–19 and 20–24 years, MAFLD remained the leading cause of cirrhosis incidence in both 2000 and 2021 across nearly all of the 21 global regions(Supplementary Table 4; Supplementary Figure 6). In terms of prevalence, during 2000–2021, in the 15–24-year groups, the proportion of cirrhosis caused by HBV decreased regionally and globally, while the proportion caused by MAFLD steadily increased (Figure 3B). Regarding its prevalence in low-SDI regions, HBV remained a leading cause of cirrhosis aged 15–24 years in 2021, accounting for nearly 50% of cases. For example, in 2021, the proportion of cirrhosis prevalence attributable to HBV among individuals aged 15–24 years exceeded 50% in several low-SDI regions in Africa (Figure 3B; Supplementary Table 5). Regarding mortality, cirrhosis due to other causes remained the leading cause of death among individuals aged 5–24 years. However, in East Asia, HBV-related cirrhosis accounted for a disproportionately high share of deaths in this age group, making it a prominent contributor in that region (Supplementary Figure 6; Supplementary Table 6).

### Age, period, and cohort effects on cirrhosis incidence

The APC model-derived age, period, and cohort effects of cirrhosis are demonstrated in Supplementary Figure 7. Overall, the patterns are similar across different SDI levels. The risk of developing cirrhosis increases steadily with age. Additionally, the incidence was lower in high-SDI regions than elsewhere. Over time, the temporal influence displayed a sustained downward trajectory worldwide and within all SDI regions. Regarding the cohort effect, the comparative prevalence risk among sequential birth cohorts initially rose before diminishing, a phenomenon observed globally and across all SDI regions. In high-SDI regions, the 2002–2006 birth cohort demonstrated a generational risk ratio of 1.013 (95% CI: 0.927–1.107) relative to the 1997–2001 cohort. Other SDI regions exhibited progressive reductions in incidence risk across successive birth cohorts.

We also used the APC model to analyse the age, period, and cohort effects of cirrhosis caused by HBV, HCV, and MAFLD. For HBV-related cirrhosis, the model revealed a consistent declining trend in age effects across regions, with high-SDI regions exhibiting low incidence rates across all age groups and a narrowing gap between these groups. Similarly, period and cohort effects trended downwards, reflecting a gradual reduction in the incidence risk of HBV-related cirrhosis over time and across successive cohorts (Supplementary Figure 8).

For HCV-related cirrhosis, APC model analysis revealed distinct patterns across SDI regions. Globally, the incidence risk decreased with age. The period effect indicated that the period risk of HCV-associated cirrhosis has remained stable globally, with a gradual decrease in the risk of developing cirrhosis with age observed in high-, high–middle-, and middle-SDI regions. However, in low-SDI regions, an entirely opposite trend and consistently less favorable outcomes were observed throughout most of the study period. Compared with the reference period (2007–2011), those in 2012–2016 and 2017–2021 had relative period risks of 1.062 (95% CI: 1.056–1.069) and 1.164 (95% CI: 1.156–1.172), respectively. Regarding cohort effects, a distinct global incidence risk pattern was observed across successive birth cohorts, with an overall downward trend. The trends in higher–middle-SDI regions closely mirrored the global pattern. However, the trend of incidence risk in low-SDI regions progressively worsened across successive cohorts (Supplementary Figure 9).

**Figure 4:**
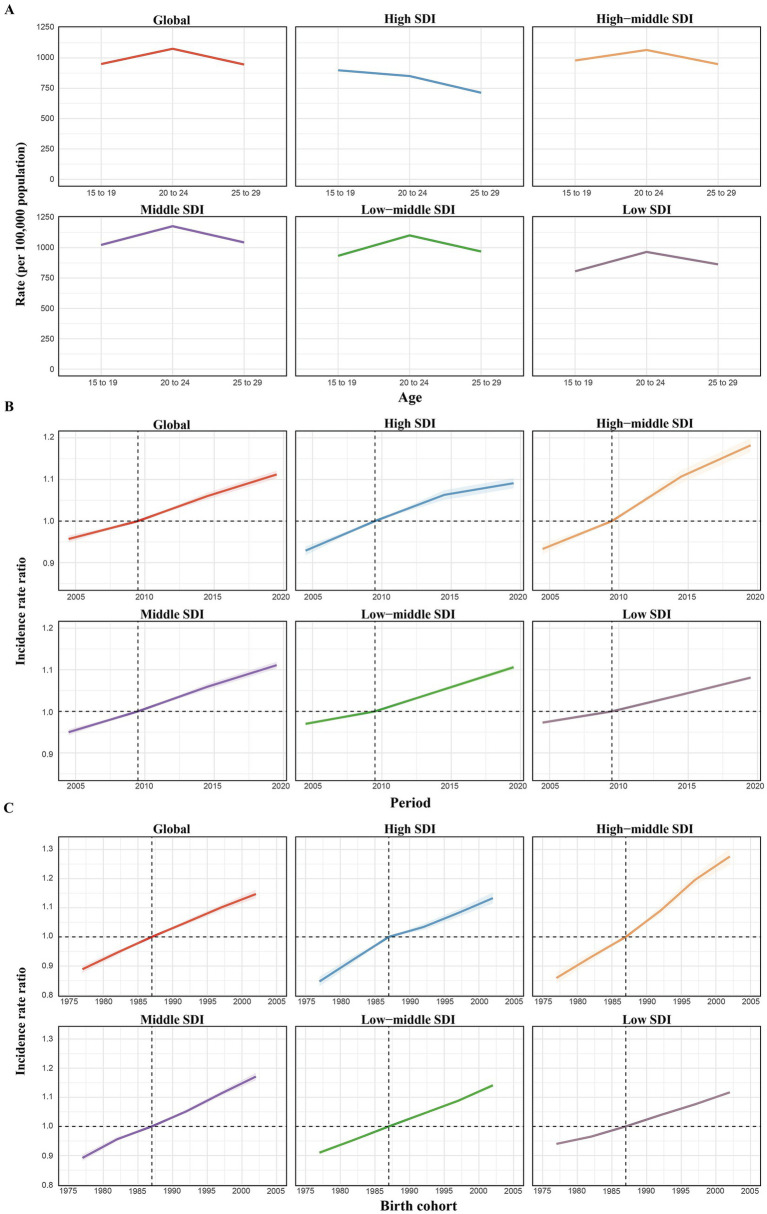
Age, period, and birth cohort effects on MAFLD-related cirrhosis incidence in childhood and adolescence according to age–period–cohort (APC) models. **(A)** Age effects: longitudinal age-specific rates, adjusted for cohort variations and period-specific deviations. **(B)** Period effects: relative risk of cirrhosis incidence across different time periods, comparing age-specific rates from 2002–2006 to 2017–2021, with 2007–2011 as the baseline. **(C)** Birth cohort effects: cohort relative risk of incidence, comparing age-specific rates between the 1978–1987 and 2007–2016 birth cohorts, using 1997–2006 as the reference. The trend line and shaded regions represent incidence rates or rate ratios, with 95% confidence intervals (CIs). SDI, socio-demographic index.

The APC model analysis of MAFLD-related cirrhosis revealed consistent patterns across global and SDI regions, with some notable differences. Across regions excluding high-SDI areas, age-related patterns exhibited comparable trajectories, peaking in the 20–24-year cohort and subsequently diminishing with advancing age. In high-SDI regions, however, the risk steadily decreased with age. Both the period and cohort effects showed a continuous upward trend globally and regionally, reflecting unfavorable period risks and cohort effects for MAFLD-related cirrhosis (Figure 4).

### Global disease burden prediction for cirrhosis until 2050

**Figure 5:**
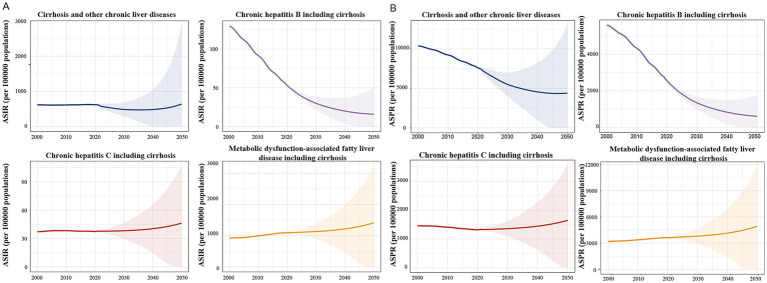
Prediction of the global ASIR and ASPR of cirrhosis and the number of cases of cirrhosis caused by different etiologies in children and adolescents from 2022 to 2050. **(A)** Prediction of the global ASIR of cirrhosis in children and adolescents from 2022 to 2050. **(B)** Prediction of the global ASPR of cirrhosis in children and adolescents from 2022 to 2050.

The BAPC model predictions of future trends from 2022 to 2050 indicated a steady decline in the ASIR and ASPR for HBV-induced cirrhosis. In contrast, the ASIR and ASPR for HCV-induced cirrhosis was predicted to initially remain stable. Regarding MAFLD-induced cirrhosis, a concerning upward trend in the ASIR and ASPR throughout the forecast period, with acceleration after 2040, was predicted. These projections highlight contrasting trends between cirrhosis etiologies, with a declining burden of HBV but increasing rates of HCV and MAFLD (Figure 5).

With regard to mortality, the BAPC model projected a continued decline in the ASMR for cirrhosis caused by HBV, HCV, and other causes from 2022 to 2050. In contrast, the ASMR for MAFLD-induced cirrhosis was predicted to increase steadily throughout the forecast period (Supplementary Figure 10).

In addition, we also conducted projections of crude incidence, prevalence, and mortality rates across different age groups. The trends observed in these crude rates were consistent with those of the age-standardized rates (Supplementary Figure 11).

## Discussion

This study is the first to use GBD 2021 data and the APC model to analyse the global burden and trends of cirrhosis among children and adolescents, yielding an overview of the global cirrhosis incidence and prevalence stratified by developmental stage and sex. Although the global cirrhosis incidence trend declined in the 5–9 and 10–14-year groups during 2000–2021, the incidence trended upwards in the 15–19 and 20–24-year groups. Furthermore, the overall burden of cirrhosis remained higher in males (vs. females) across all age groups. A retrospective cohort study in China of 16,738 patients with HBV-related cirrhosis during 2008–2015 reported 1.8 times more male than female patients (20). The consistently higher burden of cirrhosis observed in males across all age groups is consistent with findings from previous global studies (1). This disparity may be explained by a combination of behavioral, environmental, and biological factors. Men are generally more exposed to key risk factors such as alcohol consumption and hepatitis virus infections, while estrogen is thought to exert protective effects against hepatic fibrosis in female (21, 22). These findings underscore the importance of developing gender-sensitive public health strategies to address gender-specific disparities in the cirrhosis burden. The age effect on cirrhosis risk was consistent regionally and globally, with the risk increasing steadily with age. Similarly, the period and cohort effect trends uniform globally and regionally, with the former declining gradually and the latter initially increasing, then decreasing. The BAPC model predicted a gradual decline in the ASIR of cirrhosis among children and adolescents by 2040, followed by a potential rebound, indicating the need for long-term monitoring and targeted interventions in high-risk populations and regions to address these patterns.

The HBV-related cirrhosis incidence and prevalence rates among children and adolescents declined steadily, particularly in high-SDI regions, which exhibited relatively low incidence rates. The APC model analysis of HBV-related cirrhosis indicated gradual decreases in risk over time and across successive cohorts. However, in regions such as Central and Western Sub-Saharan Africa, HBV remains a leading cause of cirrhosis. The significant decline in HBV incidence is primarily attributable to the widespread implementation of vaccination. This phenomenon may be partly attributed to the low coverage of hepatitis B vaccination in low-SDI regions. According to the WHO Global Hepatitis Report, although many countries have included the hepatitis B vaccine in their routine immunization schedules, the global coverage of the birth dose remained as low as 39% in 2015, with particularly low rates reported in the African region (23). This reflects ongoing challenges in timely immunization and weak health system infrastructure. In May 2016, the WHO proposed to eliminate viral hepatitis as a public health threat by 2030 (23). Timely antiviral treatment is crucial for significantly reducing HBV-related deaths (24).

The analysis of HCV-related cirrhosis revealed significant regional differences. Globally, between 2000 and 2021, the incidence and prevalence of HCV-related cirrhosis among individuals aged 5–24 years remained relatively stable. Notably, in 2021, HCV accounted for a substantial proportion of cirrhosis cases in the 5–14-year age group in regions such as Central Sub-Saharan Africa and Eastern Europe. In 2018, the WHO recommended direct antiviral agents for treating HCV in individuals aged ≥12 years, regardless of disease stage (25), indicating the importance of expanding direct antiviral agents access and integrating HCV treatment into broader public health strategies, particularly in resource-limited settings. Despite available treatment options, these disparities in the HCV-related cirrhosis burden highlight the urgent need for targeted interventions to address inequities and reduce the global disease burden. In high-SDI regions, efforts should focus on harm reduction strategies to prevent HCV transmission among adolescent drug users. In low-SDI regions, addressing economic and healthcare barriers is crucial to ensuring equitable access to HCV treatment. The WHO has highlighted that access to diagnostics and treatment remains particularly limited in low- and middle-income countries, contributing to persistently low treatment coverage (26).

MAFLD-related cirrhosis is a major risk factor among individuals aged 15–19 and 20–24 and is steadily increasing worldwide, mirroring rising trends in adults (27). Excepting high-SDI regions, the age effect on MAFLD-related cirrhosis risk is consistent globally and regionally, with an initial increase with age followed by a decrease. However, the trends for the period and cohort effects on MAFLD-related cirrhosis are unfavorable and consistent regionally and globally. The growing burden of MAFLD-related cirrhosis is closely linked to the high overall prevalence of obesity and type 2 diabetes mellitus (28), and MAFLD is now a leading cause of liver disease in both adults and children. Dietary strategies must be adjusted to meet the specific nutritional requirements of different age groups. Age-appropriate interventions would ensure that children and adolescents receive adequate nutrition to support growth while reducing the risk of MAFLD (29).

This study is the first to analyse cirrhosis incidence associated with different etiologies among children and adolescents using the APC model and thus increases the understanding of dynamic disease trends, while offering valuable insights for epidemiology and public health. However, several limitations remain. First, as a secondary analysis based on GBD 2021 data, our findings are subject to the inherent limitations of the database. Specifically, the GBD lacks original epidemiological data for MAFLD-related cirrhosis and alcohol-associated liver disease in certain pediatrics age groups, relying instead on modeled estimates, which may introduce bias or uncertainty. To mitigate these limitations, we employed scientifically robust statistical methods, including APC and BAPC models, to enhance the accuracy and interpretability of temporal trends. Second, in the APC model, we selected the midpoint period and cohort as reference categories, following standard practice. While this approach does not affect the overall trends in age, period, and cohort effects, we did not conduct sensitivity analyses using alternative reference categories. This may affect the accuracy of the estimated effects. Third, autoimmune hepatitis, the sixth leading cause of cirrhosis, and genetic metabolic liver diseases, as significant causes of pediatric cirrhosis, are categorized as ‘other causes’ in the GBD 2021 database and not listed separately. Although detailed analyses of cirrhosis caused by these two conditions are needed, our overall understanding of the burden of cirrhosis in children and adolescents is not affected. Finally, although the GBD database includes estimates of risk factors, only behavioral risks—namely high alcohol use and drug use—are available for cirrhosis in the 15–24-year age group. Risk factor data are not available for younger age groups, such as 5–14 years, thus limiting the ability to comprehensively analyze age-specific attributable risks in this study. Future research should focus on regional differences in cirrhosis risk factors and causes and design tailored interventions by region, enabling healthcare providers to better address the unique challenges faced by different populations.

From 2000 to 2021, global cirrhosis burden exhibited divergent trends across age groups and regions. While incidence and prevalence declined in children aged 5–14 years, adolescents and young adults (15–24 years) experienced rising rates. MAFLD emerged as the leading cause of cirrhosis in older age groups, with HBV and HCV also playing significant roles. The age-period-cohort analysis indicated that while the risk of cirrhosis increases with age, there has been a general decline in incidence over time. Future projections suggest a decreasing burden of HBV-related cirrhosis but an increasing trend for MAFLD, highlighting the need for targeted interventions and public health strategies.

## Data Availability

Publicly available datasets were analyzed in this study. This data can be found at: https://ghdx.healthdata.org/gbd-2021.

## Footnotes

^1^ http://ghdx.healthdata.org/gbdresults-tool
