# Supplementary material for: Global, regional, and national burdens of cirrhosis in childhood and adolescence during 2000 to 2021: an age-period-cohort analysis
Source: Front Public Health. 2025 Aug 29;13:1638207. doi: 10.3389/fpubh.2025.1638207 (PMC12425971; doi:10.3389/fpubh.2025.1638207)
Supplement: Supplementary file 1 [file Table_1.docx]

**Supplements**

**Supplementary Table legends:**

**Supplementary Table 1. Global deaths of cirrhosis among children and adolescence in 2000 and 2021.**

**Supplementary Table 2. Incidence, prevalence, and mortality rates of cirrhosis (per 100,000 population) among individuals aged 5–24 years from 2000 to 2021, presented at the global level, across five SDI regions and 21 GBD regions.**

**Supplementary Table 3.Age-standardised incidence rate (ASIR) and age-standardised prevalence rate (ASPR) of cirrhosis among children and adolescents at the national level in 2021.**

**Supplementary Table 4. Proportional contributions of different etiologies to cirrhosis incidence among individuals aged 5–24 years in 2000 and 2021, stratified by global, five SDI regions, and 21 GBD regions.**

**Supplementary Table 5. Proportional contributions of different etiologies to cirrhosis prevalence among individuals aged 5–24 years in 2000 and 2021, stratified by global, five SDI regions, and 21 GBD regions.**

**Supplementary Table 6. Proportional contributions of different etiologies to cirrhosis deaths among individuals aged 5–24 years in 2000 and 2021, stratified by global, five SDI regions, and 21 GBD regions.**

**Supplementary Table 1. Global deaths of cirrhosis among children and adolescence in 2000 and 2021.**

| **Deaths** | | | | |
| --- | --- | --- | --- | --- |
| **2000** | | **2021** | |  |
| **Number(95%UI)** | **Rate per 100,000(95%UI)** | **Number(95%UI)** | **Rate per 100,000(95%UI)** | **No. change (%)** |
|  |  |  |  |  |
| **6702 (5684,7710)** | **1 (1,1)** | **3532 (2830,4236)** | **1 (0,1)** | **-47.29%** |
| **2906 (2359,3572)** | **1 (1,1)** | **1405 (1134,1667)** | **0 (0,0)** | **-51.66%** |
| **3796 (3098,4463)** | **1 (1,1)** | **2127 (1622,2623)** | **1 (0,1)** | **-43.97%** |
|  |  |  |  |  |
| **6524 (5705,7430)** | **1 (1,1)** | **4319 (3782,4988)** | **1 (1,1)** | **-33.79%** |
| **3425 (2948,4059)** | **1 (1,1)** | **2346 (1987,2730)** | **1 (1,1)** | **-31.49%** |
| **3099 (2649,3649)** | **1 (1,1)** | **1973 (1641,2279)** | **1 (0,1)** | **-36.34%** |
|  |  |  |  |  |
| **13896 (12591,15667)** | **2 (2,3)** | **11106 (9895,12467)** | **2 (2,2)** | **-20.09%** |
| **7141 (6270,8423)** | **3 (2,3)** | **5830 (4972,6794)** | **2 (2,2)** | **-18.36%** |
| **6755 (5918,7694)** | **2 (2,3)** | **5275 (4601,6066)** | **2 (1,2)** | **-21.91%** |
|  |  |  |  |  |
| **18761 (17407,20576)** | **4 (3,4)** | **16805 (15025,18949)** | **3 (3,3)** | **-10.43%** |
| **8359 (7397,9339)** | **3 (3,4)** | **7448 (6138,8765)** | **3 (2,3)** | **-10.90%** |
| **10402 (9259,11656)** | **4 (4,5)** | **9356 (8179,10722)** | **3 (3,4)** | **-10.06%** |

**Supplementary Table 2. Incidence, prevalence, and mortality rates of cirrhosis (per 100,000 population) among individuals aged 5–24 years from 2000 to 2021, presented at the global level, across five SDI regions and 21 GBD regions.**

| **Characteristics** | **Incidence Rate per100,000(95% UI)** | | | **Prevalence Rate per100,000(95% UI)** | | | **Deaths Rate per100,000(95% UI)** | | |
| --- | --- | --- | --- | --- | --- | --- | --- | --- | --- |
|  | **2000** | **2021** | **2000-2021 AAPC(95%CI)** | **2000** | **2021** | **2000-2021 AAPC(95%CI)** | **2000** | **2021** | **2000-2021 AAPC(95%CI)** |
| **5-9years** |  | | | | | | | | |
| Global | 221.93 (173.4,283.55) | 110.93 (81.22,148.29) | -3.41 (-3.61 - -3.21) | 6537.6 (5819.35,7396) | 2774.46 (2431.78,3185.7) | -4.25 (-4.57 - -3.93) | 1.11 (0.94,1.28) | 0.51 (0.41,0.62) | -3.27 (-3.74 - -2.80) |
| High SDI | 69.67 (54.09,88.6) | 36.41 (25.47,50.86) | -2.58 (-2.98 - -2.17) | 1228.19 (1052.2,1416.27) | 631.55 (508.69,755.22) | -2.84 (-3.16 - -2.52) | 0.08 (0.08,0.09) | 0.04 (0.04,0.04) | 0(0-0) |
| High-middle SDI | 215.5 (169.74,270.81) | 61.19 (41.4,86.06) | -6.22 (-7.09 - -5.35) | 5717.75 (5019.08,6509.05) | 1253.71 (1026.63,1495.98) | -7.23 (-7.71 - -6.74) | 0.31 (0.27,0.35) | 0.09 (0.08,0.1) | -5.63 (-6.12 - -5.14) |
| Middle SDI | 219.38 (172.77,278.54) | 84.27 (59.97,114.6) | -4.55 (-4.70 - -4.39) | 6186.93 (5439.95,7036.34) | 1799.12 (1513.61,2109.38) | -5.68 (-5.93 - -5.42) | 0.79 (0.71,0.89) | 0.33 (0.28,0.4) | -3.99 (-4.22 - -3.76) |
| Low-middle SDI | 211.07 (165.19,270.1) | 112.56 (81.75,151.74) | -3.09 (-3.53 - -2.65) | 6303.09 (5610.13,7129.44) | 2717.93 (2351.99,3129.38) | -4.12 (-4.42 - -3.82) | 2.01 (1.64,2.37) | 0.81 (0.64,0.98) | -3.89 (-4.44 - -3.33) |
| Low SDI | 349.72 (268.52,454.16) | 198.27 (150.37,258.71) | -2.75 (-3.06 - -2.43) | 11748.63 (10446.34,13181.5) | 5734.12 (5060.7,6449.79) | -3.73 (-4.40 - -3.05) | 1.44 (1.1,1.8) | 0.79 (0.58,1.02) | -2.62 (-3.05 - -2.19) |
| Andean Latin America | 88.03 (68.68,112.08) | 40.13 (28.03,55.86) | -3.72 (-4.04 - -3.40) | 1525.7 (1298.53,1793.54) | 694.1 (563.84,839.13) | -4.16 (-4.95 - -3.35) | 1.14 (0.92,1.4) | 0.43 (0.33,0.57) | -3.96 (-4.49 - -3.42) |
| Australasia | 149.33 (111.57,190.09) | 61.94 (40.42,88.22) | -4.35 (-4.84 - -3.86) | 2705.04 (2247.42,3163.25) | 1037.32 (812.74,1293.8) | -4.76 (-5.22 - -4.29) | 0.03 (0.03,0.03) | 0.02 (0.01,0.02) | 0(0-0) |
| Caribbean | 109.05 (78,145.84) | 77.66 (51.54,106.95) | -1.79 (-2.17 - -1.41) | 2394.13 (1993.32,2835.71) | 1619.83 (1321.34,1938.13) | -1.86 (-2.47 - -1.25) | 1.05 (0.73,1.4) | 0.8 (0.46,1.16) | -0.94 (-1.63 - -0.26) |
| Central Asia | 351.7 (261.67,461.79) | 246.7 (162.81,348.74) | -1.74 (-1.85 - -1.63) | 8394.59 (6973.96,9700.23) | 4705.35 (3775.34,5737.35) | -3.26 (-4.25 - -2.26) | 1.86 (1.68,2.04) | 0.75 (0.63,0.91) | -3.88 (-4.32 - -3.43) |
| Central Europe | 78.43 (60.53,99.86) | 44.05 (30.43,59.97) | -2.95 (-3.33 - -2.57) | 1416.15 (1181.6,1666.42) | 813.77 (668.06,961.33) | -2.70 (-3.00 - -2.39) | 0.25 (0.23,0.27) | 0.07 (0.07,0.08) | -5.33 (-5.76 - -4.90) |
| Central Latin America | 108 (78.42,144.49) | 67.07 (44.85,94.4) | -2.28 (-2.49 - -2.07) | 2361.36 (1984.75,2803.47) | 1169.62 (939.55,1417.76) | -3.39 (-3.65 - -3.13) | 0.51 (0.46,0.57) | 0.21 (0.18,0.25) | -3.73 (-4.04 - -3.42) |
| Central Sub-Saharan Africa | 543.07 (396.5,723.01) | 300.61 (226.93,391.29) | -2.87 (-3.17 - -2.58) | 23535.33 (20826.7,26741.04) | 11027.33 (9719.63,12379.62) | -4.39 (-5.81 - -2.94) | 1.45 (0.92,2.27) | 0.7 (0.38,1.34) | -3.42 (-3.69 - -3.16) |
| East Asia | 332.36 (258.12,426.39) | 58.13 (37.57,85.78) | -8.65 (-9.19 - -8.10) | 10062.43 (8794.62,11490.45) | 1358.32 (1106.09,1641.48) | -9.60 (-10.05 - -9.15) | 0.27 (0.23,0.33) | 0.06 (0.04,0.07) | -6.97 (-7.41 - -6.53) |
| Eastern Europe | 149.28 (107.63,197.42) | 105.33 (68.87,147.9) | -1.75 (-1.97 - -1.53) | 3279.47 (2823.63,3789.35) | 1662.92 (1313.99,2064.87) | -3.78 (-4.81 - -2.74) | 0.24 (0.23,0.25) | 0.09 (0.09,0.1) | -4.95 (-5.94 - -3.96) |
| Eastern Sub-Saharan Africa | 334.45 (253.3,438.87) | 152.02 (113.13,202.33) | -3.86 (-4.39 - -3.32) | 10217.53 (8991.51,11559.38) | 3931.8 (3437.14,4483.08) | -5.22 (-6.73 - -3.68) | 0.64 (0.48,0.83) | 0.37 (0.24,0.51) | -2.41 (-2.59 - -2.23) |
| High-income Asia Pacific | 93.58 (77.83,112.76) | 26 (18.75,35.46) | -1.53 (-2.53 - -0.52) | 1275.52 (1091.55,1472.45) | 390.76 (312.43,475.23) | -1.61 (-2.70 - -0.51) | 0.1 (0.08,0.11) | 0.04 (0.03,0.04) | -4.06 (-5.25 - -2.84) |
| High-income North America | 41.13 (28.6,57.35) | 31.95 (21.43,45.47) | -1.20 (-1.27 - -1.13) | 642.87 (521.93,768.05) | 492.41 (393.8,596.71) | -1.23 (-1.38 - -1.08) | 0.05 (0.05,0.06) | 0.04 (0.04,0.04) | 0(0-0) |
| North Africa and Middle East | 124.25 (97.77,157.39) | 69.07 (49.49,94.58) | -3.04 (-3.37 - -2.71) | 3953.12 (3532.36,4426.23) | 1856.37 (1578.29,2164.41) | -3.92 (-4.47 - -3.36) | 1.38 (1.12,1.74) | 0.64 (0.51,0.86) | -3.45 (-3.67 - -3.23) |
| Oceania | 277.36 (202.82,370.16) | 237.47 (175.7,312.38) | -0.83 (-1.34 - -0.31) | 9722.27 (8588.36,10867.89) | 8250.19 (7314.41,9278.05) | -0.71 (-0.92 - -0.50) | 0.74 (0.54,1.13) | 0.39 (0.23,0.58) | -3.07 (-3.42 - -2.73) |
| South Asia | 152.13 (118.42,194.34) | 70.5 (50.45,95.76) | -3.81 (-4.20 - -3.42) | 4838.61 (4297.41,5462.07) | 1708.39 (1460.95,1975.28) | -5.15 (-5.67 - -4.63) | 2.12 (1.68,2.56) | 0.77 (0.61,0.96) | -4.86 (-5.85 - -3.85) |
| Southeast Asia | 258.69 (199.74,329.49) | 139.52 (100.24,188.44) | -2.80 (-3.74 - -1.86) | 6211.63 (5431.59,7065.15) | 2893.65 (2449.12,3384.75) | -3.66 (-4.25 - -3.07) | 1.27 (0.99,1.58) | 0.59 (0.49,0.71) | -3.50 (-3.66 - -3.35) |
| Southern Latin America | 42.87 (33.07,54.77) | 20.29 (13.48,29.23) | -3.75 (-4.14 - -3.35) | 651.91 (520.39,834.68) | 321.2 (248.67,400.77) | -3.72 (-4.07 - -3.36) | 0.21 (0.2,0.24) | 0.1 (0.08,0.11) | -3.16 (-3.67 - -2.64) |
| Southern Sub-Saharan Africa | 227.6 (174.53,287.79) | 99.18 (72.82,133.93) | -3.94 (-4.33 - -3.55) | 4809.15 (4055.77,5615.25) | 1802.7 (1518.67,2133.53) | -4.45 (-4.87 - -4.04) | 0.52 (0.43,0.61) | 0.41 (0.3,0.55) | -1.51 (-2.73 - -0.28) |
| Tropical Latin America | 147.34 (110.44,191.81) | 78.45 (51.07,113.51) | -3.09 (-3.63 - -2.55) | 3026.56 (2517.16,3619.75) | 1290.56 (1009.19,1595.86) | -4.40 (-4.89 - -3.91) | 0.4 (0.34,0.45) | 0.13 (0.1,0.16) | -5.09 (-5.65 - -4.53) |
| Western Europe | 54.55 (40.98,70.79) | 36.6 (25.87,49.66) | -1.94 (-2.20 - -1.68) | 836.25 (687.85,1006.44) | 543.42 (440.15,659.93) | -2.10 (-2.23 - -1.98) | 0.06 (0.05,0.06) | 0.03 (0.02,0.03) | 0(0-0) |
| Western Sub-Saharan Africa | 544.26 (425.56,700.88) | 287.17 (218.1,377.27) | -3.03 (-3.19 - -2.87) | 17140.16 (15234.36,19169.41) | 7769.29 (6881.81,8810.09) | -4.08 (-5.02 - -3.14) | 1.55 (1.2,1.98) | 1.09 (0.71,1.57) | -1.70 (-2.07 - -1.34) |
| **10-14years** |  | | | | | | | | |
| Global | 169.49 (133.12,207.26) | 77.83 (58.31,101.7) | -3.80 (-4.00 - -3.59) | 7319.39 (6551.03,8219.59) | 3280.55 (2874.33,3726.19) | -3.86 (-4.04 - -3.68) | 0.98 (0.81,1.17) | 0.65 (0.57,0.75) | -2.12 (-2.39 - -1.85) |
| High SDI | 60.18 (47.49,74.96) | 31.35 (22.58,43.03) | -3.17 (-3.29 - -3.04) | 1734.08 (1511.56,1988.65) | 887.25 (742.96,1039.14) | -3.23 (-3.37 - -3.08) | 1.59 (1.26,2.06) | 0.05 (0.04,0.05) | 0(0-0) |
| High-middle SDI | 172.15 (137.21,211.72) | 37.98 (25.06,55.6) | -7.95 (-10.27 - -5.56) | 6791.12 (6049.28,7667.97) | 1509.76 (1246.26,1792.47) | -7.93 (-9.92 - -5.90) | 1.13 (0.88,1.44) | 0.14 (0.12,0.15) | -4.02 (-4.32 - -3.72) |
| Middle SDI | 184.3 (145.14,225.33) | 61.91 (45.1,82.77) | -5.26 (-5.79 - -4.73) | 7697.87 (6847.34,8693.39) | 2480.89 (2136.21,2859.66) | -5.46 (-6.04 - -4.87) | 0.91 (0.7,1.16) | 0.44 (0.38,0.5) | -2.87 (-3.09 - -2.64) |
| Low-middle SDI | 153.13 (120.43,190.57) | 79.6 (59.65,105.77) | -3.17 (-3.44 - -2.91) | 6890.6 (6177.07,7716.47) | 3392.36 (2952.53,3854.83) | -3.41 (-3.66 - -3.16) | 0.26 (0.23,0.3) | 0.99 (0.85,1.17) | -2.72 (-3.06 - -2.39) |
| Low SDI | 245.55 (188.11,306.14) | 139.12 (107.32,177.55) | -2.86 (-3.06 - -2.67) | 12182.79 (10901.48,13673.03) | 6223.3 (5550.95,7005.77) | -3.29 (-3.53 - -3.05) | 0.45 (0.42,0.48) | 1 (0.8,1.24) | -2.16 (-2.48 - -1.83) |
| Andean Latin America | 65.89 (52.31,81.7) | 27.15 (18.73,39.74) | -4.52 (-4.90 - -4.13) | 1829.68 (1574.42,2126.42) | 840.83 (685.79,1014.82) | -3.90 (-4.28 - -3.51) | 0.23 (0.2,0.26) | 0.47 (0.36,0.61) | -3.07 (-3.34 - -2.80) |
| Australasia | 100.52 (76.39,130.19) | 36.58 (23.62,55.32) | -5.58 (-7.51 - -3.61) | 3270.97 (2791.31,3756.56) | 1231.82 (968.21,1535.94) | -5.35 (-7.08 - -3.59) | 2.46 (2.27,2.68) | 0.02 (0.02,0.03) | -1.23 (-2.52 - 0.08) |
| Caribbean | 72.7 (52.15,99.62) | 58.16 (40.88,81.24) | -1.10 (-1.36 - -0.83) | 2818.05 (2385.47,3315.22) | 2225.48 (1848.37,2642.56) | -1.12 (-1.27 - -0.97) | 0.09 (0.08,0.11) | 0.79 (0.55,1.04) | -0.71 (-1.00 - -0.42) |
| Central Asia | 213.74 (162.1,280.55) | 118.59 (71.65,183.82) | -3.30 (-3.96 - -2.65) | 9411.76 (8031.25,10759.01) | 5384.08 (4355.27,6490.87) | -3.14 (-3.84 - -2.44) | 1.05 (0.91,1.19) | 0.92 (0.8,1.08) | -4.58 (-5.24 - -3.92) |
| Central Europe | 82.03 (65.61,100.79) | 26.79 (18,39.15) | -5.40 (-5.81 - -4.99) | 2680.05 (2332.49,3050.63) | 898.59 (726.12,1083.91) | -5.32 (-5.79 - -4.84) | 0.41 (0.37,0.45) | 0.11 (0.1,0.11) | -4.15 (-4.61 - -3.68) |
| Central Latin America | 84.52 (59.86,115.61) | 42.88 (26.92,66.35) | -3.20 (-3.59 - -2.81) | 3278.17 (2787.72,3859.42) | 1332.08 (1064.7,1621.46) | -4.39 (-4.74 - -4.04) | 1.46 (0.96,2.19) | 0.24 (0.21,0.28) | -2.64 (-2.88 - -2.41) |
| Central Sub-Saharan Africa | 385.19 (267.73,500.64) | 188.18 (142.12,240.47) | -3.73 (-4.36 - -3.10) | 24041.16 (21555.45,27170.7) | 9933.25 (8813.85,11122.1) | -4.33 (-4.95 - -3.71) | 0.79 (0.57,1.23) | 0.96 (0.6,1.55) | -2.03 (-2.15 - -1.90) |
| East Asia | 249 (198.25,305.61) | 33.66 (23.64,48.37) | -10.60 (-12.58 - -8.58) | 10733.35 (9479.44,12184.04) | 1720.24 (1428.51,2045.48) | -9.70 (-11.78 - -7.57) | 0.06 (0.05,0.06) | 0.07 (0.06,0.09) | -6.11 (-6.62 - -5.59) |
| Eastern Europe | 100.06 (72.15,133.93) | 67.78 (41.08,105.13) | -2.63 (-3.42 - -1.82) | 3742.2 (3229.23,4302.35) | 2088.49 (1662.67,2596.9) | -3.73 (-4.71 - -2.73) | 0.03 (0.02,0.03) | 0.15 (0.14,0.16) | -3.29 (-4.01 - -2.56) |
| Eastern Sub-Saharan Africa | 240.07 (181.18,301.94) | 108.1 (81.43,140.67) | -4.08 (-4.71 - -3.45) | 10828.9 (9608.18,12233.59) | 4482.03 (3931.09,5110.33) | -4.60 (-5.37 - -3.82) | 0.06 (0.06,0.06) | 0.66 (0.49,0.84) | -2.18 (-2.32 - -2.04) |
| High-income Asia Pacific | 86.9 (74.14,100.89) | 64.41 (52.54,77.94) | -1.49 (-1.74 - -1.23) | 1827.51 (1601.05,2092.47) | 1395.85 (1203.65,1623.65) | -1.36 (-1.69 - -1.03) | 1.84 (1.57,2.15) | 0.04 (0.04,0.05) | -3.72 (-4.65 - -2.78) |
| High-income North America | 41.79 (30.33,56.46) | 22.88 (14.18,34.9) | -2.95 (-3.18 - -2.72) | 1080.53 (906.13,1267.74) | 618.46 (496.99,764.28) | -2.78 (-3.02 - -2.54) | 0.32 (0.31,0.34) | 0.04 (0.04,0.05) | 0(0-0) |
| North Africa and Middle East | 123.41 (100.05,149.25) | 48.41 (34.79,68.67) | -4.77 (-5.15 - -4.38) | 5109.1 (4613.76,5699.65) | 2049.66 (1755.63,2378.26) | -4.72 (-4.93 - -4.52) | 0.67 (0.56,0.81) | 0.62 (0.49,0.81) | -2.74 (-2.85 - -2.63) |
| Oceania | 239.34 (178.53,303.05) | 140.01 (103.55,178.41) | -2.78 (-3.17 - -2.38) | 12377.54 (11117,13789.13) | 7404.66 (6635.49,8309.77) | -2.65 (-3.13 - -2.18) | 1.96 (1.62,2.29) | 0.51 (0.37,0.69) | -2.01 (-2.34 - -1.68) |
| South Asia | 106.46 (83.27,132.8) | 60.55 (46.13,78.54) | -2.10 (-2.52 - -1.69) | 5093.03 (4556.13,5712.78) | 2734.78 (2401.25,3086.04) | -2.32 (-2.81 - -1.82) | 0.09 (0.09,0.1) | 1.03 (0.85,1.27) | -3.06 (-3.64 - -2.48) |
| Southeast Asia | 228.55 (179.14,282.32) | 106.76 (79.48,141.21) | -3.51 (-4.22 - -2.80) | 8765.35 (7789.38,9875.89) | 3988.09 (3430.05,4573.12) | -3.65 (-4.33 - -2.96) | 0.34 (0.32,0.37) | 0.81 (0.68,0.96) | -3.14 (-3.35 - -2.93) |
| Southern Latin America | 32.72 (25.78,41.16) | 14.1 (8.98,21.46) | -4.81 (-5.49 - -4.13) | 801.06 (647.44,987.12) | 391.83 (306.14,490.02) | -4.00 (-4.81 - -3.18) | 1.67 (1.37,2.08) | 0.13 (0.11,0.15) | -2.47 (-3.13 - -1.81) |
| Southern Sub-Saharan Africa | 212.84 (166.31,262.2) | 77.53 (58.16,102.72) | -4.82 (-5.18 - -4.47) | 6539.49 (5658.72,7532.74) | 2386.62 (2041.86,2785.98) | -5.03 (-5.18 - -4.87) | 1.59 (1.28,1.91) | 0.57 (0.41,0.73) | -1.19 (-2.89 - 0.53) |
| Tropical Latin America | 109.74 (83.68,141.53) | 47.17 (27.82,75.92) | -4.80 (-5.82 - -3.76) | 3636.77 (3092.66,4290.45) | 1407.38 (1077.64,1798.98) | -5.92 (-6.70 - -5.13) | 0.81 (0.73,0.9) | 0.18 (0.16,0.2) | -3.70 (-4.23 - -3.18) |
| Western Europe | 50.02 (38.32,64.22) | 26.79 (18.83,37.51) | -3.09 (-3.25 - -2.94) | 1306.78 (1098.99,1553.72) | 705.43 (578.93,851.15) | -3.04 (-3.19 - -2.89) | 1.07 (0.83,1.33) | 0.03 (0.03,0.03) | 0(0-0) |
| Western Sub-Saharan Africa | 382.19 (299.87,481.08) | 205.95 (159.11,263.65) | -3.38 (-3.80 - -2.96) | 17993.1 (16085.79,20069.22) | 8745.25 (7785.54,9884.87) | -3.74 (-4.09 - -3.38) | 0.33 (0.3,0.37) | 1.15 (0.82,1.54) | -1.62 (-1.81 - -1.43) |
| **15-19years** |  | | | | | | | | |
| Global | 1076.95 (867.47,1311.49) | 1176.6 (926.16,1460.32) | 0.44 (0.37 - 0.50) | 5337.56 (4418.26,6320.32) | 9533.98 (8205.08,10994.34) | -1.19 (-1.28 - -1.09) | 1.66 (1.37,1.97) | 1.78 (1.59,2) | -1.55 (-1.78 - -1.31) |
| High SDI | 920.75 (737.22,1126.48) | 1036.68 (810.66,1289.21) | 0.58 (0.56 - 0.61) | 7622.54 (6472.94,8849.24) | 6236.22 (5014.7,7604.49) | -0.26 (-0.34 - -0.19) | 4.43 (3.42,5.51) | 0.14 (0.13,0.16) | -2.86 (-2.99 - -2.74) |
| High-middle SDI | 1123.61 (905.2,1369.4) | 1273.15 (997.25,1591.38) | 0.71 (0.42 - 1.01) | 6792.34 (5635.38,8151.75) | 7568.83 (6179.57,9145.26) | -2.46 (-2.82 - -2.09) | 1.53 (1.25,1.93) | 0.41 (0.37,0.45) | -3.17 (-3.56 - -2.77) |
| Middle SDI | 1160.95 (931.69,1416.5) | 1266.18 (993.19,1575.49) | 0.44 (0.30 - 0.58) | 8572.3 (7329.79,9893.36) | 8870.47 (7521.54,10378.36) | -1.85 (-1.98 - -1.72) | 1.24 (1,1.5) | 1.28 (1.15,1.44) | -2.10 (-2.52 - -1.67) |
| Low-middle SDI | 1043.37 (837.77,1289.93) | 1169.16 (918.26,1463.67) | 0.56 (0.54 - 0.58) | 14590.27 (12437.04,16966.05) | 9588.57 (8223.86,11074.55) | -1.06 (-1.16 - -0.97) | 0.63 (0.56,0.69) | 2.55 (2.15,2.98) | -2.33 (-2.67 - -1.98) |
| Low SDI | 1000.68 (805.6,1211.98) | 1066.91 (846.55,1313.18) | 0.30 (0.27 - 0.33) | 9233.38 (8169.84,10464.77) | 13181.13 (11833.91,14713.31) | -1.19 (-1.30 - -1.07) | 0.85 (0.82,0.9) | 2.96 (2.42,3.48) | -1.99 (-2.27 - -1.71) |
| Andean Latin America | 1091.06 (836.84,1365.08) | 1281.69 (988.4,1611.78) | 0.78 (0.73 - 0.84) | 12102.16 (10832.36,13461.27) | 6429.88 (5143.64,7930.6) | -0.29 (-0.35 - -0.23) | 0.42 (0.38,0.46) | 0.86 (0.64,1.14) | -3.17 (-3.40 - -2.95) |
| Australasia | 796.23 (623.98,973.98) | 859.13 (670.54,1075.47) | 0.32 (0.24 - 0.40) | 6449.36 (5493.08,7612.5) | 5351.22 (4309.53,6524.09) | -1.74 (-2.28 - -1.20) | 5.51 (5.06,5.96) | 0.06 (0.05,0.07) | -1.00 (-1.55 - -0.44) |
| Caribbean | 1001.5 (779.19,1251.03) | 1115.24 (864.91,1386.18) | 0.50 (0.48 - 0.53) | 15675.48 (13828.75,17778.52) | 7152.52 (5937.5,8525.22) | -0.33 (-0.42 - -0.25) | 0.24 (0.22,0.27) | 1.26 (0.88,1.74) | 0.57 (-0.01 - 1.15) |
| Central Asia | 1297.51 (1023.83,1589.62) | 1444.25 (1123.83,1791.4) | 0.55 (0.42 - 0.68) | 7213.23 (6137.44,8354.71) | 12390.65 (10433.14,14549.51) | -1.30 (-1.52 - -1.09) | 2.44 (2.21,2.75) | 1.89 (1.65,2.23) | -4.41 (-4.99 - -3.83) |
| Central Europe | 1002.97 (789.08,1247.02) | 1119.11 (863.88,1393.28) | 0.55 (0.50 - 0.60) | 13883.71 (12460.68,15394.84) | 5912.3 (4722.83,7255.41) | -1.18 (-1.35 - -1.02) | 0.85 (0.8,0.89) | 0.32 (0.29,0.35) | -2.81 (-3.58 - -2.03) |
| Central Latin America | 1267.26 (977.17,1581.63) | 1435.5 (1110.66,1802.32) | 0.57 (0.53 - 0.61) | 15582.56 (14054.05,17388.43) | 7509.46 (6031.41,9193.59) | -0.79 (-0.98 - -0.61) | 3.62 (2.61,4.85) | 0.59 (0.52,0.67) | -1.65 (-2.11 - -1.19) |
| Central Sub-Saharan Africa | 1080.95 (872.36,1301.41) | 1103.28 (884.81,1330.69) | 0.15 (0.09 - 0.20) | 19122.36 (17489.82,21004.95) | 22704.32 (20672.88,25122.5) | -0.86 (-0.98 - -0.74) | 1.35 (0.94,2.13) | 2.91 (1.96,4.35) | -1.05 (-1.15 - -0.95) |
| East Asia | 1068.88 (862.59,1291.68) | 1104.03 (860.51,1383.84) | 0.55 (0.36 - 0.74) | 8209.31 (7013.77,9448.53) | 7441.29 (6247.43,8841.79) | -3.91 (-4.33 - -3.47) | 0.17 (0.17,0.18) | 0.2 (0.16,0.24) | -5.46 (-5.77 - -5.15) |
| Eastern Europe | 948.12 (736.56,1180.2) | 1069.43 (831.03,1344.66) | 0.58 (0.56 - 0.59) | 4146.7 (3333.25,5057.66) | 6842.9 (5602.57,8256.24) | -1.06 (-1.27 - -0.86) | 0.09 (0.08,0.1) | 0.36 (0.33,0.38) | -4.28 (-5.14 - -3.41) |
| Eastern Sub-Saharan Africa | 992.03 (796.05,1206.89) | 1009.84 (794.13,1247.35) | 0.05 (-0.02 - 0.13) | 7117.52 (6144.22,8181.95) | 10217.79 (8952.48,11603.89) | -2.16 (-2.44 - -1.89) | 0.17 (0.16,0.18) | 2.66 (2.06,3.26) | -1.82 (-1.85 - -1.78) |
| High-income Asia Pacific | 884 (733.13,1057.66) | 934.95 (736.8,1146.37) | 0.20 (0.11 - 0.29) | 8680.17 (7318.02,10148.91) | 6314.04 (5212.4,7509.88) | -0.10 (-0.14 - -0.06) | 4.06 (3.55,4.76) | 0.12 (0.1,0.14) | -3.24 (-4.05 - -2.43) |
| High-income North America | 811.37 (640.79,1000.97) | 891.96 (696.22,1108.4) | 0.48 (0.42 - 0.54) | 5055.93 (4147.02,6113.75) | 5749.03 (4534.6,7139.29) | 0.52 (0.31 - 0.74) | 0.91 (0.88,0.94) | 0.13 (0.12,0.14) | -1.43 (-2.00 - -0.85) |
| North Africa and Middle East | 2070.97 (1635.57,2564.78) | 2392.31 (1873.08,2991.06) | 0.67 (0.62 - 0.73) | 11703.26 (10462.53,13127.9) | 13545.98 (10935.43,16444.65) | -0.36 (-0.47 - -0.26) | 1.52 (1.25,1.81) | 1.03 (0.81,1.31) | -1.80 (-1.95 - -1.65) |
| Oceania | 1301.47 (1033.82,1571.71) | 1214.08 (958.9,1499.4) | -0.36 (-0.39 - -0.33) | 11910.34 (10521.64,13443.85) | 11367.24 (10044.05,12847.78) | -2.67 (-2.87 - -2.47) | 4.71 (4.1,5.51) | 0.93 (0.67,1.3) | -1.56 (-1.91 - -1.21) |
| South Asia | 792.27 (621.55,977.13) | 918.38 (713.04,1142.78) | 0.73 (0.69 - 0.76) | 12731.32 (11346.54,14208.6) | 8362.95 (7228.52,9612.57) | -0.48 (-0.51 - -0.45) | 0.26 (0.24,0.27) | 2.72 (2.04,3.39) | -2.69 (-3.24 - -2.14) |
| Southeast Asia | 1143.51 (920.55,1394.51) | 1178.48 (926.66,1458.73) | 0.17 (0.13 - 0.21) | 15247.96 (13753.02,16815.11) | 9418.04 (8097.45,10866.51) | -1.85 (-2.14 - -1.56) | 0.59 (0.56,0.62) | 2.45 (1.96,2.95) | -1.88 (-1.97 - -1.78) |
| Southern Latin America | 674.95 (529.19,839.47) | 748.32 (579.17,932.32) | 0.53 (0.49 - 0.58) | 11862.4 (10582.84,13295.4) | 4154.96 (3283.95,5163.67) | 0.02 (-0.05 - 0.10) | 4.53 (3.88,5.19) | 0.32 (0.28,0.35) | -1.18 (-1.82 - -0.55) |
| Southern Sub-Saharan Africa | 1260.49 (976.97,1556.65) | 1329.56 (1011.2,1659.83) | 0.29 (0.24 - 0.35) | 6574.66 (5598.66,7677.18) | 8452.21 (7062.57,9983.11) | -1.71 (-1.92 - -1.49) | 3.63 (2.87,4.49) | 1.24 (0.94,1.62) | -1.02 (-1.29 - -0.74) |
| Tropical Latin America | 1080.15 (845.79,1337.62) | 1219.03 (936.68,1532.32) | 0.57 (0.53 - 0.61) | 16701.62 (15132.92,18455.95) | 6641.32 (5351.24,8094.83) | -1.44 (-1.73 - -1.14) | 2 (1.82,2.21) | 0.49 (0.45,0.52) | -2.29 (-2.48 - -2.11) |
| Western Europe | 852.67 (674.31,1049.71) | 966.61 (757.17,1188.67) | 0.61 (0.57 - 0.65) | 27848.18 (25305.05,31077.18) | 5361.65 (4310.97,6535.56) | -0.13 (-0.20 - -0.06) | 3.93 (3.31,4.55) | 0.09 (0.08,0.09) | 0(0-0) |
| Western Sub-Saharan Africa | 1239.77 (983.3,1490.5) | 1251.54 (991.09,1530.22) | 0.02 (-0.00 - 0.05) | 23138.49 (20996.43,25573.43) | 15790.04 (14216.95,17574.6) | -1.82 (-1.86 - -1.77) | 0.83 (0.78,0.9) | 3.36 (2.22,4.5) | -1.34 (-1.50 - -1.19) |
| **20-24years** |  |  |  |  |  |  |  |  |  |
| Global | 1139.98 (900.81,1408.18) | 1284.89 (1009.04,1598.04) | 0.67 (0.52 - 0.82) | 8909.2 (7133.19,10710.48) | 15747.02 (13045.42,18519.36) | -0.30 (-0.42 - -0.18) | 3.67 (3.41,4.03) | 2.81 (2.52,3.17) | -1.36 (-1.55 - -1.17) |
| High SDI | 813.96 (637,995.19) | 940.13 (729.18,1169.68) | 0.78 (0.65 - 0.92) | 12437.83 (10180.41,14812.62) | 10045.67 (7791.8,12274.74) | 0.05 (-0.04 - 0.14) | 0.48 (0.46,0.5) | 0.35 (0.32,0.38) | -1.25 (-1.41 - -1.09) |
| High-middle SDI | 1152.06 (908.37,1404.46) | 1320.81 (1023.7,1640.62) | 0.93 (0.55 - 1.31) | 10986.3 (8756.72,13418.47) | 13623.18 (10827.4,16474.09) | -0.85 (-1.41 - -0.28) | 1.53 (1.44,1.63) | 0.78 (0.7,0.86) | -3.46 (-4.01 - -2.90) |
| Middle SDI | 1250.47 (982.69,1529.43) | 1399.84 (1087.15,1731.79) | 0.63 (0.49 - 0.77) | 13969.03 (11399.66,16749.58) | 15512.79 (12759.41,18439.81) | -0.67 (-0.87 - -0.47) | 3.24 (3,3.52) | 2.19 (1.95,2.46) | -1.96 (-2.17 - -1.74) |
| Low-middle SDI | 1159.51 (910.74,1426.44) | 1316.64 (1022.3,1614.54) | 0.65 (0.61 - 0.69) | 23018.09 (18660.48,27621.12) | 16396.71 (13627.59,19282.88) | -0.18 (-0.28 - -0.07) | 6.36 (5.71,7.2) | 4.18 (3.57,4.93) | -2.25 (-2.61 - -1.89) |
| Low SDI | 1120.64 (885.3,1365.82) | 1226.5 (965.09,1500.51) | 0.46 (0.44 - 0.48) | 13985.26 (11748.73,16352.21) | 20164.49 (17550.68,23095.08) | -0.28 (-0.31 - -0.25) | 6.15 (5.37,7.19) | 4.61 (3.88,5.33) | -1.42 (-1.60 - -1.23) |
| Andean Latin America | 1110.38 (863.56,1384.56) | 1305.14 (989.95,1632.24) | 0.81 (0.78 - 0.83) | 16768.84 (14367.32,19343.08) | 11630.01 (9062.71,14271.5) | 0.26 (0.22 - 0.30) | 2.31 (1.94,2.76) | 1.24 (0.94,1.66) | -2.97 (-3.28 - -2.66) |
| Australasia | 734.89 (564.33,910.98) | 793.29 (597.82,977.35) | 0.29 (0.22 - 0.36) | 9498.44 (8112.84,11126.59) | 8615.12 (6780.02,10589.39) | -0.96 (-1.18 - -0.75) | 0.18 (0.16,0.2) | 0.13 (0.12,0.15) | -1.30 (-1.62 - -0.98) |
| Caribbean | 1096.55 (850.43,1364.35) | 1227.76 (947.24,1525.49) | 0.54 (0.52 - 0.56) | 20406.05 (17656.85,23559.65) | 12335.11 (9942.64,14853.88) | -0.03 (-0.08 - 0.03) | 1.97 (1.65,2.34) | 1.9 (1.33,2.52) | 0.35 (-0.47 - 1.17) |
| Central Asia | 1246.19 (989.88,1539.91) | 1437.12 (1139.7,1768.17) | 0.77 (0.59 - 0.95) | 10906.99 (8889.26,12962.11) | 20568.17 (17428.37,24079.19) | 0.02 (-0.04 - 0.07) | 8.7 (8.04,9.38) | 3.73 (3.3,4.23) | -4.21 (-4.66 - -3.77) |
| Central Europe | 953.8 (744.29,1167.79) | 1057.02 (821.42,1310.04) | 0.50 (0.44 - 0.56) | 18456.48 (16016.13,21256.79) | 10070.16 (7784.95,12411.42) | -0.42 (-0.53 - -0.31) | 0.96 (0.9,1.01) | 0.63 (0.57,0.68) | -1.93 (-2.54 - -1.31) |
| Central Latin America | 1304.62 (1011.71,1618.6) | 1477.77 (1141.94,1823.36) | 0.58 (0.56 - 0.60) | 20146.21 (17719.61,23037.89) | 13212.89 (10332.53,16158.42) | -0.14 (-0.16 - -0.12) | 1.89 (1.83,1.98) | 1.43 (1.29,1.56) | -0.87 (-1.27 - -0.46) |
| Central Sub-Saharan Africa | 1147.5 (904.31,1384.73) | 1196.08 (959.94,1438.62) | 0.19 (0.17 - 0.21) | 23144.71 (20673.5,26103.32) | 28044.09 (25451.98,31028.54) | -0.57 (-0.59 - -0.56) | 6.15 (4.58,7.84) | 5.41 (3.75,7.55) | -0.57 (-0.66 - -0.48) |
| East Asia | 1153.91 (912.4,1406.22) | 1259.1 (965.01,1567.53) | 0.91 (0.12 - 1.71) | 11501.81 (9595.56,13534.95) | 14149.27 (11597.77,17049.78) | -1.56 (-3.02 - -0.08) | 1.14 (1.02,1.28) | 0.48 (0.37,0.6) | -4.24 (-4.82 - -3.66) |
| Eastern Europe | 906.13 (708.1,1120.91) | 1019.56 (803.32,1267.39) | 0.58 (0.55 - 0.61) | 7632.25 (5867.24,9537.01) | 11187.67 (9170.38,13415.88) | -0.11 (-0.20 - -0.02) | 2.5 (2.41,2.63) | 1.12 (0.98,1.25) | -3.91 (-4.78 - -3.04) |
| Eastern Sub-Saharan Africa | 1097.78 (866.95,1343.77) | 1170.86 (920.58,1451.39) | 0.35 (0.32 - 0.39) | 10207.79 (8632.69,12064.98) | 18073.44 (15608.69,20796.45) | -0.33 (-0.38 - -0.27) | 5.98 (5.02,7.11) | 4.86 (3.82,5.9) | -0.98 (-1.10 - -0.86) |
| High-income Asia Pacific | 696.62 (557.36,838.46) | 724.6 (563.1,892.83) | 0.12 (-0.00 - 0.25) | 13622.04 (10933.49,16295.62) | 8749 (7075.38,10541.37) | -0.43 (-0.50 - -0.36) | 0.36 (0.33,0.41) | 0.19 (0.17,0.23) | -3.03 (-3.56 - -2.50) |
| High-income North America | 657.21 (500.36,813.79) | 736.37 (561.5,915.94) | 0.55 (0.52 - 0.57) | 8167.39 (6509.12,9974.12) | 8677.79 (6747.11,10784.98) | 0.24 (0.18 - 0.31) | 0.4 (0.38,0.41) | 0.37 (0.36,0.39) | -0.08 (-0.36 - 0.20) |
| North Africa and Middle East | 2100.72 (1626.77,2619.77) | 2363.03 (1805.37,2980.68) | 0.53 (0.43 - 0.63) | 16248.2 (13889.03,18834.54) | 23718.93 (18493.3,29016.8) | 0.08 (-0.04 - 0.21) | 2.07 (1.74,2.55) | 1.39 (1.13,1.69) | -1.83 (-1.94 - -1.72) |
| Oceania | 1293.66 (1028.92,1574.76) | 1207.85 (933.76,1497.46) | -0.31 (-0.45 - -0.17) | 17349.4 (14801.29,20117.85) | 16845.13 (14616.79,19460.56) | -1.44 (-1.55 - -1.33) | 2.17 (1.55,3.22) | 1.61 (1.23,2.05) | -1.20 (-1.72 - -0.68) |
| South Asia | 947.24 (732.63,1175.13) | 1123.56 (868.97,1390.71) | 0.86 (0.81 - 0.92) | 17842.46 (15223.5,20660.75) | 14139.21 (11770.77,16740.97) | 0.15 (0.05 - 0.25) | 6.98 (6.21,7.95) | 4.36 (3.49,5.35) | -2.45 (-2.88 - -2.03) |
| Southeast Asia | 1217.31 (960.96,1501.22) | 1300.69 (1010.26,1611.81) | 0.34 (0.31 - 0.37) | 19764.18 (17379.24,22412.06) | 15454.88 (12927.77,18156.11) | -0.90 (-1.06 - -0.75) | 6.15 (5.08,7.17) | 4.08 (3.23,5.08) | -1.99 (-2.10 - -1.88) |
| Southern Latin America | 717.86 (547.37,896.95) | 817.43 (618.34,1027.17) | 0.65 (0.60 - 0.71) | 17033.62 (14507.08,19726.13) | 8394.06 (6422.52,10563.37) | 0.44 (0.37 - 0.51) | 0.66 (0.59,0.75) | 0.53 (0.46,0.61) | -0.05 (-0.53 - 0.43) |
| Southern Sub-Saharan Africa | 1398.94 (1098.37,1723.98) | 1528.75 (1181.93,1901.8) | 0.42 (0.40 - 0.44) | 10063.85 (8278.82,11945.47) | 15389.26 (12593.12,18290.93) | -0.65 (-0.75 - -0.55) | 3.07 (2.69,3.5) | 2.03 (1.61,2.7) | -2.55 (-3.39 - -1.71) |
| Tropical Latin America | 1222.96 (955.1,1514.02) | 1386.87 (1081.24,1722.39) | 0.55 (0.49 - 0.60) | 21499.43 (18953.9,24407.65) | 12700.86 (10001.58,15630.16) | -0.49 (-0.57 - -0.40) | 1.63 (1.56,1.69) | 0.8 (0.75,0.85) | -3.06 (-3.49 - -2.62) |
| Western Europe | 795.37 (619.64,976.57) | 890.98 (686.06,1101.62) | 0.55 (0.49 - 0.60) | 31533.68 (28453.76,35141.19) | 9128.07 (7041.26,11186.35) | 0.08 (0.03 - 0.13) | 0.35 (0.34,0.36) | 0.2 (0.18,0.21) | -2.76 (-3.32 - -2.19) |
| Western Sub-Saharan Africa | 1357.01 (1090.82,1646.9) | 1457.29 (1157.09,1782.51) | 0.36 (0.34 - 0.37) | 28490.43 (25513.06,31883.17) | 25032.6 (22040.06,28662.48) | -0.54 (-0.58 - -0.51) | 5.55 (4.14,7.16) | 4.44 (3.02,5.88) | -1.08 (-1.23 - -0.93) |

AAPC: Average Annual Percent Change; UI: uncertainty interval; CI:confidence interval.

**Supplementary Table 3. Age-standardised incidence rate (ASIR) and age-standardised prevalence rate (ASPR) of cirrhosis among children and adolescents at the national level in 2021**

| **location** | **2021** | |
| --- | --- | --- |
|  | **ASIR(95%UI)** | **ASPR(95%UI)** |
| Afghanistan | 873.27 (671.77,1093.25) | 9466.23 (7733.9,11339.76) |
| Albania | 617.83 (468.85,780.34) | 4650.58 (3639.8,5836.79) |
| Algeria | 1080.5 (827.06,1364.08) | 8879.22 (7024.9,10955.16) |
| American Samoa | 967.23 (729.64,1239.24) | 6720.35 (5345.67,8142.13) |
| Andorra | 422.31 (324.97,533.9) | 3432.65 (2639.98,4255.59) |
| Angola | 652.55 (509.66,811.53) | 16179.61 (14304.06,18123.98) |
| Antigua and Barbuda | 584.15 (443.67,744.76) | 4439.45 (3424.62,5541.34) |
| Argentina | 353.92 (267.33,449.81) | 2912.43 (2249.96,3696.75) |
| Armenia | 779.4 (587.7,998.11) | 8433.2 (6866.07,10221.73) |
| Australia | 410.01 (310.41,518.65) | 3816.69 (3008.08,4703.86) |
| Austria | 426.01 (324.33,533.16) | 3441.23 (2661.19,4274.86) |
| Azerbaijan | 849.39 (647.3,1078.93) | 10617.87 (8790.25,12638.16) |
| Bahamas | 598.17 (450.01,761.37) | 4720.25 (3699.58,5842.67) |
| Bahrain | 1290.85 (982.08,1628.98) | 9249.92 (7111.73,11590.49) |
| Bangladesh | 486.12 (372.01,609.28) | 5128.24 (4203.24,6101.84) |
| Barbados | 622.82 (468.74,792.69) | 4655.91 (3654.01,5784.04) |
| Belarus | 511.34 (387.45,652.73) | 4741.16 (3782.75,5788.57) |
| Belgium | 399.79 (305.37,507.41) | 3251.64 (2514.99,4044.2) |
| Belize | 713.72 (546.02,902.76) | 5575.18 (4393.45,6909.65) |
| Benin | 680.24 (525.7,857.77) | 10103.84 (8567.57,11845.09) |
| Bermuda | 687.51 (518.69,885.46) | 4960.39 (3897.77,6153.08) |
| Bhutan | 548.59 (414.91,708.51) | 5579.76 (4480.61,6785.44) |
| Bolivia (Plurinational State of) | 572.01 (433.66,715.55) | 4093.92 (3175.03,5110.48) |
| Bosnia and Herzegovina | 652.04 (494,814.47) | 4883.55 (3754.25,6084.74) |
| Botswana | 637.55 (487.1,807.12) | 5810.33 (4750.41,6994.21) |
| Brazil | 640.64 (491.04,810.21) | 5175.55 (4089.52,6370.71) |
| Brunei Darussalam | 513.23 (384.49,646.83) | 4494.99 (3495.58,5600.62) |
| Bulgaria | 505.65 (384.85,642.21) | 4172.67 (3303.77,5126.32) |
| Burkina Faso | 547.19 (421.03,699.56) | 10708.03 (9365.23,12192.88) |
| Burundi | 493.46 (372.08,635.46) | 6947.69 (5776.98,8299.68) |
| Cambodia | 562.45 (425.51,720.27) | 9289.14 (7858.85,10849.85) |
| Cameroon | 717.84 (550.31,910.92) | 10771.58 (9403.44,12329.69) |
| Canada | 331.36 (253.09,416.91) | 4116.81 (3431.49,4889.47) |
| Cabo Verde | 711.02 (536.48,891.6) | 8924.52 (7517.09,10442.45) |
| Central African Republic | 618.11 (480.34,767.31) | 15961.71 (14460.65,17689.05) |
| Chad | 688.43 (529.61,862.65) | 18500.38 (15122.43,21627.88) |
| Chile | 423.03 (319.22,536.52) | 3510.72 (2733.1,4399.92) |
| China | 571.02 (437.81,717.78) | 5694.7 (4694.35,6838.44) |
| Colombia | 675.45 (514.34,851.18) | 5892.04 (4761.19,7166.78) |
| Comoros | 655.54 (499.56,819.84) | 7977.29 (6684.74,9477.68) |
| Congo | 580.65 (448.5,733.12) | 12001.42 (10554.51,13502.71) |
| Cook Islands | 742.22 (558.71,956.4) | 5024.28 (3945.32,6217.36) |
| Costa Rica | 712.77 (535.46,909.43) | 5329.42 (4136.43,6566.81) |
| Cote dIvoire | 605.02 (461.59,761.95) | 10133.32 (8932.83,11469.1) |
| Croatia | 473.35 (358.95,599.19) | 3696.45 (2828.94,4627.3) |
| Cuba | 646.3 (486.09,825.45) | 4785.78 (3749.64,5989.91) |
| Cyprus | 396.78 (300.25,501.39) | 3172.37 (2408.87,3993.11) |
| Czechia | 443.52 (339.37,566.56) | 3531.02 (2779.87,4363.58) |
| Democratic Republic of the Congo | 674.51 (533.31,828.15) | 18394.57 (16483.23,20472.51) |
| Denmark | 367.93 (284.53,458.9) | 3484.09 (2762.31,4284.63) |
| Djibouti | 620.41 (477.65,777.6) | 9281.58 (7862.45,10946.97) |
| Dominica | 659.45 (497.14,833.81) | 5076.9 (4002.46,6275.35) |
| Dominican Republic | 523.95 (398.38,657.31) | 4971.94 (4016.43,6013.59) |
| Ecuador | 735.23 (560.72,942.38) | 4953.7 (3786.65,6217.67) |
| Egypt | 1543.74 (1168.51,1986.41) | 11961.29 (9456.98,14807.47) |
| El Salvador | 683.05 (512.05,874.61) | 5494.97 (4347.32,6750.68) |
| Equatorial Guinea | 657.01 (503.38,826.52) | 8430.29 (7185.66,9879.27) |
| Eritrea | 479.99 (366.62,610.12) | 6824.43 (5707.45,8083.03) |
| Estonia | 469.58 (353.94,602.64) | 4253.37 (3441.82,5206.22) |
| Ethiopia | 564.67 (437.56,701.48) | 9322.28 (8113.26,10725.86) |
| Micronesia (Federated States of) | 718.22 (548.59,911.11) | 6883.22 (5685.08,8234.6) |
| Fiji | 797.16 (606.95,1010.89) | 5864.67 (4670.37,7171.7) |
| Finland | 344.6 (267.88,429.32) | 3521.94 (2833.55,4303.6) |
| France | 395.05 (305.26,498.2) | 3289.07 (2563.73,4071.2) |
| Gabon | 659.33 (502.35,834.34) | 10543.24 (9199.71,11989.89) |
| Georgia | 733.02 (547.9,937.52) | 7395.45 (5851,9145.19) |
| Germany | 389.32 (295.16,492.57) | 3109.09 (2394.43,3930.25) |
| Ghana | 681.19 (516.57,859.15) | 8785.56 (7445.41,10300.92) |
| Greece | 399.75 (306.3,503.83) | 3122.77 (2405.39,3911.11) |
| Greenland | 367.89 (281.69,464.85) | 4305.1 (3474.21,5249.37) |
| Grenada | 591.02 (446.88,748.43) | 4585.23 (3547.43,5716.54) |
| Guam | 770.19 (591.79,980.02) | 6062.04 (4870.2,7346.69) |
| Guatemala | 691.29 (521.7,875.91) | 6508.65 (5253.46,7902.79) |
| Guinea | 751.27 (584.44,926.32) | 16106.59 (13548.38,18512.55) |
| Guinea-Bissau | 678.55 (533.81,828.57) | 13170.22 (11403.43,14917.61) |
| Guyana | 590.84 (448.19,746.27) | 4951.12 (3957.39,6085.1) |
| Haiti | 526.84 (401.94,664.38) | 6477.13 (5388.92,7708.93) |
| Honduras | 716.04 (543.11,904.92) | 6138.72 (4814.64,7569.34) |
| Hungary | 512.17 (395.79,638.54) | 4460.64 (3552.64,5477.03) |
| Iceland | 435.48 (332.46,546.55) | 3691.57 (2873.16,4600.53) |
| India | 495.53 (383.81,617.57) | 6379.7 (5451.44,7425.93) |
| Indonesia | 713.06 (549.32,892.67) | 7962.53 (6702.94,9356.5) |
| Iran (Islamic Republic of) | 1253.09 (966.53,1578.66) | 8649.74 (6628.15,10817.08) |
| Iraq | 1075.52 (816.54,1364.4) | 8679.57 (6787.4,10735.87) |
| Ireland | 453.9 (347.69,567.08) | 3671.97 (2879.61,4532.05) |
| Israel | 529.24 (404.65,664.48) | 3867.88 (2942.61,4886.36) |
| Italy | 622.76 (478.85,777.95) | 4778.79 (3754.39,5930.1) |
| Jamaica | 642.28 (483.35,812.07) | 5117.93 (4060.81,6398.79) |
| Japan | 396.95 (313.86,486.55) | 3957.14 (3271.2,4702.86) |
| Jordan | 1242.93 (952.63,1569.42) | 9461.87 (7375.35,11715.98) |
| Kazakhstan | 681.15 (518.25,866.86) | 6522.64 (5265.03,7976.23) |
| Kenya | 601.07 (460.47,758.13) | 6369.36 (5255.7,7531.17) |
| Kiribati | 804.35 (623.52,1009.5) | 9702.05 (8178.1,11440.23) |
| Kuwait | 1417.46 (1085.5,1780.59) | 9881.76 (7553.83,12385.91) |
| Kyrgyzstan | 731.84 (556.3,936.02) | 10272.83 (8538.24,12302.79) |
| Lao People's Democratic Republic | 513.12 (393.27,648.67) | 8860.28 (7657.6,10160.33) |
| Latvia | 440.78 (331.43,562.01) | 3990.03 (3165.5,4901.35) |
| Lebanon | 1124.67 (866.4,1411.11) | 8636.48 (6737.87,10778.86) |
| Lesotho | 640.52 (492.81,801.99) | 7190.3 (6056.67,8516.88) |
| Liberia | 782.37 (609.42,968.42) | 14286.86 (12258.39,16451.03) |
| Libya | 1177.65 (894.9,1486.89) | 8887.13 (6890.71,11065.15) |
| Lithuania | 463.81 (347,596.42) | 4378.07 (3528.76,5341.47) |
| Luxembourg | 408 (309,515.75) | 3225.13 (2486.99,4051.76) |
| North Macedonia | 573.02 (437.19,724.88) | 4692.75 (3651.51,5820.85) |
| Madagascar | 537.68 (414.19,674.87) | 9509.27 (8169.47,10894.9) |
| Malawi | 590.27 (455.45,747.11) | 7631.44 (6479.59,8975.12) |
| Malaysia | 768.85 (578.48,986.56) | 7038.93 (5636.03,8596.03) |
| Maldives | 662.73 (492.91,842.39) | 6633.58 (5406.86,8039.23) |
| Mali | 691.64 (533.06,861.76) | 11797.75 (10271.2,13589.38) |
| Malta | 422.69 (322.21,530.86) | 3414.4 (2646.74,4266.4) |
| Marshall Islands | 669.44 (512.1,851.87) | 6731.32 (5543.96,8033.96) |
| Mauritania | 910.08 (714.85,1121.07) | 14954.38 (13163.13,16964.62) |
| Mauritius | 641 (475.6,822.27) | 6617.79 (5365.06,8038.55) |
| Mexico | 727.29 (557.69,917.74) | 5027.85 (3903.73,6296.07) |
| Republic of Moldova | 540.57 (402.55,690.62) | 5641.1 (4594.04,6843.47) |
| Monaco | 454.53 (347.59,575.55) | 3411 (2593.45,4315.39) |
| Mongolia | 776.16 (563.6,1018.5) | 12454.64 (9745.44,15479.62) |
| Montenegro | 578.82 (438.83,735.17) | 4524.63 (3568.27,5663.92) |
| Morocco | 1038.54 (792.3,1307.67) | 8313.52 (6465.33,10320.64) |
| Mozambique | 507 (387.74,638.04) | 7599.87 (6596.23,8699.92) |
| Myanmar | 623.36 (479.29,789.87) | 7380.07 (6163.27,8814.2) |
| Namibia | 557.23 (436.63,694.91) | 7426.49 (6436.46,8526.68) |
| Nauru | 640.97 (487.12,822.13) | 5720.79 (4658.14,6909.32) |
| Nepal | 457.83 (348.76,574.8) | 4197.17 (3367.48,5096.34) |
| Netherlands | 390.21 (300.18,490.38) | 3147.41 (2441.03,3938.09) |
| New Zealand | 408.7 (313,509.29) | 3824.83 (3088.78,4613.89) |
| Nicaragua | 699.78 (528.64,889.43) | 5944.32 (4690.43,7315.76) |
| Niger | 723.26 (562.7,891.46) | 17058.77 (14980.34,19395.77) |
| Nigeria | 843.04 (662.64,1038.3) | 15606.44 (13741.41,17727.83) |
| Niue | 741.67 (553.17,950.29) | 5248.33 (4164.04,6538.64) |
| Democratic People's Republic of Korea | 536.79 (416.96,674.32) | 8555.7 (7335.14,9887.27) |
| Northern Mariana Islands | 848.5 (651.95,1068.72) | 6558.14 (5281.79,7968.43) |
| Norway | 375.95 (288.46,469.64) | 2990.26 (2322.42,3732.01) |
| Oman | 1188.58 (909.2,1518.68) | 8638.38 (6652.18,10842.86) |
| Pakistan | 585.14 (447.56,737.12) | 7188.3 (5966.38,8499.01) |
| Palau | 767.88 (575.93,967.67) | 6017.75 (4821.57,7360.55) |
| Palestine | 1062.4 (816.51,1341.66) | 8436.78 (6599.1,10548.27) |
| Panama | 662.51 (502.55,839.16) | 5130.39 (4047.12,6355.71) |
| Papua New Guinea | 641.93 (496.25,804.97) | 11623.92 (10285.43,13164.14) |
| Paraguay | 528.12 (400.29,671.47) | 4771.51 (3842.61,5841.86) |
| Peru | 571.52 (433.97,719.3) | 4511.75 (3620.21,5512.39) |
| Philippines | 557.99 (431.64,698.94) | 7554.74 (6478.7,8785.99) |
| Poland | 506.48 (385.62,640.13) | 3762.84 (2946.03,4680.17) |
| Portugal | 478.33 (359.11,610.47) | 4005.89 (3082.02,5019.96) |
| Puerto Rico | 737.84 (559.89,937.09) | 5177.93 (4059.05,6462.02) |
| Qatar | 1458.92 (1112.21,1855.31) | 9800.7 (7552.57,12229.72) |
| Romania | 501.5 (383.01,638.36) | 4259.97 (3325.17,5282.49) |
| Russian Federation | 532.93 (404.39,677.98) | 4940.94 (3988.66,6034.88) |
| Rwanda | 413.95 (314.72,532.21) | 5827.43 (4905.03,6828.07) |
| Saint Kitts and Nevis | 641.04 (480.83,822.02) | 4719.09 (3645.97,5915.36) |
| Saint Lucia | 549.25 (416.57,699.56) | 4545.89 (3568.4,5662.58) |
| Saint Vincent and the Grenadines | 594.35 (451.28,767.74) | 4823.19 (3832.08,6002.08) |
| Samoa | 762.39 (587.59,963.8) | 8167.66 (6814.14,9611.08) |
| San Marino | 437.69 (332.72,553.91) | 3422.71 (2645.43,4278.05) |
| Sao Tome and Principe | 715.2 (546.8,893.7) | 9860.49 (8471.29,11427.72) |
| Saudi Arabia | 1302.53 (984.74,1674.45) | 10048.48 (7788.11,12424.87) |
| Senegal | 703.9 (541.58,884.05) | 9404.98 (7979.15,10980.02) |
| Serbia | 694.69 (535.91,869.23) | 5372.88 (4217.25,6641.72) |
| Seychelles | 667.03 (498.83,854.8) | 5764.18 (4587.29,7086.61) |
| Sierra Leone | 627.67 (487.25,780.47) | 13059.97 (10707.88,15077.49) |
| Singapore | 528.84 (405.31,664.63) | 4630.32 (3604.68,5723.37) |
| Slovakia | 458.85 (346.4,583.04) | 3607.07 (2807.14,4518.03) |
| Slovenia | 443.36 (338.38,564.64) | 3471.81 (2699.61,4313.25) |
| Solomon Islands | 646.89 (491.59,819.08) | 7540.35 (6366.04,8865.74) |
| Somalia | 853.3 (675.97,1056.64) | 20259.33 (18099.53,22665.75) |
| South Africa | 740.32 (558.43,934.2) | 5576.79 (4438.58,6745.75) |
| Republic of Korea | 422.06 (321.44,532.46) | 3780.04 (2996.49,4690.97) |
| South Sudan | 707.04 (549.23,888.56) | 12984.8 (10904.66,15276.43) |
| Spain | 461.34 (351.42,575.58) | 3650.35 (2830.98,4565.07) |
| Sri Lanka | 642.62 (483.2,814.13) | 6144.56 (5001.41,7433.51) |
| Sudan | 676.45 (529.68,836.26) | 9848.78 (8409.4,11422.03) |
| Suriname | 598.86 (458.02,758.38) | 5168.49 (4148.59,6300.41) |
| Sweden | 409.66 (314.84,510) | 3286.03 (2584.36,4062.55) |
| Switzerland | 416.78 (316.57,522.32) | 3804.92 (3017.91,4664.24) |
| Syrian Arab Republic | 1115.38 (856.02,1398.28) | 9157.2 (7234.93,11310.23) |
| Taiwan (Province of China) | 636.83 (481.95,811.74) | 6845.82 (5580.22,8288.84) |
| Tajikistan | 658.53 (490.52,845.34) | 10771.66 (9019.83,12741.14) |
| United Republic of Tanzania | 611.29 (468.76,773.95) | 7909.53 (6652.33,9274.68) |
| Thailand | 586.22 (445.68,746.98) | 5655.41 (4651.66,6813.36) |
| Gambia | 593.37 (451.74,756.16) | 6312.18 (5176.08,7553.83) |
| Timor-Leste | 585.19 (450.07,733.55) | 9018.27 (7708.72,10464.06) |
| Togo | 680.66 (530.53,838.26) | 12541.51 (10964.23,14323.74) |
| Tokelau | 671 (513.45,844.18) | 5405.27 (4328.07,6661.04) |
| Tonga | 778.3 (581.93,994.31) | 6230.79 (4966.47,7571.4) |
| Trinidad and Tobago | 625.29 (471.11,796.24) | 4912.45 (3862.84,6079.94) |
| Tunisia | 1023.03 (769.35,1294.39) | 8103.96 (6323.19,10052.85) |
| Turkey | 1028.16 (787.85,1298.26) | 10890.55 (9033,12937.45) |
| Turkmenistan | 821.8 (619.65,1065.26) | 7342.05 (6218.09,8612.29) |
| Tuvalu | 692.55 (528.49,874.9) | 8011.33 (6274.15,9858.46) |
| Uganda | 500.51 (385.8,633.75) | 7750.58 (6723.67,8901.6) |
| Ukraine | 540.38 (409.58,686.06) | 6118.35 (5031.84,7343.16) |
| United Arab Emirates | 1379.41 (1052.42,1764.23) | 9275.11 (7139.54,11509.29) |
| United Kingdom | 471.83 (367.39,583.46) | 4178.61 (3366.95,5092.93) |
| United States of America | 398.93 (305.63,499.69) | 3566.23 (2778.82,4451.73) |
| Uruguay | 349.56 (263.25,445.62) | 2910.63 (2234.08,3701.06) |
| Uzbekistan | 820.18 (624.78,1041.44) | 11884 (10028.24,14024.33) |
| Vanuatu | 744.49 (573.42,946.58) | 8610.11 (7254.55,10062.87) |
| Venezuela (Bolivarian Republic of) | 650.66 (485.8,833.03) | 5317.19 (4189.54,6511.29) |
| Viet Nam | 536.72 (418.7,669.22) | 7449.05 (6426.42,8584.98) |
| United States Virgin Islands | 651.07 (489.5,829.16) | 4639.91 (3652.55,5761.44) |
| Yemen | 836.89 (652.38,1041.59) | 10721.01 (9111.27,12499.45) |
| Zambia | 558.62 (426.91,711.48) | 6817.74 (5789.36,7952.15) |
| Zimbabwe | 646.69 (509.28,807.87) | 9400.07 (8182.92,10827.29) |

**Supplementary Table 4. Proportional contributions of different etiologies to cirrhosis incidence among individuals aged 5–24 years in 2000 and 2021, stratified by global, five SDI regions, and 21 GBD regions.**

| **Characteristics** | **2000** | | | | | **2021** | | | | |
| --- | --- | --- | --- | --- | --- | --- | --- | --- | --- | --- |
|  | **Chronic hepatitis B including cirrhosis (%)** | **Chronic hepatitis C including cirrhosis (%)** | **Cirrhosis due to alcohol (%)** | **Metabolic dysfunction-associated fatty liver disease including cirrhosis (%)** | **Cirrhosis due to other causes (%)** | **Chronic hepatitis B including cirrhosis (%)** | **Chronic hepatitis C including cirrhosis (%)** | **Cirrhosis due to alcohol (%)** | **Metabolic dysfunction-associated fatty liver disease including cirrhosis (%)** | **Cirrhosis due to other causes (%)** |
| **5-9years** |  | | | | | | | | | |
| Global | 0.70 | 0.28 | 0.00 | 0.00 | 0.02 | 0.40 | 0.56 | 0.00 | 0.00 | 0.03 |
| High SDI | 0.56 | 0.42 | 0.00 | 0.00 | 0.02 | 0.26 | 0.71 | 0.00 | 0.00 | 0.03 |
| High-middle SDI | 0.76 | 0.23 | 0.00 | 0.00 | 0.01 | 0.20 | 0.78 | 0.00 | 0.00 | 0.03 |
| Middle SDI | 0.72 | 0.26 | 0.00 | 0.00 | 0.02 | 0.30 | 0.67 | 0.00 | 0.00 | 0.03 |
| Low-middle SDI | 0.64 | 0.32 | 0.00 | 0.00 | 0.03 | 0.34 | 0.61 | 0.00 | 0.00 | 0.05 |
| Low SDI | 0.71 | 0.27 | 0.00 | 0.00 | 0.02 | 0.55 | 0.42 | 0.00 | 0.00 | 0.02 |
| Andean Latin America | 0.63 | 0.35 | 0.00 | 0.00 | 0.02 | 0.26 | 0.70 | 0.00 | 0.00 | 0.04 |
| Australasia | 0.61 | 0.39 | 0.00 | 0.00 | 0.00 | 0.22 | 0.77 | 0.00 | 0.00 | 0.01 |
| Caribbean | 0.41 | 0.57 | 0.00 | 0.00 | 0.02 | 0.24 | 0.74 | 0.00 | 0.00 | 0.03 |
| Central Asia | 0.33 | 0.66 | 0.00 | 0.00 | 0.02 | 0.07 | 0.92 | 0.00 | 0.00 | 0.02 |
| Central Europe | 0.52 | 0.45 | 0.00 | 0.00 | 0.03 | 0.25 | 0.72 | 0.00 | 0.00 | 0.03 |
| Central Latin America | 0.48 | 0.51 | 0.00 | 0.00 | 0.01 | 0.18 | 0.80 | 0.00 | 0.00 | 0.02 |
| Central Sub-Saharan Africa | 0.76 | 0.23 | 0.00 | 0.00 | 0.01 | 0.63 | 0.35 | 0.00 | 0.00 | 0.01 |
| East Asia | 0.86 | 0.13 | 0.00 | 0.00 | 0.01 | 0.24 | 0.75 | 0.00 | 0.00 | 0.02 |
| Eastern Europe | 0.40 | 0.57 | 0.00 | 0.00 | 0.03 | 0.09 | 0.88 | 0.00 | 0.00 | 0.03 |
| Eastern Sub-Saharan Africa | 0.74 | 0.25 | 0.00 | 0.00 | 0.02 | 0.51 | 0.46 | 0.00 | 0.00 | 0.03 |
| High-income Asia Pacific | 0.79 | 0.19 | 0.00 | 0.00 | 0.02 | 0.32 | 0.62 | 0.00 | 0.00 | 0.06 |
| High-income North America | 0.29 | 0.70 | 0.00 | 0.00 | 0.02 | 0.17 | 0.80 | 0.00 | 0.00 | 0.03 |
| North Africa and Middle East | 0.66 | 0.32 | 0.00 | 0.00 | 0.02 | 0.35 | 0.61 | 0.00 | 0.00 | 0.04 |
| Oceania | 0.73 | 0.26 | 0.00 | 0.00 | 0.01 | 0.69 | 0.31 | 0.00 | 0.00 | 0.01 |
| South Asia | 0.67 | 0.27 | 0.00 | 0.00 | 0.05 | 0.29 | 0.61 | 0.00 | 0.00 | 0.10 |
| Southeast Asia | 0.60 | 0.38 | 0.00 | 0.00 | 0.02 | 0.32 | 0.65 | 0.00 | 0.00 | 0.02 |
| Southern Latin America | 0.62 | 0.36 | 0.00 | 0.00 | 0.03 | 0.18 | 0.77 | 0.00 | 0.00 | 0.05 |
| Southern Sub-Saharan Africa | 0.72 | 0.27 | 0.00 | 0.00 | 0.02 | 0.41 | 0.56 | 0.00 | 0.00 | 0.03 |
| Tropical Latin America | 0.58 | 0.42 | 0.00 | 0.00 | 0.00 | 0.16 | 0.83 | 0.00 | 0.00 | 0.01 |
| Western Europe | 0.51 | 0.47 | 0.00 | 0.00 | 0.02 | 0.36 | 0.62 | 0.00 | 0.00 | 0.03 |
| Western Sub-Saharan Africa | 0.68 | 0.31 | 0.00 | 0.00 | 0.01 | 0.55 | 0.43 | 0.00 | 0.00 | 0.02 |
| **10-14years** |  |  |  |  |  |  |  |  |  |  |
| Global | 0.80 | 0.19 | 0.00 | 0.00 | 0.01 | 0.54 | 0.43 | 0.00 | 0.00 | 0.03 |
| High SDI | 0.67 | 0.31 | 0.00 | 0.00 | 0.02 | 0.45 | 0.52 | 0.00 | 0.00 | 0.03 |
| High-middle SDI | 0.84 | 0.15 | 0.00 | 0.00 | 0.01 | 0.29 | 0.68 | 0.00 | 0.00 | 0.04 |
| Middle SDI | 0.83 | 0.16 | 0.00 | 0.00 | 0.01 | 0.48 | 0.49 | 0.00 | 0.00 | 0.03 |
| Low-middle SDI | 0.74 | 0.24 | 0.00 | 0.00 | 0.02 | 0.51 | 0.45 | 0.00 | 0.00 | 0.04 |
| Low SDI | 0.78 | 0.21 | 0.00 | 0.00 | 0.01 | 0.65 | 0.33 | 0.00 | 0.00 | 0.02 |
| Andean Latin America | 0.69 | 0.29 | 0.00 | 0.00 | 0.02 | 0.32 | 0.64 | 0.00 | 0.00 | 0.04 |
| Australasia | 0.70 | 0.29 | 0.00 | 0.00 | 0.01 | 0.31 | 0.68 | 0.00 | 0.00 | 0.01 |
| Caribbean | 0.51 | 0.48 | 0.00 | 0.00 | 0.01 | 0.44 | 0.55 | 0.00 | 0.00 | 0.01 |
| Central Asia | 0.49 | 0.49 | 0.00 | 0.00 | 0.02 | 0.11 | 0.87 | 0.00 | 0.00 | 0.02 |
| Central Europe | 0.73 | 0.26 | 0.00 | 0.00 | 0.02 | 0.28 | 0.68 | 0.00 | 0.00 | 0.04 |
| Central Latin America | 0.59 | 0.40 | 0.00 | 0.00 | 0.01 | 0.16 | 0.82 | 0.00 | 0.00 | 0.02 |
| Central Sub-Saharan Africa | 0.82 | 0.18 | 0.00 | 0.00 | 0.00 | 0.67 | 0.32 | 0.00 | 0.00 | 0.01 |
| East Asia | 0.93 | 0.07 | 0.00 | 0.00 | 0.00 | 0.46 | 0.51 | 0.00 | 0.00 | 0.03 |
| Eastern Europe | 0.46 | 0.51 | 0.00 | 0.00 | 0.03 | 0.11 | 0.86 | 0.00 | 0.00 | 0.04 |
| Eastern Sub-Saharan Africa | 0.79 | 0.20 | 0.00 | 0.00 | 0.01 | 0.59 | 0.39 | 0.00 | 0.00 | 0.02 |
| High-income Asia Pacific | 0.84 | 0.14 | 0.00 | 0.00 | 0.02 | 0.81 | 0.16 | 0.00 | 0.00 | 0.03 |
| High-income North America | 0.47 | 0.50 | 0.00 | 0.00 | 0.02 | 0.17 | 0.79 | 0.00 | 0.00 | 0.04 |
| North Africa and Middle East | 0.79 | 0.20 | 0.00 | 0.00 | 0.01 | 0.42 | 0.56 | 0.00 | 0.00 | 0.02 |
| Oceania | 0.86 | 0.14 | 0.00 | 0.00 | 0.01 | 0.75 | 0.24 | 0.00 | 0.00 | 0.01 |
| South Asia | 0.76 | 0.20 | 0.00 | 0.00 | 0.04 | 0.58 | 0.36 | 0.00 | 0.00 | 0.07 |
| Southeast Asia | 0.75 | 0.23 | 0.00 | 0.00 | 0.01 | 0.49 | 0.49 | 0.00 | 0.00 | 0.02 |
| Southern Latin America | 0.66 | 0.31 | 0.00 | 0.00 | 0.03 | 0.20 | 0.74 | 0.00 | 0.00 | 0.06 |
| Southern Sub-Saharan Africa | 0.80 | 0.19 | 0.00 | 0.00 | 0.01 | 0.52 | 0.46 | 0.00 | 0.00 | 0.02 |
| Tropical Latin America | 0.64 | 0.35 | 0.00 | 0.00 | 0.01 | 0.11 | 0.87 | 0.00 | 0.00 | 0.02 |
| Western Europe | 0.63 | 0.34 | 0.00 | 0.00 | 0.03 | 0.41 | 0.55 | 0.00 | 0.00 | 0.04 |
| Western Sub-Saharan Africa | 0.76 | 0.23 | 0.00 | 0.00 | 0.01 | 0.65 | 0.34 | 0.00 | 0.00 | 0.01 |
| **15-19years** |  |  |  |  |  |  |  |  |  |  |
| Global | 0.11 | 0.02 | 0.00 | 0.87 | 0.00 | 0.04 | 0.02 | 0.00 | 0.93 | 0.00 |
| High SDI | 0.05 | 0.02 | 0.00 | 0.93 | 0.00 | 0.01 | 0.01 | 0.00 | 0.97 | 0.00 |
| High-middle SDI | 0.11 | 0.02 | 0.00 | 0.87 | 0.00 | 0.01 | 0.02 | 0.00 | 0.97 | 0.00 |
| Middle SDI | 0.11 | 0.02 | 0.00 | 0.87 | 0.00 | 0.03 | 0.02 | 0.00 | 0.95 | 0.00 |
| Low-middle SDI | 0.10 | 0.03 | 0.00 | 0.87 | 0.00 | 0.04 | 0.02 | 0.00 | 0.93 | 0.00 |
| Low SDI | 0.17 | 0.04 | 0.00 | 0.79 | 0.00 | 0.11 | 0.03 | 0.00 | 0.86 | 0.00 |
| Andean Latin America | 0.04 | 0.01 | 0.00 | 0.95 | 0.00 | 0.01 | 0.01 | 0.00 | 0.98 | 0.00 |
| Australasia | 0.08 | 0.03 | 0.00 | 0.89 | 0.00 | 0.01 | 0.02 | 0.00 | 0.97 | 0.00 |
| Caribbean | 0.04 | 0.03 | 0.00 | 0.93 | 0.00 | 0.02 | 0.02 | 0.00 | 0.96 | 0.00 |
| Central Asia | 0.08 | 0.05 | 0.00 | 0.87 | 0.00 | 0.01 | 0.05 | 0.00 | 0.94 | 0.00 |
| Central Europe | 0.05 | 0.02 | 0.00 | 0.93 | 0.00 | 0.01 | 0.01 | 0.00 | 0.98 | 0.00 |
| Central Latin America | 0.03 | 0.02 | 0.00 | 0.95 | 0.00 | 0.00 | 0.02 | 0.00 | 0.97 | 0.00 |
| Central Sub-Saharan Africa | 0.25 | 0.05 | 0.00 | 0.69 | 0.00 | 0.21 | 0.05 | 0.00 | 0.74 | 0.00 |
| East Asia | 0.20 | 0.01 | 0.00 | 0.79 | 0.00 | 0.02 | 0.01 | 0.00 | 0.97 | 0.00 |
| Eastern Europe | 0.04 | 0.04 | 0.00 | 0.91 | 0.01 | 0.00 | 0.04 | 0.00 | 0.95 | 0.00 |
| Eastern Sub-Saharan Africa | 0.16 | 0.04 | 0.00 | 0.79 | 0.00 | 0.07 | 0.04 | 0.00 | 0.89 | 0.00 |
| High-income Asia Pacific | 0.08 | 0.01 | 0.00 | 0.91 | 0.00 | 0.05 | 0.01 | 0.00 | 0.94 | 0.00 |
| High-income North America | 0.02 | 0.02 | 0.00 | 0.95 | 0.00 | 0.01 | 0.02 | 0.00 | 0.97 | 0.00 |
| North Africa and Middle East | 0.04 | 0.01 | 0.00 | 0.94 | 0.00 | 0.01 | 0.01 | 0.00 | 0.98 | 0.00 |
| Oceania | 0.17 | 0.02 | 0.00 | 0.81 | 0.00 | 0.06 | 0.02 | 0.00 | 0.92 | 0.00 |
| South Asia | 0.09 | 0.02 | 0.00 | 0.88 | 0.01 | 0.06 | 0.02 | 0.00 | 0.92 | 0.00 |
| Southeast Asia | 0.13 | 0.04 | 0.00 | 0.83 | 0.00 | 0.04 | 0.03 | 0.00 | 0.92 | 0.00 |
| Southern Latin America | 0.03 | 0.01 | 0.00 | 0.96 | 0.00 | 0.00 | 0.01 | 0.00 | 0.98 | 0.00 |
| Southern Sub-Saharan Africa | 0.12 | 0.03 | 0.00 | 0.85 | 0.00 | 0.03 | 0.02 | 0.00 | 0.94 | 0.00 |
| Tropical Latin America | 0.06 | 0.03 | 0.00 | 0.91 | 0.00 | 0.00 | 0.03 | 0.00 | 0.97 | 0.00 |
| Western Europe | 0.03 | 0.02 | 0.00 | 0.95 | 0.00 | 0.01 | 0.01 | 0.00 | 0.97 | 0.00 |
| Western Sub-Saharan Africa | 0.21 | 0.05 | 0.00 | 0.74 | 0.00 | 0.12 | 0.04 | 0.00 | 0.84 | 0.00 |
| **20-24years** |  |  |  |  |  |  |  |  |  |  |
| Global | 0.09 | 0.02 | 0.00 | 0.88 | 0.00 | 0.05 | 0.02 | 0.00 | 0.00 | 0.93 |
| High SDI | 0.06 | 0.02 | 0.00 | 0.91 | 0.00 | 0.02 | 0.02 | 0.00 | 0.00 | 0.97 |
| High-middle SDI | 0.10 | 0.02 | 0.00 | 0.88 | 0.00 | 0.02 | 0.01 | 0.00 | 0.00 | 0.97 |
| Middle SDI | 0.09 | 0.02 | 0.00 | 0.89 | 0.00 | 0.04 | 0.02 | 0.00 | 0.00 | 0.94 |
| Low-middle SDI | 0.08 | 0.03 | 0.00 | 0.89 | 0.00 | 0.05 | 0.02 | 0.00 | 0.00 | 0.93 |
| Low SDI | 0.14 | 0.04 | 0.00 | 0.82 | 0.00 | 0.11 | 0.03 | 0.00 | 0.00 | 0.86 |
| Andean Latin America | 0.03 | 0.01 | 0.00 | 0.95 | 0.00 | 0.02 | 0.01 | 0.00 | 0.00 | 0.96 |
| Australasia | 0.08 | 0.03 | 0.00 | 0.89 | 0.00 | 0.01 | 0.02 | 0.00 | 0.00 | 0.97 |
| Caribbean | 0.03 | 0.02 | 0.00 | 0.94 | 0.00 | 0.02 | 0.02 | 0.00 | 0.00 | 0.96 |
| Central Asia | 0.07 | 0.04 | 0.00 | 0.88 | 0.00 | 0.05 | 0.04 | 0.00 | 0.01 | 0.91 |
| Central Europe | 0.05 | 0.02 | 0.00 | 0.93 | 0.00 | 0.01 | 0.01 | 0.00 | 0.00 | 0.98 |
| Central Latin America | 0.03 | 0.02 | 0.00 | 0.95 | 0.00 | 0.01 | 0.02 | 0.00 | 0.00 | 0.97 |
| Central Sub-Saharan Africa | 0.21 | 0.05 | 0.00 | 0.73 | 0.00 | 0.19 | 0.05 | 0.00 | 0.00 | 0.76 |
| East Asia | 0.17 | 0.01 | 0.00 | 0.82 | 0.00 | 0.04 | 0.01 | 0.00 | 0.00 | 0.95 |
| Eastern Europe | 0.04 | 0.04 | 0.00 | 0.92 | 0.01 | 0.01 | 0.04 | 0.00 | 0.01 | 0.94 |
| Eastern Sub-Saharan Africa | 0.13 | 0.04 | 0.00 | 0.83 | 0.00 | 0.10 | 0.03 | 0.00 | 0.00 | 0.87 |
| High-income Asia Pacific | 0.13 | 0.02 | 0.00 | 0.85 | 0.00 | 0.06 | 0.02 | 0.00 | 0.00 | 0.92 |
| High-income North America | 0.02 | 0.04 | 0.00 | 0.94 | 0.00 | 0.01 | 0.02 | 0.00 | 0.00 | 0.97 |
| North Africa and Middle East | 0.04 | 0.02 | 0.00 | 0.94 | 0.00 | 0.02 | 0.02 | 0.00 | 0.00 | 0.97 |
| Oceania | 0.15 | 0.02 | 0.00 | 0.83 | 0.00 | 0.07 | 0.02 | 0.00 | 0.00 | 0.91 |
| South Asia | 0.07 | 0.02 | 0.00 | 0.91 | 0.00 | 0.05 | 0.01 | 0.00 | 0.00 | 0.93 |
| Southeast Asia | 0.11 | 0.03 | 0.00 | 0.86 | 0.00 | 0.05 | 0.03 | 0.00 | 0.00 | 0.92 |
| Southern Latin America | 0.03 | 0.01 | 0.00 | 0.96 | 0.00 | 0.01 | 0.01 | 0.00 | 0.00 | 0.97 |
| Southern Sub-Saharan Africa | 0.09 | 0.02 | 0.00 | 0.88 | 0.00 | 0.04 | 0.02 | 0.00 | 0.00 | 0.94 |
| Tropical Latin America | 0.04 | 0.03 | 0.00 | 0.93 | 0.00 | 0.01 | 0.02 | 0.00 | 0.00 | 0.97 |
| Western Europe | 0.04 | 0.02 | 0.00 | 0.94 | 0.00 | 0.01 | 0.01 | 0.00 | 0.00 | 0.97 |
| Western Sub-Saharan Africa | 0.17 | 0.04 | 0.00 | 0.78 | 0.00 | 0.13 | 0.03 | 0.00 | 0.00 | 0.83 |
|  |  |  |  |  |  |  |  |  |  |  |

**Supplementary Table 5. Proportional contributions of different etiologies to cirrhosis prevalence among individuals aged 5–24 years in 2000 and 2021, stratified by global, five SDI regions, and 21 GBD regions.**

| **Characteristics** | **2000** | | | | | **2021** | | | | |
| --- | --- | --- | --- | --- | --- | --- | --- | --- | --- | --- |
|  | **Chronic hepatitis B including cirrhosis (%)** | **Chronic hepatitis C including cirrhosis (%)** | **Cirrhosis due to alcohol (%)** | **Metabolic dysfunction-associated fatty liver disease including cirrhosis (%)** | **Cirrhosis due to other causes (%)** | **Chronic hepatitis B including cirrhosis (%)** | **Chronic hepatitis C including cirrhosis (%)** | **Cirrhosis due to alcohol (%)** | **Metabolic dysfunction-associated fatty liver disease including cirrhosis (%)** | **Cirrhosis due to other causes (%)** |
| **5-9years** |  | | | | | | | | | |
| Global | 0.79 | 0.19 | 0.00 | 0.00 | 0.02 | 0.56 | 0.41 | 0.00 | 0.00 | 0.04 |
| High SDI | 0.59 | 0.39 | 0.00 | 0.00 | 0.02 | 0.28 | 0.67 | 0.00 | 0.00 | 0.06 |
| High-middle SDI | 0.82 | 0.18 | 0.00 | 0.00 | 0.01 | 0.27 | 0.70 | 0.00 | 0.00 | 0.03 |
| Middle SDI | 0.80 | 0.19 | 0.00 | 0.00 | 0.02 | 0.39 | 0.57 | 0.00 | 0.00 | 0.04 |
| Low-middle SDI | 0.75 | 0.23 | 0.00 | 0.00 | 0.03 | 0.48 | 0.47 | 0.00 | 0.00 | 0.05 |
| Low SDI | 0.83 | 0.16 | 0.00 | 0.00 | 0.02 | 0.71 | 0.26 | 0.00 | 0.00 | 0.03 |
| Andean Latin America | 0.60 | 0.34 | 0.00 | 0.00 | 0.06 | 0.23 | 0.69 | 0.00 | 0.00 | 0.08 |
| Australasia | 0.66 | 0.34 | 0.00 | 0.00 | 0.00 | 0.24 | 0.75 | 0.00 | 0.00 | 0.01 |
| Caribbean | 0.50 | 0.46 | 0.00 | 0.00 | 0.04 | 0.32 | 0.63 | 0.00 | 0.00 | 0.05 |
| Central Asia | 0.44 | 0.53 | 0.00 | 0.00 | 0.02 | 0.11 | 0.87 | 0.00 | 0.00 | 0.03 |
| Central Europe | 0.53 | 0.42 | 0.00 | 0.00 | 0.05 | 0.28 | 0.65 | 0.00 | 0.00 | 0.08 |
| Central Latin America | 0.61 | 0.37 | 0.00 | 0.00 | 0.02 | 0.29 | 0.68 | 0.00 | 0.00 | 0.03 |
| Central Sub-Saharan Africa | 0.89 | 0.11 | 0.00 | 0.00 | 0.01 | 0.81 | 0.18 | 0.00 | 0.00 | 0.01 |
| East Asia | 0.88 | 0.12 | 0.00 | 0.00 | 0.00 | 0.29 | 0.70 | 0.00 | 0.00 | 0.01 |
| Eastern Europe | 0.58 | 0.40 | 0.00 | 0.00 | 0.02 | 0.16 | 0.81 | 0.00 | 0.00 | 0.03 |
| Eastern Sub-Saharan Africa | 0.84 | 0.14 | 0.00 | 0.00 | 0.01 | 0.66 | 0.31 | 0.00 | 0.00 | 0.03 |
| High-income Asia Pacific | 0.78 | 0.20 | 0.00 | 0.00 | 0.02 | 0.31 | 0.64 | 0.00 | 0.00 | 0.06 |
| High-income North America | 0.41 | 0.57 | 0.00 | 0.00 | 0.02 | 0.23 | 0.67 | 0.00 | 0.00 | 0.10 |
| North Africa and Middle East | 0.70 | 0.27 | 0.00 | 0.00 | 0.03 | 0.42 | 0.53 | 0.00 | 0.00 | 0.05 |
| Oceania | 0.81 | 0.19 | 0.00 | 0.00 | 0.00 | 0.77 | 0.23 | 0.00 | 0.00 | 0.00 |
| South Asia | 0.77 | 0.19 | 0.00 | 0.00 | 0.04 | 0.42 | 0.48 | 0.00 | 0.00 | 0.09 |
| Southeast Asia | 0.68 | 0.30 | 0.00 | 0.00 | 0.02 | 0.41 | 0.56 | 0.00 | 0.00 | 0.03 |
| Southern Latin America | 0.58 | 0.38 | 0.00 | 0.00 | 0.03 | 0.17 | 0.77 | 0.00 | 0.00 | 0.06 |
| Southern Sub-Saharan Africa | 0.80 | 0.18 | 0.00 | 0.00 | 0.02 | 0.50 | 0.46 | 0.00 | 0.00 | 0.04 |
| Tropical Latin America | 0.69 | 0.30 | 0.00 | 0.00 | 0.00 | 0.26 | 0.72 | 0.00 | 0.00 | 0.01 |
| Western Europe | 0.55 | 0.43 | 0.00 | 0.00 | 0.02 | 0.38 | 0.58 | 0.00 | 0.00 | 0.03 |
| Western Sub-Saharan Africa | 0.81 | 0.18 | 0.00 | 0.00 | 0.01 | 0.71 | 0.27 | 0.00 | 0.00 | 0.02 |
| **10-14years** |  | | | | | | | | | |
| Global | 0.79 | 0.20 | 0.00 | 0.00 | 0.02 | 0.57 | 0.40 | 0.00 | 0.00 | 0.03 |
| High SDI | 0.64 | 0.34 | 0.00 | 0.00 | 0.02 | 0.39 | 0.57 | 0.00 | 0.00 | 0.04 |
| High-middle SDI | 0.82 | 0.18 | 0.00 | 0.00 | 0.01 | 0.28 | 0.69 | 0.00 | 0.00 | 0.03 |
| Middle SDI | 0.81 | 0.17 | 0.00 | 0.00 | 0.01 | 0.48 | 0.49 | 0.00 | 0.00 | 0.03 |
| Low-middle SDI | 0.73 | 0.24 | 0.00 | 0.00 | 0.03 | 0.53 | 0.43 | 0.00 | 0.00 | 0.04 |
| Low SDI | 0.81 | 0.18 | 0.00 | 0.00 | 0.01 | 0.69 | 0.28 | 0.00 | 0.00 | 0.02 |
| Andean Latin America | 0.60 | 0.35 | 0.00 | 0.00 | 0.05 | 0.24 | 0.70 | 0.00 | 0.00 | 0.06 |
| Australasia | 0.65 | 0.34 | 0.00 | 0.00 | 0.00 | 0.27 | 0.72 | 0.00 | 0.00 | 0.01 |
| Caribbean | 0.50 | 0.47 | 0.00 | 0.00 | 0.03 | 0.42 | 0.55 | 0.00 | 0.00 | 0.03 |
| Central Asia | 0.43 | 0.55 | 0.00 | 0.00 | 0.02 | 0.09 | 0.89 | 0.00 | 0.00 | 0.02 |
| Central Europe | 0.70 | 0.28 | 0.00 | 0.00 | 0.03 | 0.23 | 0.70 | 0.00 | 0.00 | 0.07 |
| Central Latin America | 0.66 | 0.33 | 0.00 | 0.00 | 0.01 | 0.22 | 0.75 | 0.00 | 0.00 | 0.02 |
| Central Sub-Saharan Africa | 0.87 | 0.13 | 0.00 | 0.00 | 0.01 | 0.75 | 0.24 | 0.00 | 0.00 | 0.01 |
| East Asia | 0.88 | 0.12 | 0.00 | 0.00 | 0.00 | 0.36 | 0.63 | 0.00 | 0.00 | 0.01 |
| Eastern Europe | 0.55 | 0.43 | 0.00 | 0.00 | 0.02 | 0.15 | 0.82 | 0.00 | 0.00 | 0.03 |
| Eastern Sub-Saharan Africa | 0.82 | 0.16 | 0.00 | 0.00 | 0.01 | 0.65 | 0.33 | 0.00 | 0.00 | 0.03 |
| High-income Asia Pacific | 0.80 | 0.18 | 0.00 | 0.00 | 0.02 | 0.77 | 0.21 | 0.00 | 0.00 | 0.02 |
| High-income North America | 0.54 | 0.45 | 0.00 | 0.00 | 0.02 | 0.22 | 0.70 | 0.00 | 0.00 | 0.08 |
| North Africa and Middle East | 0.74 | 0.23 | 0.00 | 0.00 | 0.02 | 0.40 | 0.55 | 0.00 | 0.00 | 0.05 |
| Oceania | 0.83 | 0.17 | 0.00 | 0.00 | 0.00 | 0.71 | 0.28 | 0.00 | 0.00 | 0.00 |
| South Asia | 0.75 | 0.21 | 0.00 | 0.00 | 0.04 | 0.60 | 0.34 | 0.00 | 0.00 | 0.06 |
| Southeast Asia | 0.73 | 0.25 | 0.00 | 0.00 | 0.02 | 0.48 | 0.49 | 0.00 | 0.00 | 0.02 |
| Southern Latin America | 0.58 | 0.39 | 0.00 | 0.00 | 0.03 | 0.16 | 0.78 | 0.00 | 0.00 | 0.06 |
| Southern Sub-Saharan Africa | 0.82 | 0.17 | 0.00 | 0.00 | 0.01 | 0.53 | 0.44 | 0.00 | 0.00 | 0.03 |
| Tropical Latin America | 0.68 | 0.32 | 0.00 | 0.00 | 0.00 | 0.16 | 0.83 | 0.00 | 0.00 | 0.01 |
| Western Europe | 0.63 | 0.35 | 0.00 | 0.00 | 0.02 | 0.40 | 0.57 | 0.00 | 0.00 | 0.03 |
| Western Sub-Saharan Africa | 0.79 | 0.20 | 0.00 | 0.00 | 0.01 | 0.69 | 0.29 | 0.00 | 0.00 | 0.02 |
| **15-19years** |  | | | | | | | | | |
| Global | 0.47 | 0.13 | 0.00 | 0.39 | 0.01 | 0.28 | 0.15 | 0.00 | 0.56 | 0.01 |
| High SDI | 0.25 | 0.10 | 0.00 | 0.64 | 0.00 | 0.07 | 0.09 | 0.00 | 0.83 | 0.01 |
| High-middle SDI | 0.48 | 0.11 | 0.00 | 0.41 | 0.00 | 0.08 | 0.15 | 0.00 | 0.76 | 0.01 |
| Middle SDI | 0.49 | 0.12 | 0.00 | 0.39 | 0.01 | 0.21 | 0.15 | 0.00 | 0.63 | 0.01 |
| Low-middle SDI | 0.43 | 0.15 | 0.00 | 0.40 | 0.01 | 0.26 | 0.16 | 0.00 | 0.56 | 0.01 |
| Low SDI | 0.60 | 0.14 | 0.00 | 0.25 | 0.01 | 0.49 | 0.15 | 0.00 | 0.35 | 0.01 |
| Andean Latin America | 0.18 | 0.11 | 0.00 | 0.70 | 0.01 | 0.07 | 0.10 | 0.00 | 0.82 | 0.01 |
| Australasia | 0.34 | 0.18 | 0.00 | 0.49 | 0.00 | 0.07 | 0.18 | 0.00 | 0.74 | 0.00 |
| Caribbean | 0.22 | 0.19 | 0.00 | 0.57 | 0.01 | 0.13 | 0.19 | 0.00 | 0.67 | 0.01 |
| Central Asia | 0.27 | 0.36 | 0.00 | 0.36 | 0.01 | 0.05 | 0.42 | 0.00 | 0.51 | 0.01 |
| Central Europe | 0.27 | 0.11 | 0.00 | 0.61 | 0.01 | 0.04 | 0.12 | 0.00 | 0.83 | 0.01 |
| Central Latin America | 0.26 | 0.14 | 0.00 | 0.60 | 0.00 | 0.05 | 0.16 | 0.00 | 0.79 | 0.00 |
| Central Sub-Saharan Africa | 0.74 | 0.12 | 0.00 | 0.14 | 0.00 | 0.70 | 0.12 | 0.00 | 0.18 | 0.00 |
| East Asia | 0.63 | 0.09 | 0.00 | 0.28 | 0.00 | 0.16 | 0.16 | 0.00 | 0.68 | 0.00 |
| Eastern Europe | 0.25 | 0.22 | 0.00 | 0.51 | 0.01 | 0.02 | 0.29 | 0.00 | 0.68 | 0.01 |
| Eastern Sub-Saharan Africa | 0.59 | 0.13 | 0.00 | 0.27 | 0.01 | 0.39 | 0.16 | 0.00 | 0.44 | 0.01 |
| High-income Asia Pacific | 0.27 | 0.06 | 0.00 | 0.66 | 0.01 | 0.20 | 0.05 | 0.00 | 0.74 | 0.01 |
| High-income North America | 0.12 | 0.12 | 0.00 | 0.76 | 0.00 | 0.04 | 0.09 | 0.00 | 0.86 | 0.01 |
| North Africa and Middle East | 0.27 | 0.09 | 0.00 | 0.63 | 0.01 | 0.08 | 0.09 | 0.00 | 0.82 | 0.01 |
| Oceania | 0.64 | 0.11 | 0.00 | 0.24 | 0.00 | 0.36 | 0.20 | 0.00 | 0.44 | 0.00 |
| South Asia | 0.42 | 0.12 | 0.00 | 0.44 | 0.02 | 0.34 | 0.12 | 0.00 | 0.53 | 0.02 |
| Southeast Asia | 0.48 | 0.18 | 0.00 | 0.34 | 0.01 | 0.22 | 0.23 | 0.00 | 0.53 | 0.01 |
| Southern Latin America | 0.13 | 0.09 | 0.00 | 0.78 | 0.01 | 0.02 | 0.09 | 0.00 | 0.88 | 0.01 |
| Southern Sub-Saharan Africa | 0.47 | 0.11 | 0.00 | 0.41 | 0.01 | 0.21 | 0.14 | 0.00 | 0.63 | 0.01 |
| Tropical Latin America | 0.30 | 0.15 | 0.00 | 0.54 | 0.00 | 0.03 | 0.20 | 0.00 | 0.77 | 0.00 |
| Western Europe | 0.18 | 0.10 | 0.00 | 0.71 | 0.01 | 0.07 | 0.09 | 0.00 | 0.84 | 0.00 |
| Western Sub-Saharan Africa | 0.62 | 0.17 | 0.00 | 0.20 | 0.01 | 0.49 | 0.18 | 0.00 | 0.32 | 0.01 |
| **20-24years** |  |  |  |  |  |  |  |  |  |  |
| Global | 0.35 | 0.10 | 0.00 | 0.55 | 0.00 | 0.23 | 0.10 | 0.00 | 0.67 | 0.00 |
| High SDI | 0.20 | 0.08 | 0.00 | 0.72 | 0.00 | 0.06 | 0.07 | 0.00 | 0.87 | 0.00 |
| High-middle SDI | 0.36 | 0.09 | 0.00 | 0.55 | 0.00 | 0.11 | 0.09 | 0.00 | 0.80 | 0.00 |
| Middle SDI | 0.36 | 0.09 | 0.00 | 0.55 | 0.00 | 0.19 | 0.09 | 0.00 | 0.71 | 0.00 |
| Low-middle SDI | 0.30 | 0.12 | 0.00 | 0.57 | 0.01 | 0.22 | 0.10 | 0.00 | 0.67 | 0.01 |
| Low SDI | 0.46 | 0.12 | 0.00 | 0.41 | 0.00 | 0.41 | 0.10 | 0.00 | 0.48 | 0.00 |
| Andean Latin America | 0.12 | 0.07 | 0.00 | 0.80 | 0.01 | 0.09 | 0.06 | 0.00 | 0.85 | 0.00 |
| Australasia | 0.26 | 0.13 | 0.00 | 0.60 | 0.00 | 0.05 | 0.12 | 0.00 | 0.83 | 0.00 |
| Caribbean | 0.14 | 0.13 | 0.00 | 0.73 | 0.00 | 0.09 | 0.12 | 0.00 | 0.79 | 0.00 |
| Central Asia | 0.21 | 0.29 | 0.00 | 0.49 | 0.01 | 0.17 | 0.27 | 0.00 | 0.55 | 0.01 |
| Central Europe | 0.19 | 0.08 | 0.00 | 0.72 | 0.00 | 0.04 | 0.08 | 0.00 | 0.88 | 0.00 |
| Central Latin America | 0.16 | 0.10 | 0.00 | 0.74 | 0.00 | 0.05 | 0.10 | 0.00 | 0.84 | 0.00 |
| Central Sub-Saharan Africa | 0.64 | 0.11 | 0.00 | 0.25 | 0.00 | 0.60 | 0.10 | 0.00 | 0.30 | 0.00 |
| East Asia | 0.51 | 0.07 | 0.00 | 0.42 | 0.00 | 0.21 | 0.08 | 0.00 | 0.70 | 0.00 |
| Eastern Europe | 0.18 | 0.18 | 0.00 | 0.63 | 0.01 | 0.08 | 0.19 | 0.00 | 0.71 | 0.01 |
| Eastern Sub-Saharan Africa | 0.46 | 0.11 | 0.00 | 0.43 | 0.00 | 0.38 | 0.10 | 0.00 | 0.51 | 0.00 |
| High-income Asia Pacific | 0.32 | 0.05 | 0.00 | 0.63 | 0.00 | 0.17 | 0.04 | 0.00 | 0.78 | 0.00 |
| High-income North America | 0.08 | 0.08 | 0.00 | 0.83 | 0.00 | 0.03 | 0.07 | 0.00 | 0.90 | 0.00 |
| North Africa and Middle East | 0.18 | 0.06 | 0.00 | 0.76 | 0.00 | 0.08 | 0.06 | 0.00 | 0.86 | 0.00 |
| Oceania | 0.54 | 0.10 | 0.00 | 0.36 | 0.00 | 0.35 | 0.14 | 0.00 | 0.51 | 0.00 |
| South Asia | 0.28 | 0.09 | 0.00 | 0.63 | 0.01 | 0.23 | 0.08 | 0.00 | 0.69 | 0.01 |
| Southeast Asia | 0.36 | 0.14 | 0.00 | 0.49 | 0.00 | 0.21 | 0.15 | 0.00 | 0.63 | 0.00 |
| Southern Latin America | 0.08 | 0.05 | 0.00 | 0.87 | 0.00 | 0.04 | 0.05 | 0.00 | 0.90 | 0.00 |
| Southern Sub-Saharan Africa | 0.33 | 0.08 | 0.00 | 0.58 | 0.00 | 0.19 | 0.09 | 0.00 | 0.72 | 0.00 |
| Tropical Latin America | 0.19 | 0.11 | 0.00 | 0.71 | 0.00 | 0.04 | 0.12 | 0.00 | 0.84 | 0.00 |
| Western Europe | 0.14 | 0.07 | 0.00 | 0.78 | 0.00 | 0.05 | 0.06 | 0.00 | 0.89 | 0.00 |
| Western Sub-Saharan Africa | 0.50 | 0.15 | 0.00 | 0.34 | 0.00 | 0.45 | 0.12 | 0.00 | 0.43 | 0.00 |
|  |  |  |  |  |  |  |  |  |  |  |
|  |  |  |  |  |  |  |  |  |  |  |

**Supplementary Table 6. Proportional contributions of different etiologies to cirrhosis deaths among individuals aged 5–24 years in 2000 and 2021, stratified by global, five SDI regions, and 21 GBD regions.**

| **Characteristics** | **2000** | | | | | **2021** | | | | |
| --- | --- | --- | --- | --- | --- | --- | --- | --- | --- | --- |
|  | **Chronic hepatitis B including cirrhosis (%)** | **Chronic hepatitis C including cirrhosis (%)** | **Cirrhosis due to alcohol (%)** | **Metabolic dysfunction-associated fatty liver disease including cirrhosis (%)** | **Cirrhosis due to other causes (%)** | **Chronic hepatitis B including cirrhosis (%)** | **Chronic hepatitis C including cirrhosis (%)** | **Cirrhosis due to alcohol (%)** | **Metabolic dysfunction-associated fatty liver disease including cirrhosis (%)** | **Cirrhosis due to other causes (%)** |
| **5-9years** |  | | | | | | | | | |
| Global | 0.06 | 0.03 | 0.00 | 0.00 | 0.91 | 0.05 | 0.04 | 0.00 | 0.00 | 0.91 |
| High SDI | 0.06 | 0.04 | 0.00 | 0.00 | 0.90 | 0.04 | 0.04 | 0.00 | 0.00 | 0.92 |
| High-middle SDI | 0.14 | 0.03 | 0.00 | 0.00 | 0.83 | 0.10 | 0.03 | 0.00 | 0.00 | 0.87 |
| Middle SDI | 0.08 | 0.03 | 0.00 | 0.00 | 0.89 | 0.05 | 0.03 | 0.00 | 0.00 | 0.92 |
| Low-middle SDI | 0.04 | 0.03 | 0.00 | 0.00 | 0.93 | 0.05 | 0.04 | 0.00 | 0.00 | 0.91 |
| Low SDI | 0.05 | 0.03 | 0.00 | 0.00 | 0.91 | 0.06 | 0.04 | 0.00 | 0.00 | 0.90 |
| Andean Latin America | 0.01 | 0.01 | 0.00 | 0.00 | 0.98 | 0.01 | 0.01 | 0.00 | 0.00 | 0.98 |
| Australasia | 0.04 | 0.09 | 0.00 | 0.00 | 0.87 | 0.04 | 0.09 | 0.00 | 0.00 | 0.87 |
| Caribbean | 0.01 | 0.02 | 0.00 | 0.00 | 0.96 | 0.02 | 0.03 | 0.00 | 0.00 | 0.96 |
| Central Asia | 0.02 | 0.02 | 0.00 | 0.00 | 0.96 | 0.02 | 0.02 | 0.00 | 0.00 | 0.96 |
| Central Europe | 0.05 | 0.03 | 0.00 | 0.00 | 0.92 | 0.04 | 0.03 | 0.00 | 0.00 | 0.93 |
| Central Latin America | 0.01 | 0.04 | 0.00 | 0.00 | 0.96 | 0.01 | 0.04 | 0.00 | 0.00 | 0.96 |
| Central Sub-Saharan Africa | 0.05 | 0.05 | 0.00 | 0.00 | 0.89 | 0.05 | 0.05 | 0.00 | 0.00 | 0.90 |
| East Asia | 0.45 | 0.04 | 0.00 | 0.00 | 0.51 | 0.40 | 0.04 | 0.00 | 0.00 | 0.56 |
| Eastern Europe | 0.01 | 0.01 | 0.00 | 0.00 | 0.98 | 0.01 | 0.01 | 0.00 | 0.00 | 0.98 |
| Eastern Sub-Saharan Africa | 0.06 | 0.05 | 0.00 | 0.00 | 0.89 | 0.06 | 0.04 | 0.00 | 0.00 | 0.90 |
| High-income Asia Pacific | 0.06 | 0.05 | 0.00 | 0.00 | 0.89 | 0.04 | 0.07 | 0.00 | 0.00 | 0.89 |
| High-income North America | 0.01 | 0.04 | 0.00 | 0.00 | 0.95 | 0.00 | 0.04 | 0.00 | 0.00 | 0.96 |
| North Africa and Middle East | 0.03 | 0.04 | 0.00 | 0.00 | 0.93 | 0.03 | 0.04 | 0.00 | 0.00 | 0.92 |
| Oceania | 0.17 | 0.05 | 0.00 | 0.00 | 0.78 | 0.16 | 0.06 | 0.00 | 0.00 | 0.78 |
| South Asia | 0.04 | 0.03 | 0.00 | 0.00 | 0.94 | 0.04 | 0.04 | 0.00 | 0.00 | 0.92 |
| Southeast Asia | 0.04 | 0.04 | 0.00 | 0.00 | 0.92 | 0.04 | 0.04 | 0.00 | 0.00 | 0.93 |
| Southern Latin America | 0.02 | 0.04 | 0.00 | 0.00 | 0.95 | 0.02 | 0.04 | 0.00 | 0.00 | 0.94 |
| Southern Sub-Saharan Africa | 0.04 | 0.04 | 0.00 | 0.00 | 0.93 | 0.04 | 0.04 | 0.00 | 0.00 | 0.93 |
| Tropical Latin America | 0.05 | 0.07 | 0.00 | 0.00 | 0.88 | 0.04 | 0.06 | 0.00 | 0.00 | 0.89 |
| Western Europe | 0.01 | 0.02 | 0.00 | 0.00 | 0.97 | 0.01 | 0.02 | 0.00 | 0.00 | 0.97 |
| Western Sub-Saharan Africa | 0.08 | 0.03 | 0.00 | 0.00 | 0.89 | 0.07 | 0.03 | 0.00 | 0.00 | 0.89 |
| **10-14years** |  | | | | | | | | | |
| Andean Latin America | 0.02 | 0.01 | 0.00 | 0.00 | 0.96 | 0.02 | 0.01 | 0.00 | 0.00 | 0.97 |
| Australasia | 0.03 | 0.07 | 0.00 | 0.00 | 0.90 | 0.03 | 0.07 | 0.00 | 0.00 | 0.90 |
| Caribbean | 0.02 | 0.04 | 0.00 | 0.00 | 0.94 | 0.02 | 0.04 | 0.00 | 0.00 | 0.94 |
| Central Asia | 0.03 | 0.04 | 0.00 | 0.00 | 0.93 | 0.03 | 0.04 | 0.00 | 0.00 | 0.93 |
| Central Europe | 0.07 | 0.05 | 0.00 | 0.00 | 0.87 | 0.07 | 0.05 | 0.00 | 0.00 | 0.88 |
| Central Latin America | 0.01 | 0.06 | 0.00 | 0.00 | 0.93 | 0.01 | 0.06 | 0.00 | 0.00 | 0.93 |
| Central Sub-Saharan Africa | 0.08 | 0.08 | 0.00 | 0.00 | 0.84 | 0.07 | 0.08 | 0.00 | 0.00 | 0.85 |
| East Asia | 0.55 | 0.04 | 0.00 | 0.00 | 0.40 | 0.50 | 0.05 | 0.00 | 0.00 | 0.45 |
| Eastern Europe | 0.01 | 0.02 | 0.00 | 0.00 | 0.97 | 0.01 | 0.02 | 0.00 | 0.00 | 0.97 |
| Eastern Sub-Saharan Africa | 0.09 | 0.07 | 0.00 | 0.00 | 0.84 | 0.09 | 0.07 | 0.00 | 0.00 | 0.85 |
| Global | 0.08 | 0.05 | 0.00 | 0.00 | 0.87 | 0.07 | 0.06 | 0.00 | 0.00 | 0.87 |
| High SDI | 0.10 | 0.07 | 0.00 | 0.00 | 0.84 | 0.05 | 0.07 | 0.00 | 0.00 | 0.88 |
| High-income Asia Pacific | 0.10 | 0.09 | 0.00 | 0.00 | 0.81 | 0.07 | 0.11 | 0.00 | 0.00 | 0.82 |
| High-income North America | 0.01 | 0.07 | 0.00 | 0.00 | 0.92 | 0.01 | 0.06 | 0.00 | 0.00 | 0.93 |
| High-middle SDI | 0.19 | 0.04 | 0.00 | 0.00 | 0.77 | 0.11 | 0.04 | 0.00 | 0.00 | 0.85 |
| Low SDI | 0.08 | 0.05 | 0.00 | 0.00 | 0.87 | 0.09 | 0.06 | 0.00 | 0.00 | 0.86 |
| Low-middle SDI | 0.06 | 0.05 | 0.00 | 0.00 | 0.89 | 0.06 | 0.06 | 0.00 | 0.00 | 0.88 |
| Middle SDI | 0.11 | 0.05 | 0.00 | 0.00 | 0.84 | 0.07 | 0.05 | 0.00 | 0.00 | 0.88 |
| North Africa and Middle East | 0.06 | 0.06 | 0.00 | 0.00 | 0.88 | 0.05 | 0.07 | 0.00 | 0.00 | 0.88 |
| Oceania | 0.26 | 0.09 | 0.00 | 0.00 | 0.65 | 0.26 | 0.09 | 0.00 | 0.00 | 0.65 |
| South Asia | 0.05 | 0.04 | 0.00 | 0.00 | 0.91 | 0.05 | 0.05 | 0.00 | 0.00 | 0.90 |
| Southeast Asia | 0.07 | 0.06 | 0.00 | 0.00 | 0.87 | 0.06 | 0.06 | 0.00 | 0.00 | 0.88 |
| Southern Latin America | 0.02 | 0.06 | 0.00 | 0.00 | 0.91 | 0.02 | 0.07 | 0.00 | 0.00 | 0.91 |
| Southern Sub-Saharan Africa | 0.06 | 0.06 | 0.00 | 0.00 | 0.88 | 0.06 | 0.06 | 0.00 | 0.00 | 0.88 |
| Tropical Latin America | 0.08 | 0.11 | 0.00 | 0.00 | 0.82 | 0.06 | 0.10 | 0.00 | 0.00 | 0.83 |
| Western Europe | 0.02 | 0.04 | 0.00 | 0.00 | 0.95 | 0.01 | 0.04 | 0.00 | 0.00 | 0.95 |
| Western Sub-Saharan Africa | 0.12 | 0.05 | 0.00 | 0.00 | 0.83 | 0.11 | 0.05 | 0.00 | 0.00 | 0.84 |
| **15-19years** |  | | | | | | | | | |
| Global | 0.11 | 0.07 | 0.00 | 0.01 | 0.01 | 0.09 | 0.07 | 0.00 | 0.01 | 0.82 |
| High SDI | 0.14 | 0.09 | 0.01 | 0.03 | 0.03 | 0.07 | 0.09 | 0.00 | 0.03 | 0.80 |
| High-middle SDI | 0.21 | 0.05 | 0.00 | 0.02 | 0.02 | 0.13 | 0.06 | 0.00 | 0.02 | 0.79 |
| Middle SDI | 0.13 | 0.06 | 0.00 | 0.01 | 0.01 | 0.09 | 0.06 | 0.00 | 0.02 | 0.83 |
| Low-middle SDI | 0.08 | 0.07 | 0.00 | 0.01 | 0.01 | 0.08 | 0.08 | 0.00 | 0.01 | 0.83 |
| Low SDI | 0.11 | 0.07 | 0.00 | 0.01 | 0.01 | 0.11 | 0.08 | 0.00 | 0.01 | 0.79 |
| Andean Latin America | 0.03 | 0.02 | 0.01 | 0.04 | 0.04 | 0.03 | 0.02 | 0.01 | 0.06 | 0.88 |
| Australasia | 0.08 | 0.16 | 0.00 | 0.04 | 0.04 | 0.07 | 0.15 | 0.00 | 0.06 | 0.72 |
| Caribbean | 0.03 | 0.06 | 0.01 | 0.03 | 0.03 | 0.03 | 0.06 | 0.01 | 0.04 | 0.86 |
| Central Asia | 0.04 | 0.06 | 0.01 | 0.02 | 0.02 | 0.04 | 0.06 | 0.01 | 0.03 | 0.87 |
| Central Europe | 0.10 | 0.07 | 0.01 | 0.03 | 0.03 | 0.09 | 0.07 | 0.01 | 0.04 | 0.79 |
| Central Latin America | 0.02 | 0.09 | 0.01 | 0.03 | 0.03 | 0.02 | 0.09 | 0.01 | 0.05 | 0.84 |
| Central Sub-Saharan Africa | 0.11 | 0.11 | 0.00 | 0.01 | 0.01 | 0.10 | 0.11 | 0.00 | 0.01 | 0.78 |
| East Asia | 0.64 | 0.05 | 0.00 | 0.02 | 0.02 | 0.58 | 0.06 | 0.01 | 0.03 | 0.33 |
| Eastern Europe | 0.02 | 0.03 | 0.00 | 0.02 | 0.02 | 0.01 | 0.03 | 0.00 | 0.02 | 0.93 |
| Eastern Sub-Saharan Africa | 0.13 | 0.09 | 0.00 | 0.01 | 0.01 | 0.12 | 0.09 | 0.00 | 0.01 | 0.78 |
| High-income Asia Pacific | 0.13 | 0.13 | 0.01 | 0.02 | 0.02 | 0.09 | 0.15 | 0.01 | 0.02 | 0.73 |
| High-income North America | 0.01 | 0.11 | 0.00 | 0.02 | 0.02 | 0.01 | 0.09 | 0.00 | 0.02 | 0.87 |
| North Africa and Middle East | 0.09 | 0.10 | 0.00 | 0.01 | 0.01 | 0.08 | 0.10 | 0.00 | 0.02 | 0.80 |
| Oceania | 0.30 | 0.10 | 0.01 | 0.03 | 0.03 | 0.29 | 0.10 | 0.01 | 0.03 | 0.57 |
| South Asia | 0.06 | 0.06 | 0.00 | 0.01 | 0.01 | 0.06 | 0.06 | 0.00 | 0.01 | 0.86 |
| Southeast Asia | 0.09 | 0.09 | 0.00 | 0.01 | 0.01 | 0.09 | 0.09 | 0.00 | 0.01 | 0.81 |
| Southern Latin America | 0.04 | 0.09 | 0.00 | 0.03 | 0.03 | 0.04 | 0.10 | 0.00 | 0.04 | 0.82 |
| Southern Sub-Saharan Africa | 0.09 | 0.09 | 0.00 | 0.02 | 0.02 | 0.09 | 0.09 | 0.00 | 0.03 | 0.79 |
| Tropical Latin America | 0.11 | 0.15 | 0.01 | 0.04 | 0.04 | 0.10 | 0.15 | 0.01 | 0.05 | 0.69 |
| Western Europe | 0.02 | 0.05 | 0.01 | 0.04 | 0.04 | 0.02 | 0.05 | 0.01 | 0.05 | 0.87 |
| Western Sub-Saharan Africa | 0.16 | 0.06 | 0.00 | 0.01 | 0.01 | 0.14 | 0.06 | 0.00 | 0.01 | 0.78 |
| **20-24years** |  | | | | | | | | | |
| Global | 0.24 | 0.19 | 0.04 | 0.02 | 0.51 | 0.22 | 0.20 | 0.04 | 0.02 | 0.52 |
| High SDI | 0.21 | 0.23 | 0.06 | 0.04 | 0.46 | 0.13 | 0.24 | 0.05 | 0.05 | 0.53 |
| High-middle SDI | 0.31 | 0.12 | 0.05 | 0.03 | 0.49 | 0.25 | 0.15 | 0.05 | 0.04 | 0.52 |
| Middle SDI | 0.26 | 0.18 | 0.04 | 0.02 | 0.49 | 0.20 | 0.18 | 0.04 | 0.03 | 0.54 |
| Low-middle SDI | 0.21 | 0.21 | 0.04 | 0.01 | 0.54 | 0.19 | 0.21 | 0.03 | 0.02 | 0.54 |
| Low SDI | 0.28 | 0.20 | 0.03 | 0.01 | 0.48 | 0.27 | 0.21 | 0.03 | 0.02 | 0.47 |
| Andean Latin America | 0.09 | 0.07 | 0.10 | 0.08 | 0.66 | 0.07 | 0.06 | 0.10 | 0.12 | 0.64 |
| Australasia | 0.15 | 0.32 | 0.03 | 0.07 | 0.43 | 0.15 | 0.33 | 0.03 | 0.09 | 0.41 |
| Caribbean | 0.08 | 0.17 | 0.08 | 0.06 | 0.61 | 0.08 | 0.17 | 0.09 | 0.07 | 0.59 |
| Central Asia | 0.10 | 0.17 | 0.06 | 0.03 | 0.64 | 0.09 | 0.17 | 0.07 | 0.05 | 0.62 |
| Central Europe | 0.21 | 0.16 | 0.13 | 0.04 | 0.46 | 0.19 | 0.16 | 0.13 | 0.06 | 0.46 |
| Central Latin America | 0.04 | 0.29 | 0.10 | 0.06 | 0.51 | 0.03 | 0.27 | 0.09 | 0.08 | 0.52 |
| Central Sub-Saharan Africa | 0.25 | 0.28 | 0.03 | 0.01 | 0.43 | 0.23 | 0.28 | 0.03 | 0.02 | 0.45 |
| East Asia | 0.83 | 0.07 | 0.01 | 0.01 | 0.08 | 0.77 | 0.08 | 0.01 | 0.02 | 0.12 |
| Eastern Europe | 0.04 | 0.10 | 0.08 | 0.04 | 0.75 | 0.04 | 0.10 | 0.07 | 0.05 | 0.75 |
| Eastern Sub-Saharan Africa | 0.31 | 0.24 | 0.02 | 0.01 | 0.42 | 0.29 | 0.24 | 0.02 | 0.02 | 0.42 |
| High-income Asia Pacific | 0.29 | 0.25 | 0.08 | 0.02 | 0.35 | 0.20 | 0.30 | 0.08 | 0.03 | 0.39 |
| High-income North America | 0.03 | 0.32 | 0.05 | 0.04 | 0.56 | 0.03 | 0.27 | 0.04 | 0.04 | 0.62 |
| North Africa and Middle East | 0.22 | 0.26 | 0.01 | 0.02 | 0.50 | 0.19 | 0.26 | 0.01 | 0.03 | 0.51 |
| Oceania | 0.47 | 0.20 | 0.03 | 0.03 | 0.27 | 0.47 | 0.20 | 0.03 | 0.03 | 0.27 |
| South Asia | 0.18 | 0.17 | 0.05 | 0.01 | 0.59 | 0.17 | 0.18 | 0.04 | 0.01 | 0.60 |
| Southeast Asia | 0.22 | 0.25 | 0.02 | 0.01 | 0.49 | 0.21 | 0.25 | 0.03 | 0.02 | 0.49 |
| Southern Latin America | 0.09 | 0.27 | 0.04 | 0.05 | 0.55 | 0.08 | 0.27 | 0.04 | 0.07 | 0.53 |
| Southern Sub-Saharan Africa | 0.19 | 0.23 | 0.02 | 0.03 | 0.53 | 0.19 | 0.23 | 0.03 | 0.04 | 0.51 |
| Tropical Latin America | 0.22 | 0.31 | 0.13 | 0.04 | 0.30 | 0.20 | 0.31 | 0.14 | 0.06 | 0.30 |
| Western Europe | 0.06 | 0.16 | 0.11 | 0.08 | 0.59 | 0.05 | 0.14 | 0.11 | 0.10 | 0.60 |
| Western Sub-Saharan Africa | 0.35 | 0.17 | 0.02 | 0.02 | 0.44 | 0.34 | 0.17 | 0.02 | 0.02 | 0.45 |

**Supplementary Figure legends:**

**Supplementary Figure 1. Counts and rates of cirrhosis deaths at the global level by sex, 2000–2021.**

**Supplementary Figure 2. Incidence rates of cirrhosis in 2021 across four age groups (5–9, 10–14, 15–19, and 20–24 years) in countries and territories.**

**Supplementary Figure 3. Prevalence rates of cirrhosis in 2021 across four age groups (5–9, 10–14, 15–19, and 20–24 years) in countries and territories.**

**Supplementary Figure 4. Deaths rates of cirrhosis in 2021 across four age groups (5–9, 10–14, 15–19, and 20–24 years) in countries and territories.**

**Supplementary Figure 5. Contributions of different causes to the deaths rates across four age groups in the years 2000 and 2021.**

**Supplementary Figure 6. Contributions of different causes to the incidence and deaths rates across regions across four age groups in the years 2000 and 2021.**

**Supplementary Figure 7. Age, period, and birth cohort effects on cirrhosis incidence in childhood and adolescence according to age–period–cohort (APC) models.**

**Supplementary Figure 8. Analysis of the impact of age, period, and birth cohort on the incidence of hepatitis B virus (HBV)-related cirrhosis in children and adolescents using an age–period–cohort (APC) model.**

**Supplementary Figure 9. Analysis of the impact of age, period, and birth cohort on the incidence of hepatitis C virus (HCV)-related cirrhosis in children and adolescents using an age–period–cohort (APC) model.**

**Supplementary Figure 10. Prediction of the global ASMR of cirrhosis and the number of cases of cirrhosis caused by different aetiologies in children and adolescents from 2022 to 2050.**

**Supplementary Figure 11. Prediction of the global incidence rate, prevalence rate and deaths rate of cirrhosis and the number of cases of cirrhosis caused by different aetiologies in children and adolescents from 2022 to 2050.**

**Supplementary Figure 1. Counts and rates of cirrhosis deaths at the global level by sex, 2000–2021.**


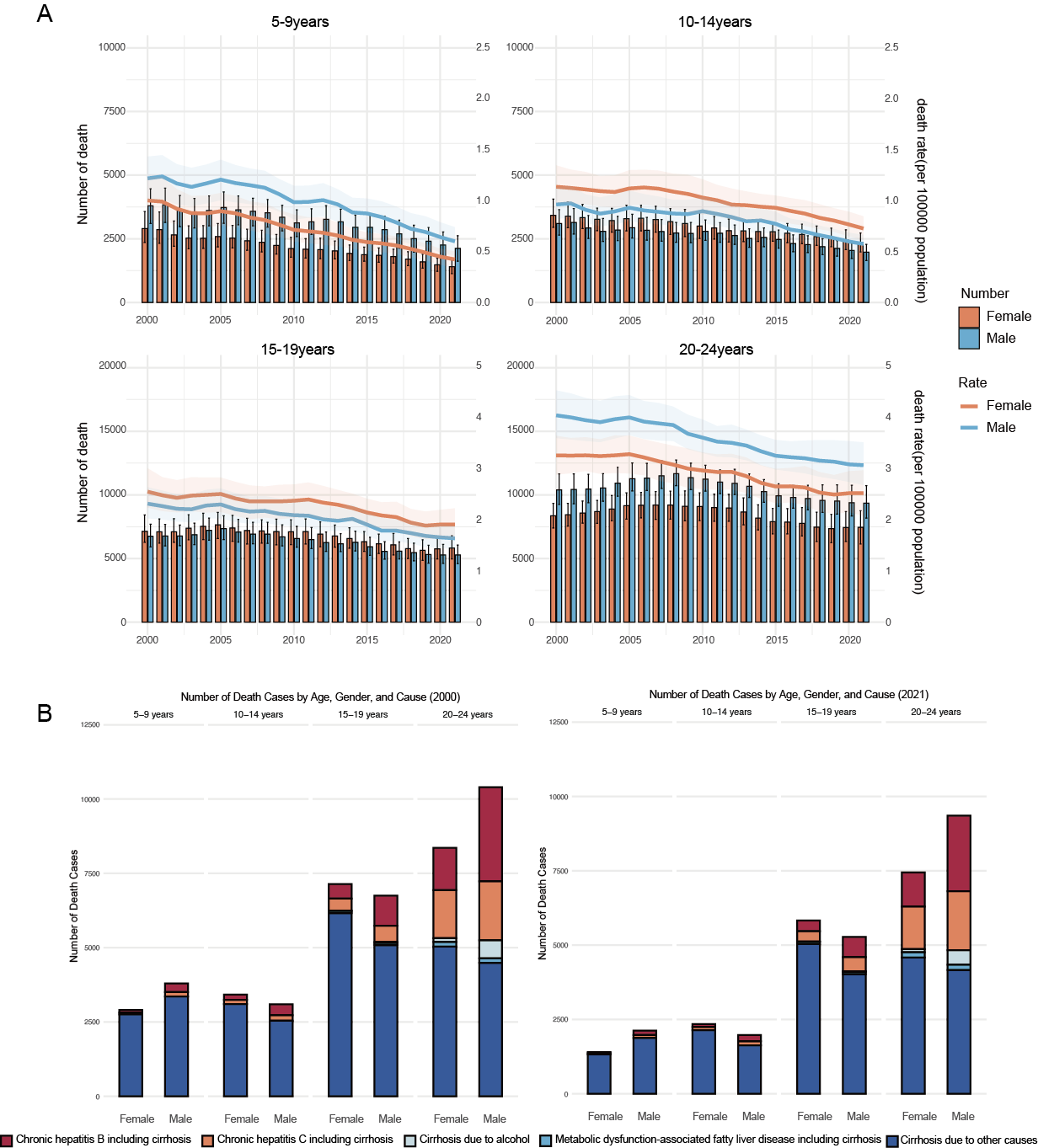


**Supplementary Figure 2. Incidence rates of cirrhosis in 2021 across four age groups (5–9, 10–14, 15–19, and 20–24 years) in countries and territories.**

**
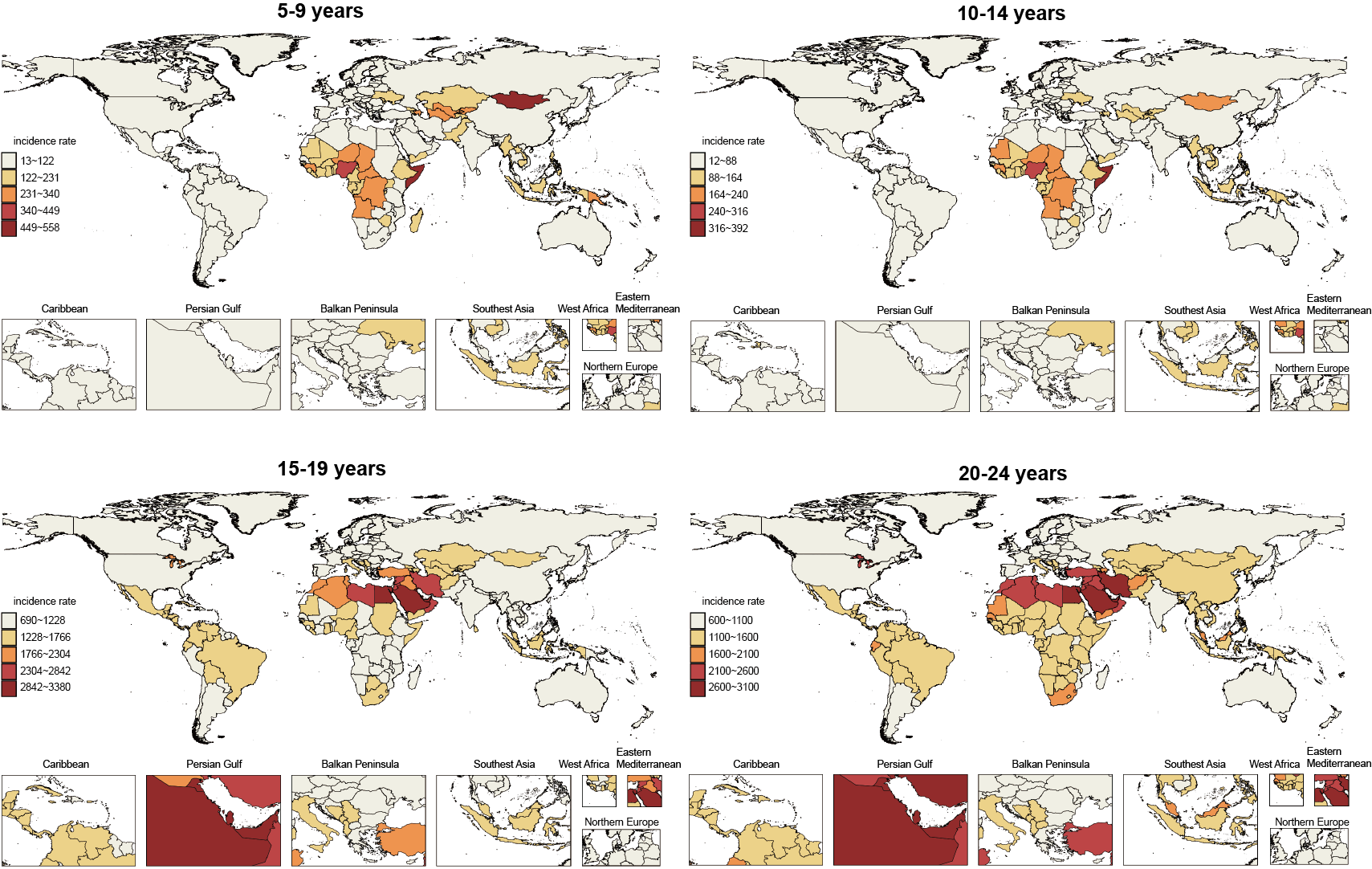
**


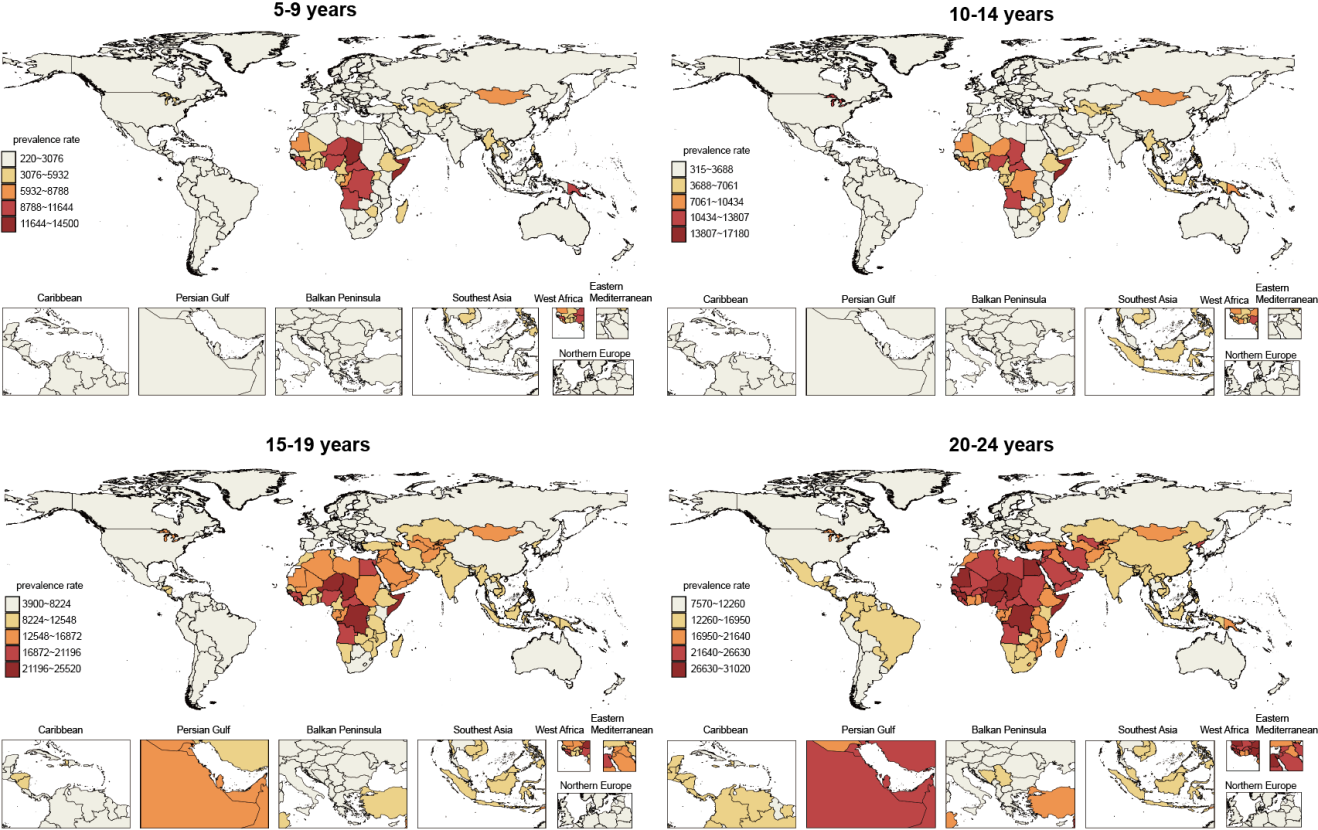
**Supplementary Figure 3. Prevalence rates of cirrhosis in 2021 across four age groups (5–9, 10–14, 15–19, and 20–24 years) in countries and territories.**

**
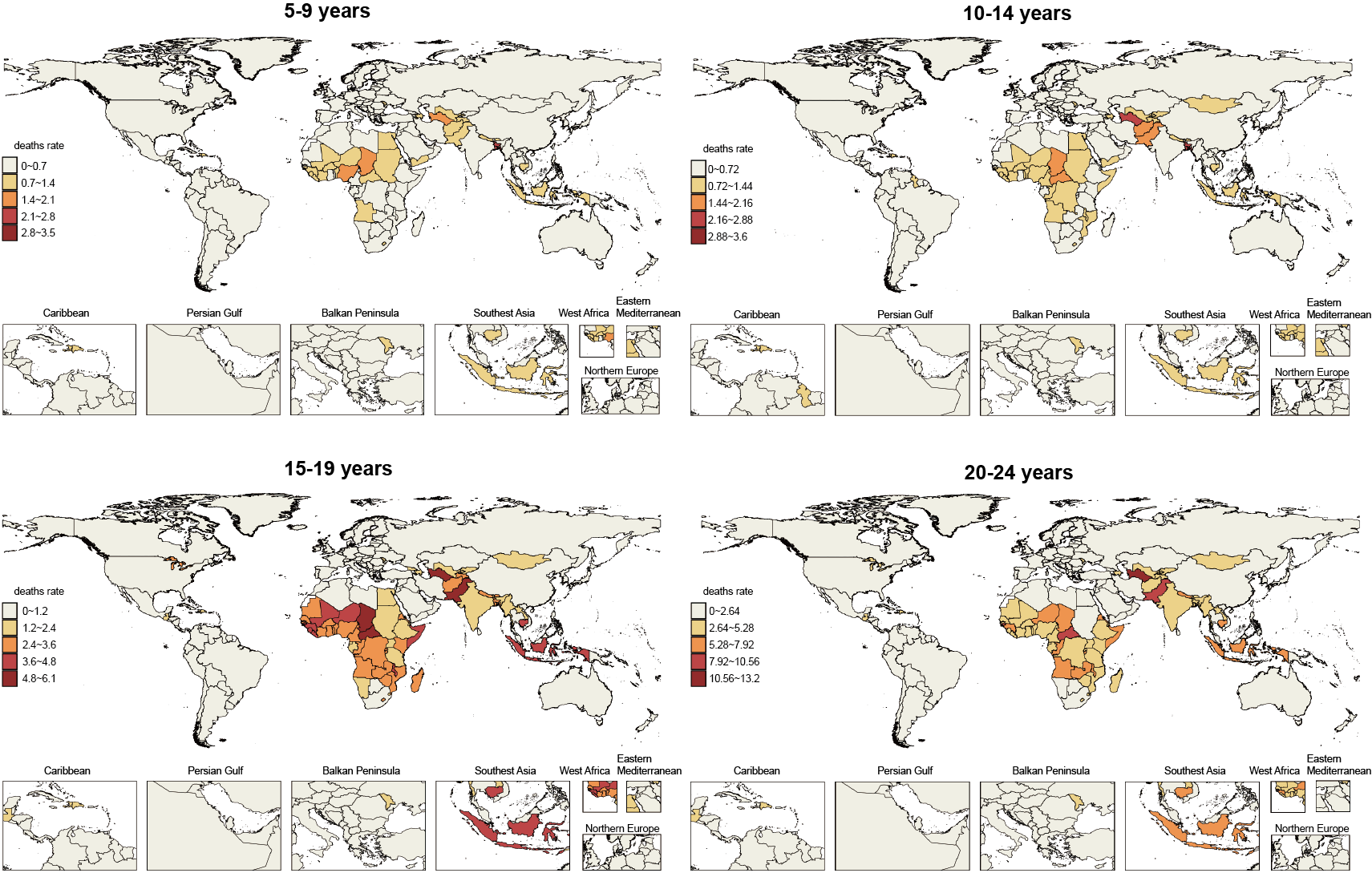
Supplementary Figure 4. Deaths rates of cirrhosis in 2021 across four age groups (5–9, 10–14, 15–19, and 20–24 years) in countries and territories.**

**Supplementary Figure 5. Contributions of different causes to the deaths rates across four age groups in the years 2000 and 2021.**


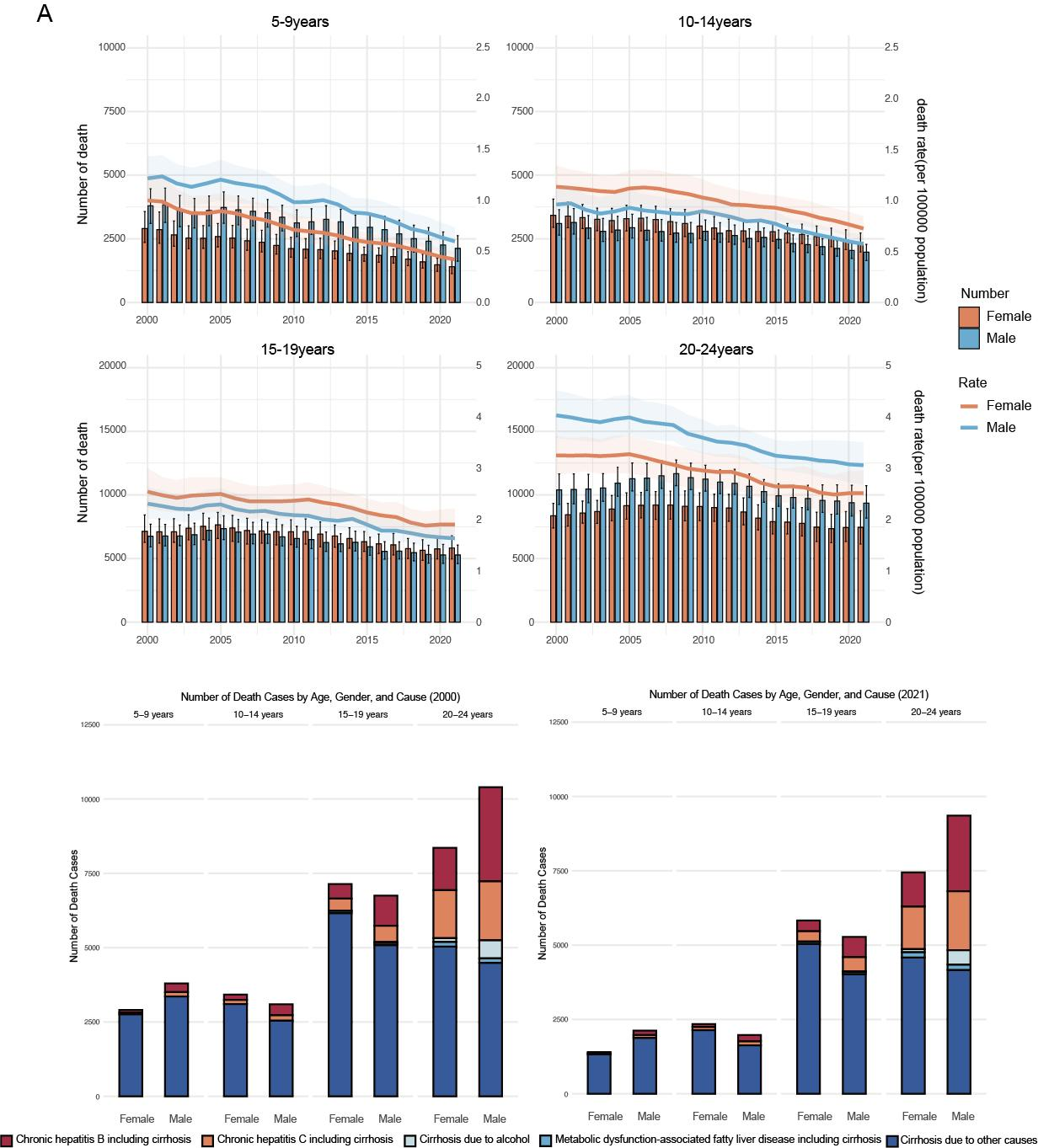


**Supplementary Figure 6. Contributions of different causes to the incidence and deaths rates across regions across four age groups in the years 2000 and 2021.**


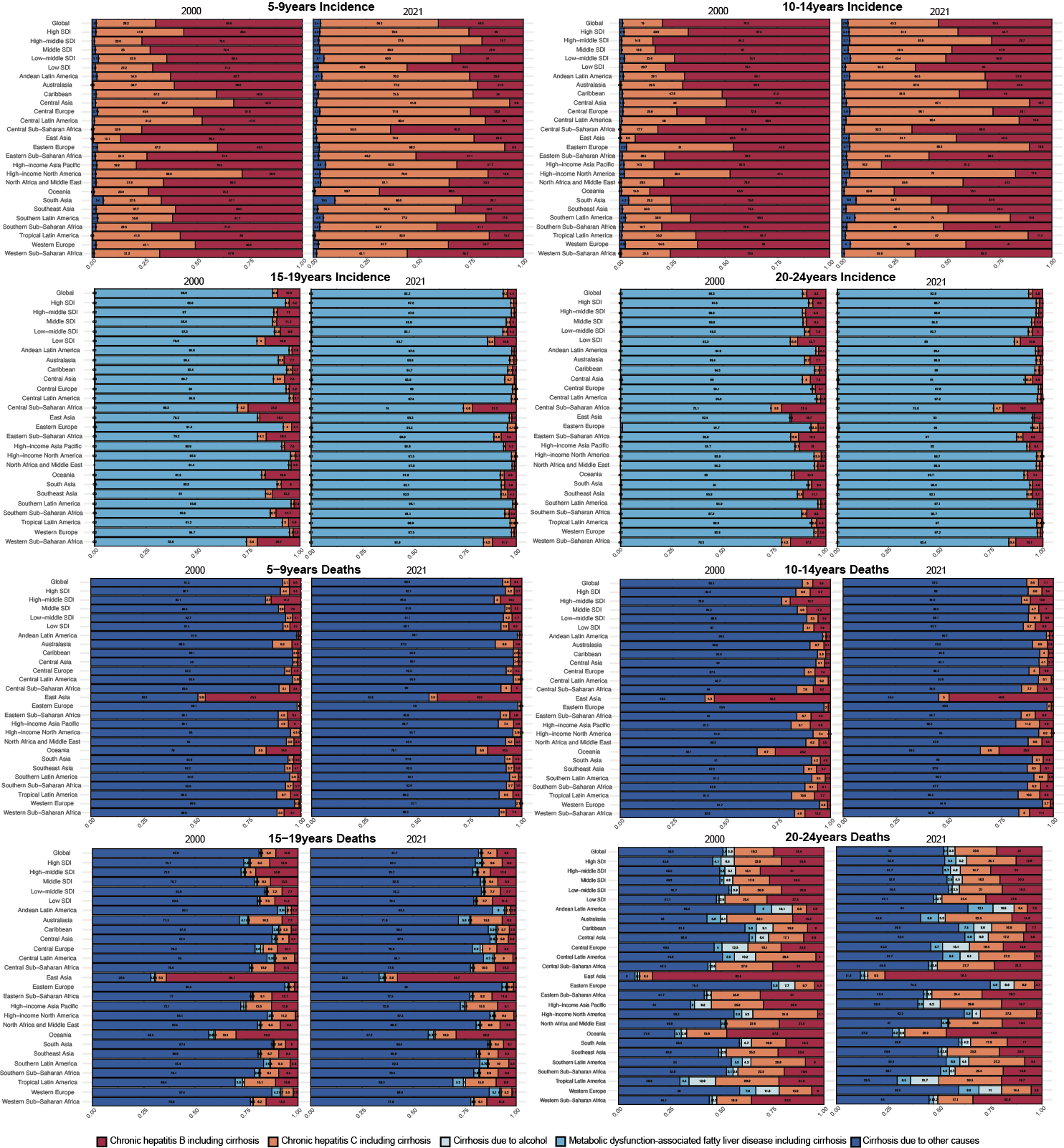


**Supplementary Figure 7. Age, period, and birth cohort effects on cirrhosis incidence in childhood and adolescence according to age–period–cohort (APC) models.
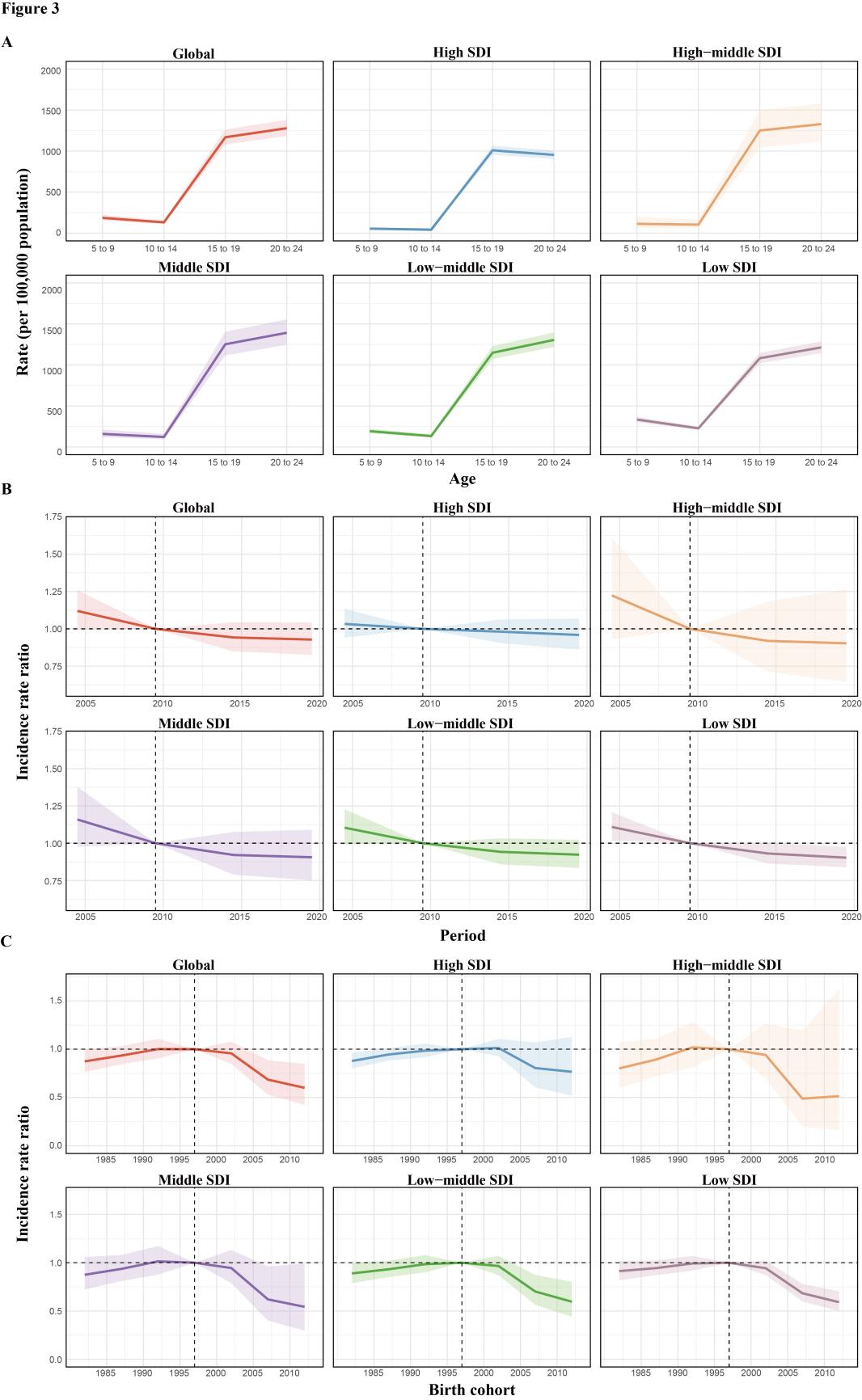
**

**Supplementary Figure 8. Analysis of the impact of age, period, and birth cohort on the incidence of hepatitis B virus (HBV)-related cirrhosis in children and adolescents using an age–period–cohort (APC) model.**


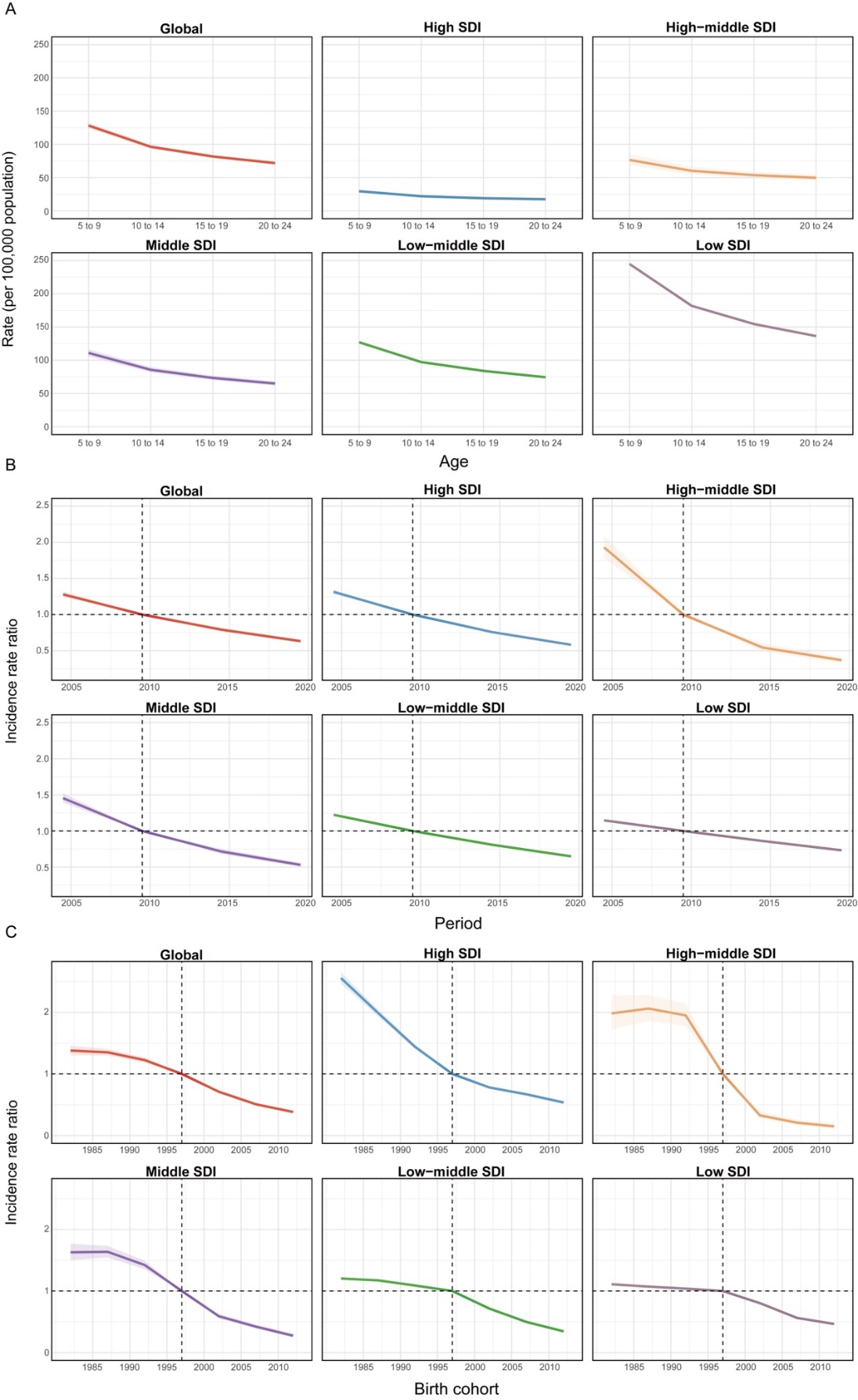


**Supplementary Figure 9. Analysis of the impact of age, period, and birth cohort on the incidence of hepatitis C virus (HCV)-related cirrhosis in children and adolescents using an age–period–cohort (APC) model.**


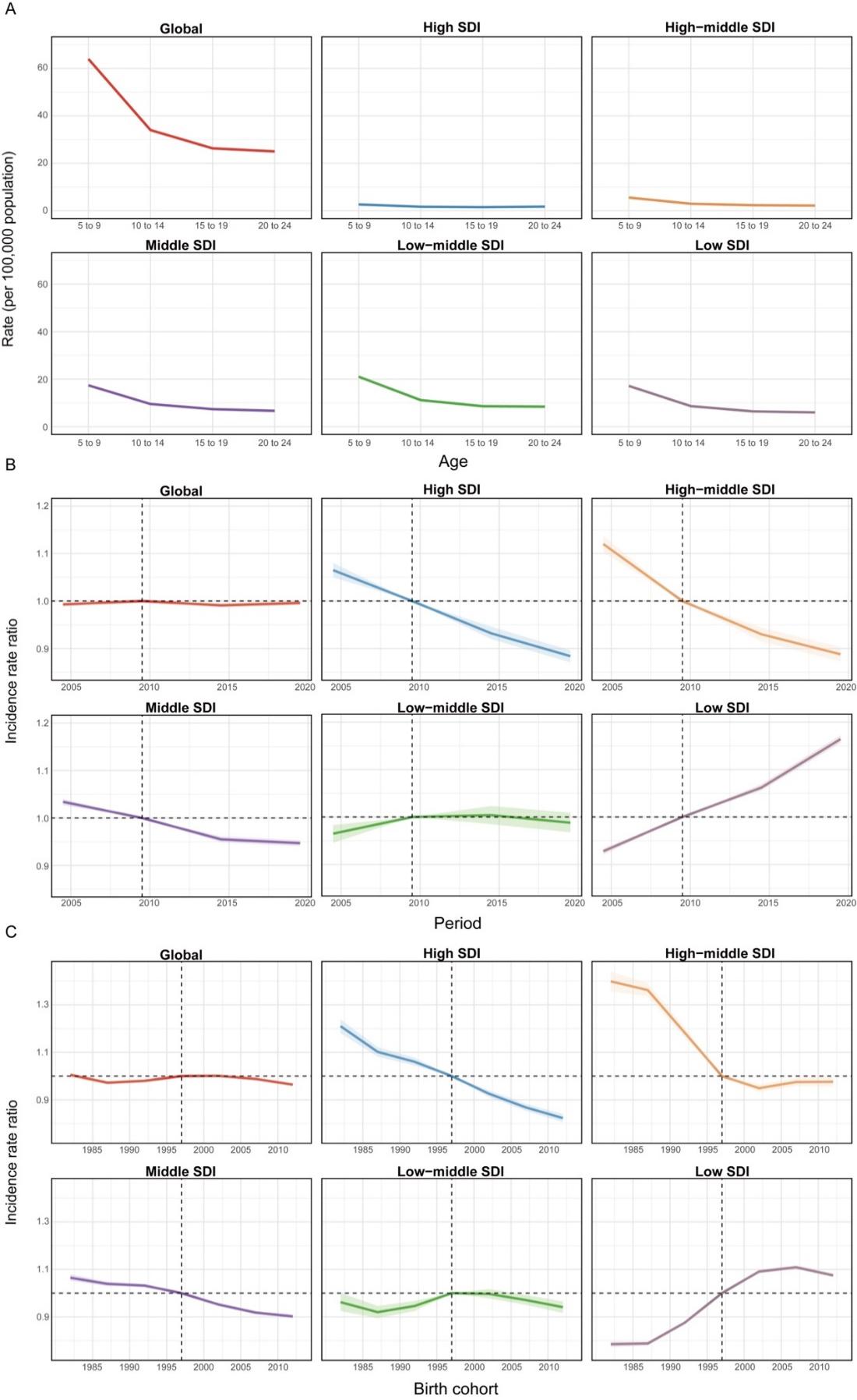


**Supplementary Figure 10. Prediction of the global ASMR of cirrhosis and the number of cases of cirrhosis caused by different aetiologies in children and adolescents from 2022 to 2050.**

**
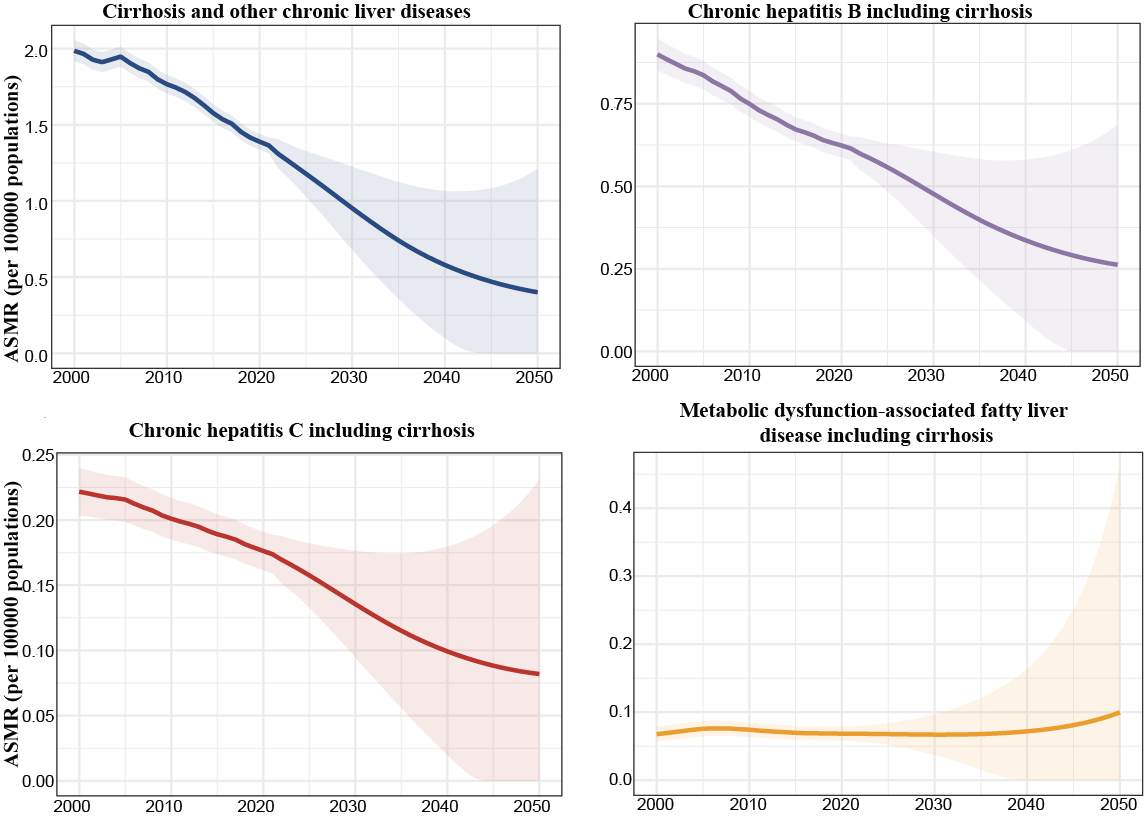
**

**Supplementary Figure 11. Prediction of the global incidence rate, prevalence rate and deaths rate of cirrhosis and the number of cases of cirrhosis caused by different aetiologies in children and adolescents from 2022 to 2050.**

**
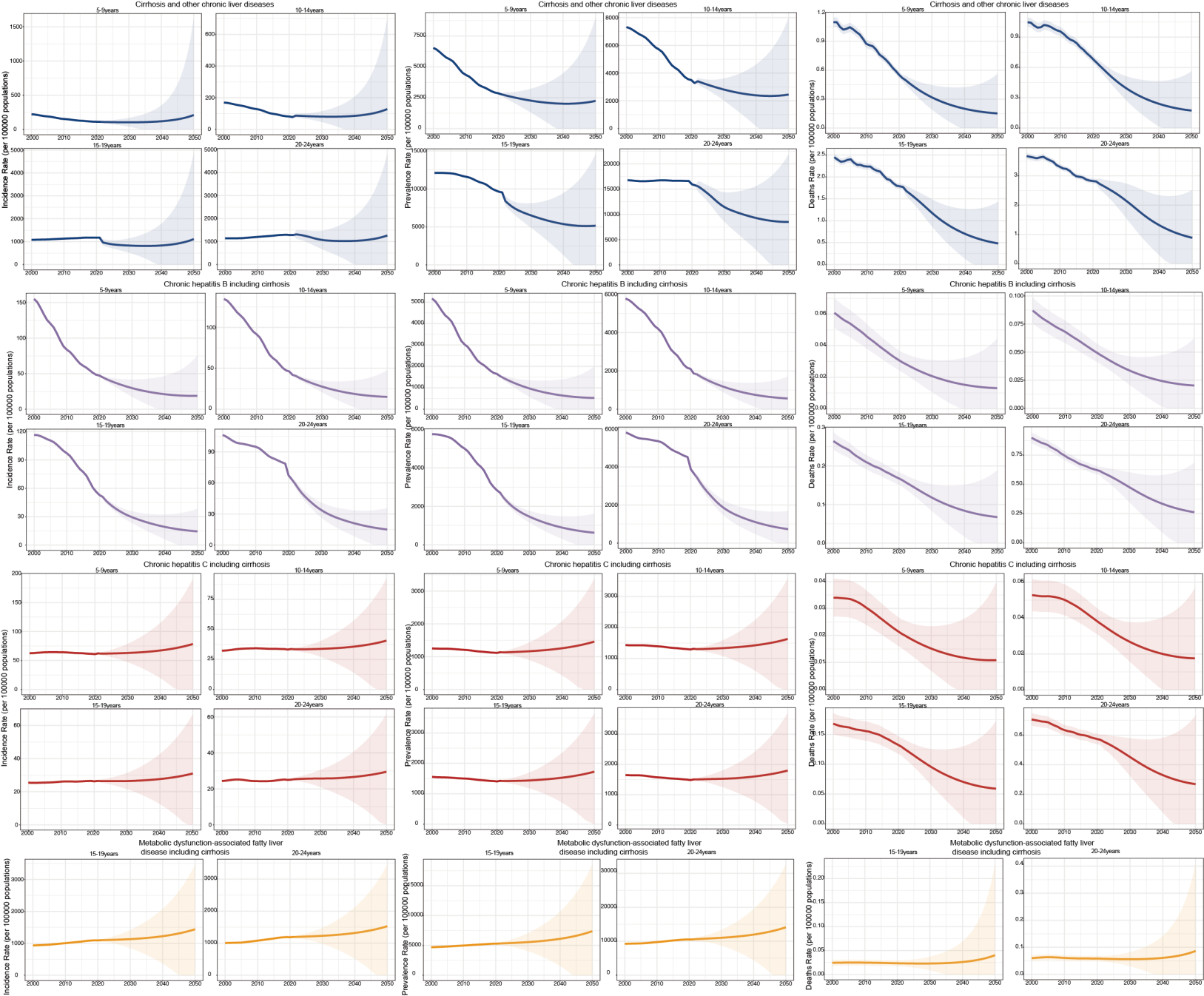
**
